# Prenatal exposure to maternal depression and antidepressants and neurodevelopmental outcomes: A population cohort study

**DOI:** 10.1371/journal.pmed.1004658

**Published:** 2026-09-29

**Authors:** Saraid McIlvride, Neha Rao, Sarjit Singh, Scott M. Nelson, Jill P. Pell, Michael Fleming

**Affiliations:** 1 School of Health and Wellbeing, University of Glasgow, Glasgow, United Kingdom; 2 School of Medicine, Dentistry and Nursing, University of Glasgow, Glasgow, United Kingdom; London School of Hygiene and Tropical Medicine, UNITED KINGDOM OF GREAT BRITAIN AND NORTHERN IRELAND

## Abstract

**Background:**

The long-term effects of antidepressant use in pregnancy on child neurodevelopment are unclear; meanwhile antidepressant use in pregnancy is increasing. We aimed to clarify associations between prenatal maternal depression and/or antidepressant exposure and neurodevelopmental outcomes in school-age children.

**Methods and findings:**

This population-wide cohort study included 167,447 children born in Wales, UK between 2009 and 2016, followed until 2022 (mean age 5.68 years) using linked primary care, maternity and education records. Exposures were one or more antidepressant prescriptions during pregnancy or up to one month before conception, and/or maternal depression diagnosis recorded in the two years before delivery. Outcomes were all-cause and cause-specific special educational need (SEN), including autism spectrum disorder (ASD), sensory impairment, communication difficulties, learning difficulties, physical and medical difficulties, and behavioral-emotional-social difficulties, as well as attention deficit hyperactivity disorder (ADHD). Generalized estimating equation models with an interaction term between maternal depression and antidepressant exposure were used to investigate SEN and causes of SEN. Predicted probabilities and marginal effects were derived to quantify absolute risk. Separate models were fitted for individual antidepressant classes and combinations. Cox proportional hazards regression and flexible parametric survival models were used for time to ADHD diagnosis. Covariates included child sex, area-level deprivation quintile, ethnicity, age, maternal age, smoking, parity, multiple birth and epilepsy.

In total, 12,630 children (7.6%) were exposed to untreated depression during pregnancy; 7,005 (4.2%) to treated depression; and 5,996 (3.6%) to antidepressant medication without a recorded depression diagnosis. SEN was associated with both maternal depression (adjusted odds ratio (aOR) 1.25 (95% CI [1.20,1.31])) and antidepressant medication exposure (aOR 1.46 (95% CI [1.38,1.56])). Predicted probabilities of all-cause SEN were highest among children exposed to both maternal depression and antidepressants (26.5 per 100 children (95% CI [25.5,27.5])) and lowest among unexposed children (20.0 per 100 (95% CI [19.8,20.2])). Depression alone was associated with an increase to 23.6 per 100 children (95% CI [22.9,24.3]), while antidepressant exposure without depression was associated with an increase to 26.3 per 100 (95% CI [25.3,27.4]). Antidepressant exposure was associated with a 6.3% absolute increase in SEN risk (95% CI [5.2,7.4]) among children of mothers without depression and 2.9% absolute increase (95% CI [1.7,4.1]) among children of mothers with depression. Associations with antidepressant exposure were observed across multiple outcomes including ASD (aOR 1.59 (95% CI [1.31,1.92])) and ADHD (adjusted hazard ratio (aHR) 1.89 (95% CI [1.57,2.27])). No antidepressant class showed a markedly different association with SEN risk, although tricyclic antidepressants (TCAs) and serotonin and norepinephrine reuptake inhibitors (SNRIs) had significant differences in absolute risk of SEN when comparing treated and untreated depression (TCAs 8.8% (95% CI [3.2,14.4]); SNRIs 8.8% (95% CI [2.6,15.0])). Key limitations include potential confounding by indication and depression severity, as women prescribed antidepressants likely represent a more severely depressed group.

**Conclusions:**

Offspring of mothers with depression have a higher risk of SEN that cannot be attributed solely to antidepressant exposure. Prenatal exposure to antidepressants may confer a small additional absolute risk, but findings may partly represent confounding by depression severity. Further research is needed.

## Author summary

**Why was this study done?:**

- Antidepressant use in pregnancy is rising, yet information on the effects on child neurodevelopment is limited and existing studies give conflicting results.
- Women should be able to make informed decisions around what medication to take during pregnancy, therefore more research is needed to examine the long-term impact of both depression and antidepressant use in pregnancy.

**What did the researchers do and find?:**

- We linked maternal and child health and education databases, resulting in a cohort of 167,447 children born in Wales between 2009 and 2016.
- We tested for associations between exposure to prenatal depression and/or antidepressant use and special educational need (SEN) in children.
- We found significant associations between child SEN and prenatal exposure to maternal depression (aOR 1.25 (95% CI [1.20,1.31])) and antidepressants (aOR 1.46 (95% CI [1.38,1.56])).
- Antidepressant exposure was associated with a 2.9% increase (95% CI [1.7,4.1]) in absolute risk of SEN among those exposed to depression.

**What do these findings mean?:**

- Treated depression had marginally stronger associations with SEN than untreated depression.
- This difference could be due to severity of depression, since we cannot fully distinguish between the effect of the medication itself and the underlying severity of maternal depression, therefore more research is needed.

## Abstract 3

In this study of more than 167,000 children in Wales, Saraid McIlvride and colleagues examined how prenatal exposure to maternal depression and antidepressants relates to later neurodevelopmental outcomes. The findings suggest that children exposed to maternal depression had higher rates of special educational needs.

## Introduction

Mental health disorders are increasingly prevalent in pregnant women, with depression affecting approximately 17% of pregnancies globally [1]. In high-income countries, maternal mental ill health is the primary cause of death in the first postnatal year and a leading cause of maternal mortality overall [2,3]. Untreated maternal depression is associated with adverse obstetric and neonatal outcomes, including preterm birth, low birth weight, and intrauterine growth restriction, as well as impaired neurodevelopment in offspring [4–8]. Concerns about the potential effects of antidepressant exposure on fetal development often lead to poor medication adherence or discontinuation during pregnancy, potentially exposing both mother and child to the risks of untreated illness.

Studies assessing the long-term effects of prenatal exposure to antidepressant medication have reported conflicting results. Some have reported an increased risk of autism spectrum disorder (ASD) or attention deficit hyperactivity disorder (ADHD) [9–11], or a weak association with antidepressant treatment initiated during pregnancy only [12], while others have found no association or suggested that associations are attributable to underlying maternal depression rather than medication exposure [13–17]. These inconsistencies, coupled with methodological limitations of previous studies, have left clinicians and patients without definitive evidence to guide treatment decisions.

This large population-based cohort study linked detailed maternal health records with long-term child educational outcomes, to examine whether prenatal exposure to maternal depression and/or antidepressant medication is associated with increased risk of special educational need (SEN) during childhood (up to age 12). By examining the impact of both treated and untreated depression, we aimed to disentangle the effects of maternal depression from those of antidepressant exposure while adjusting for key confounding factors.

## Methods

This study was reported using the Strengthening the Reporting of Observational Studies in Epidemiology (STROBE) guideline (S1 Checklist).

### Databases

A Wales-wide retrospective cohort of mother-infant pairs was created by linking, at an individual level, data extracted from the Annual District Birth Extract (ADBE), National Community Child Health Database (NCCHD), Maternity Indicators Data Set (MIDS), Welsh Longitudinal General Practice (WLGP) and Education Wales (EDUW) datasets held by the Secure Anonymised Information Linkage (SAIL) Databank [18–22]. The linked data were retained within the SAIL trusted research environment and analyzed remotely using a virtual private network. Datasets are linked at an individual level using a SAIL unique encrypted identifier, known as the anonymized linking field (ALF). Children’s education records were linked to health datasets via their unique educational identifier (Individual Record Number). Access to the data was approved by the SAIL Databank Information Governance Review Panel (IGRP; study approval number 1089). Individual informed consent was not required for use of anonymized datasets arising from routine public service delivery.

Birth records from the ADBE, NCCHD and MIDS (initiated in 2014) databases provided information on date of birth, gestational age, maternal health information such as age and smoking status, obstetric history, delivery and neonatal outcomes such as birthweight and Apgar score. NCCHD has 100% coverage of children born, resident or treated in Wales since 1987, with data completeness of 91% on average up to 2016. MIDS had on average 94% data completeness in 2016. Primary care data (prescriptions, smoking status and clinical diagnoses) were obtained from the WLGP dataset, which covers approximately 86% of the Welsh population (population 3.1 million). Socioeconomic status was estimated using the Welsh Index of Multiple Deprivation (WIMD), a relative measure of area-based deprivation derived from postal codes using seven domains: income, education, health, community safety, services access, housing, and environment [23]. The Pupil Level Annual School Census (PLASC), part of the EDUW dataset, provided data on SEN status (yes/no), as well as individual causes of SEN and demographics including ethnicity. This annual census collects individual-level and aggregated data from all state-funded nursery, primary, secondary and special schools in Wales, and has a high level of completeness. Data are recorded for each year that a child attends school, therefore multiple school records pertaining to the same child are to be expected. It is estimated that 94% of children in Wales are included in EDUW data, with those missing largely due to being educated in the private sector [24].

### Inclusion criteria and definitions

The study population included children who attended state-funded schools in Wales during any of the academic years 2013/2014–2021/2022, were born in Wales from 2009 onwards, and had linked health and maternal health records (S1 Appendix). Children entered the cohort from birth and were followed until the study end date (2022). Medications were classified according to the British National Formulary (BNF). Medications and diagnoses were identified from maternal primary care records using Read codes version 2 (S1 Appendix), selected from published code lists [25–28] and validated by clinical authors. We defined prenatal antidepressant exposure as the mother receiving at least one antidepressant prescription during pregnancy, or up to one month before conception, to capture medication discontinued early in pregnancy. Maternal depression was defined as having a depression diagnosis recorded in their primary care record in the two years prior to delivery. Mothers were excluded if their primary care records weren’t continuously registered with SAIL throughout the study period. Gaps of less than 30 days during this period were permitted as it could take up to 30 days to transfer records between primary care practices.

Children with no prenatal exposure to antidepressants or depression, but who were exposed prenatally to anxiolytics, antipsychotics, anxiety or severe mental illness (including psychosis, schizophrenia and bipolar disorder) were excluded.

### Covariates

We adjusted for maternal (age, smoking status, parity, multiple births, and comorbid epilepsy) and child (sex, age, ethnicity, and WIMD quintile at birth) confounders. Covariates were selected a priori based on clinical and epidemiological knowledge of factors associated with both prenatal antidepressant/depression exposure and child neurodevelopmental outcomes. A directed acyclic graph was constructed to formalize the assumed causal structure (S1 Appendix). Data quality was optimized using multiple sources. When variables were missing from birth records, we obtained them from primary care or maternity records. Birth weight was truncated at <500 g and >5,500 g, gestational age at <24 weeks and >43 weeks, and maternal age at <11 years; values outside these biologically/clinically plausible limits were converted to missing. Ethnicity was primarily obtained from school census data, with birth records used when missing. Smoking status missing from NCCHD and MIDS was identified from primary care records using validated Read codes [29].

For remaining missing data, our approach was 2-fold. For three non-ordered categorical confounders (maternal smoking status, ethnicity, and mode of delivery) with higher rates of missingness, we used the missing indicator method, where missing values were included as a separate category. This approach was chosen because the data were unlikely to be missing completely at random (for example, mothers who smoke may be less likely to disclose their smoking status). For remaining variables, complete case analysis was used; missingness in these variables was low (<5%) and thus unlikely to introduce substantial bias.

### Outcomes

The primary outcome was all-cause SEN and secondary outcomes were cause-specific SEN and ADHD. SEN is defined as any additional educational support required by a child over and above what one would expect compared to other children of the same age [30]. Cause-specific SEN included: ASD; sensory (visual and hearing) impairment; communication (speech, language and communication) difficulties; learning difficulties (dyslexia, dyspraxia, dyscalculia, and general, specific, moderate, severe, profound and multiple learning difficulties); physical and medical difficulties (PMED); and behavioral-emotional-social (BES) difficulties. Children could have multiple SEN categories recorded. All SEN outcomes were binary. We defined ADHD as either SEN due to ADHD in school records, ADHD medication prescription or an ADHD diagnostic code in primary care records (S1 Appendix). For the ADHD analyses, children were excluded if they did not have complete primary care record coverage from age 5 years until the end of follow-up (gaps less than 30 days were permitted).

### Statistical analyses

Our research aims to answer the question: “Is prenatal exposure to maternal depression and/or antidepressant medication associated with increased risk of special educational need (SEN) identified during childhood (up to age 12)?”. We planned a priori to test for associations between four different exposure groups (record of depression and/or antidepressant medication, antidepressant medications (with/without depression), untreated depression and treated depression) and all-cause SEN and cause-specific SEN, each referent to an unexposed comparison group. We then planned to test specific classes of antidepressants, the most common individual antidepressants within each class, and combinations of medication, for any associations with all-cause SEN and cause-specific SEN, each compared to the unexposed comparison group. In response to reviewers’ comments, the study design was altered to fit a single model for each outcome including binary terms for maternal depression and antidepressant medication exposure, and an interaction term between the two. Absolute risk and risk differences were also estimated. Models were sequentially adjusted for sociodemographic factors (sex, age, ethnicity and WIMD quintile) and further adjusted for maternal factors (maternal age, smoking status, parity, multiple birth and comorbid epilepsy). Unadjusted and partially adjusted estimates are presented in S2–S7 Appendices.

We reported baseline summary statistics for the exposure groups and the comparison group and tested differences using chi-squared tests for categorical variables, chi-squared tests for trend for ordinal variables, and t-tests for continuous variables. For SEN outcomes, generalized estimating equation (GEE) models with a binomial distribution and logit link function were used to account for multiple annual observations per child. An independent correlation structure was specified, selected on the basis of the quasilikelihood under the independence model criterion (QIC) [31], and robust variance estimators were used. The referent group for regression analyses was children of mothers unexposed to both depression and antidepressants. Predicted probabilities of SEN diagnoses were estimated for each combination of maternal depression and antidepressant exposure using the Stata margins command. Average marginal effects (absolute differences in predicted probability) associated with antidepressant exposure were estimated separately for mothers with and without a depression diagnosis. We also examined associations with specific antidepressant classes, combinations of classes and exclusive use of individual medications (all coded as binary variables), where statistical power permitted.

For ADHD, we used Cox proportional hazards models. Models were constructed sequentially: unadjusted; adjusted for sociodemographic factors; and further adjusted for maternal factors. As in the analysis of the other SEN outcomes, models included an interaction term between maternal depression and antidepressant exposure. The proportional hazards assumption was assessed using log-log plots of the survival function and tests based on Schoenfeld residuals. Log-log plots were approximately parallel for the main exposure variables. Follow-up time was measured from date of birth until the date of first ADHD diagnosis or the end of the study period, whichever occurred first. ADHD diagnoses were identified from the earliest record across primary care, prescription data and PLASC data. To estimate absolute measures of risk, flexible parametric survival models (Royston–Parmar models) were additionally fitted using the Stata stpm3 command [32]. The number of degrees of freedom for the baseline spline (6) was selected based on the Akaike and Bayesian information criteria. Marginal cumulative incidence of ADHD diagnoses were estimated for each combination of maternal depression and antidepressant exposure using regression standardization using the Stata standsurv command. Absolute risk differences associated with antidepressant exposure were estimated separately for children of mothers with and without a depression diagnosis, with confidence intervals derived using the delta method. Hazard ratios from the flexible parametric models were consistent with those from the Cox models. Statistical analyses were performed using SPSS Statistics 28.0.1.0 (IBM) and Stata 19.0 SE (StataCorp). Figures were generated in R (version 4.4.0).

### AI tools

Claude Sonnet 4.6 (Anthropic), a large language model, was used to assist with writing Stata code, with all code reviewed and validated by the authors prior to use.

## Results

### Demographic factors

The main cohort comprised 167,477 children, born in Wales between 2009 and 2016, with mean age of 5.68 years (Table 1). Overall, 12,630 children (7.6%) were exposed to untreated maternal depression; 7,005 (4.2%) to treated depression; and 5,996 (3.6%) to antidepressant medication without a record of depression (Table 1). Selective serotonin-reuptake inhibitors (SSRIs) accounted for 83.4% of antidepressant prescribing (S1 Appendix). All-cause SEN was recorded in 47,512 (28.4%) children, including SEN attributed to learning difficulties (15.7%), communication difficulties (11.3%), behavioral-emotional-social difficulties (6.7%), ASD (2.0%), physical and medical difficulties (1.9%) and sensory impairment (1.1%). Overall, 3,050 (1.8%) children had ADHD (Table 1). Children with prenatal depression or antidepressant exposure were more likely to live in deprived areas and have mothers who were younger, smoked during pregnancy, were multiparous, and had comorbid epilepsy. They were less likely to be of Black or Asian ethnicity and had lower gestational age, birth weight centile, and Apgar scores (Table 1).

**Table 1:** Characteristics of cohort by exposure to antidepressant medication and/or depression.

	No depression, no antidepressants^a^	Untreated depression^b^	Treated depression^c^	Antidepressants, no depression^d^	Total
Characteristics, *n* (%)	*n* = 141,816	*n* = 12,630	*n* = 7,005	*n* = 5,996	*n* = 167,447
**Sociodemographic factors**					
Child’s sex					
Female	69,268 (48.8)	6,307 (49.9)	3,403 (48.6)	2,874 (47.9)	81,852 (48.9)
Male	72,548 (51.2)	6,323 (50.1)	3,602 (51.4)	3,122 (52.1)	85,595 (51.1)
Mean age (years (SD))	5.70 (1.11)	5.66 (1.11)	5.48 (1.10)	5.51 (1.1)	5.68 (1.11)
Child’s ethnicity					
White	132,618 (93.5)	12,050 (95.4)	6,732 (96.1)	5,753 (96.0)	157,153 (93.9)
Asian/Asian British	3,151 (2.2)	101 (0.8)	42 (0.6)	50 (0.8)	3,344 (2.0)
Black/Black British	698 (0.5)	33 (0.3)	11 (0.2)	14 (0.2)	756 (0.5)
Mixed/Dual heritage	4,295 (3.0)	392 (3.1)	204 (2.9)	163 (2.7)	5,054 (3.0)
Other	965 (0.7)	43 (0.3)	13 (0.2)	12 (0.2)	1,033 (0.6)
Missing^e^	89 (0.1)	11 (0.1)	3 (0.0)	4 (0.1)	107 (0.1)
Child’s deprivation quintile					
1 (Most Deprived)	35,373 (24.9)	4,348 (34.4)	2,527 (36.1)	1,892 (31.6)	44,140 (26.4)
2	31,046 (21.9)	2,973 (23.6)	1,748 (25.0)	1,470 (24.5)	37,237 (22.2)
3	28,409 (20.0)	2,317 (18.4)	1,206 (17.2)	1,052 (17.6)	32,984 (19.7)
4	22,972 (16.2)	1,686 (13.4)	795 (11.3)	806 (13.4)	26,259 (15.7)
5 (Least Deprived)	23,979 (16.9)	1,298 (10.3)	729 (10.4)	774 (12.9)	26,780 (16.0)
Missing	37	8	0	2	47
**Maternal and obstetric factors**					
Mean maternal age (years (SD))	28.60 (5.89)	26.65 (5.70)	27.95 (5.81)	30.10 (5.8)	28.48 (5.90)
Maternal age (years)					
≤24	37,359 (26.3)	5,166 (40.9)	2,263 (32.3)	1,082 (18.1)	45,870 (27.4)
25–29	41,559 (29.3)	3,709 (29.4)	2,061 (29.4)	1,725 (28.8)	49,054 (29.3)
30–34	38,951 (27.5)	2,394 (19.0)	1,615 (23.1)	1,766 (29.5)	44,726 (26.7)
≥35	23,929 (16.9)	1,361 (10.8)	1,066 (15.2)	1,423 (23.7)	27,779 (16.6)
Missing	18	0	0	0	18
Maternal smoking status					
Non-smoker	61,316 (43.2)	4,369 (34.6)	2,306 (32.9)	2,051 (34.2)	70,042 (41.8)
Quit during pregnancy	4,652 (3.3)	567 (4.5)	305 (4.4)	272 (4.5)	5,796 (3.5)
Smoker	21,049 (14.8)	3,480 (27.6)	2,200 (31.4)	1,620 (27.0)	28,349 (16.9)
Ex Smoker	2,196 (1.5)	255 (2.0)	145 (2.1)	108 (1.8)	2,704 (1.6)
Missing^e^	52,603 (37.1)	3,959 (31.3)	2,049 (29.3)	1,945 (32.4)	60,556 (36.2)
Gestation and sex specific birth weight centiles					
1–3	4,390 (3.1)	485 (3.9)	288 (4.1)	274 (4.6)	5,437 (3.3)
4–10	10,204 (7.2)	1,022 (8.1)	600 (8.6)	481 (8.1)	12,307 (7.4)
11–20	14,265 (10.1)	1,364 (10.9)	780 (11.2)	627 (10.5)	17,036 (10.2)
21–80	84,464 (59.9)	7,415 (59.0)	4,028 (57.9)	3,476 (58.5)	99,383 (59.7)
81–90	13,914 (9.9)	1,126 (9.0)	616 (8.9)	529 (8.9)	16,185 (9.7)
91–97	9,636 (6.8)	790 (6.3)	444 (6.4)	401 (6.7)	11,271 (6.8)
98–100	4,240 (3.0)	364 (2.9)	198 (2.8)	156 (2.6)	4,958 (3.0)
Missing	703	64	51	52	870
Mean gestational age (completed weeks (SD))	39.29 (1.96)	39.06 (2.07)	38.85 (2.16)	38.82 (2.2)	39.24 (1.99)
Gestational age (completed weeks)					
≤27	342 (0.2)	30 (0.2)	21 (0.3)	22 (0.4)	415 (0.2)
28–32	1,481 (1.0)	175 (1.4)	118 (1.7)	105 (1.8)	1,879 (1.1)
33–36	7,475 (5.3)	881 (7.0)	535 (7.7)	470 (7.9)	9,361 (5.6)
37	7,888 (5.6)	838 (6.7)	581 (8.3)	493 (8.3)	9,800 (5.9)
38	16,283 (11.5)	1,652 (13.1)	1,007 (14.5)	875 (14.7)	19,817 (11.9)
39	31,540 (22.3)	2,922 (23.2)	1,704 (24.5)	1,481 (24.9)	37,647 (22.6)
40	39,803 (28.2)	3,359 (26.7)	1,746 (25.1)	1,424 (23.9)	46,332 (27.8)
41	30,022 (21.3)	2,265 (18.0)	1,004 (14.4)	910 (15.3)	34,201 (20.5)
≥42	6,433 (4.6)	467 (3.7)	244 (3.5)	176 (3.0)	7,320 (4.4)
Missing	549	41	45	40	675
Mode of delivery					
Spontaneous Delivery	60,157 (42.4)	5,497 (43.5)	3,108 (44.4)	2,386 (39.8)	71,148 (42.5)
Assisted Delivery	11,330 (8.0)	760 (6.0)	418 (6.0)	390 (6.5)	12,898 (7.7)
Breech	412 (0.3)	31 (0.2)	20 (0.3)	13 (0.2)	476 (0.3)
Elective CS	11,167 (7.9)	1,031 (8.2)	607 (8.7)	566 (9.4)	13,371 (8.0)
Emergency CS	15,193 (10.7)	1,243 (9.8)	705 (10.1)	664 (11.1)	17,805 (10.6)
Missing^e^	43,557 (30.7)	4,068 (32.2)	2,147 (30.6)	1,977 (33.0)	51,749 (30.9)
Parity					
0	61,074 (48.9)	4,700 (42.1)	2,335 (37.3)	2,037 (37.9)	70,146 (47.5)
1	40,519 (32.4)	3,736 (33.5)	1,941 (31.0)	1,624 (30.2)	47,820 (32.4)
>1	23,393 (18.7)	2,715 (24.3)	1,979 (31.6)	1,717 (31.9)	29,804 (20.2)
Missing	16,830	1,479	750	618	19,677
5-min Apgar score					
0–3	242 (0.2)	23 (0.2)	15 (0.2)	11 (0.2)	291 (0.2)
4–6	1,255 (0.9)	114 (0.9)	117 (1.7)	133 (2.3)	1,619 (1.0)
7–10	133,560 (98.9)	11,872 (98.9)	6,564 (98.0)	5580 (97.5)	157,576 (98.8)
Missing	6,759	621	309	272	7,961
Multiple births	4,067 (2.9)	377 (3.0)	201 (2.9)	177 (3.0)	4,822 (2.9)
Comorbid epilepsy	784 (0.6)	114 (0.9)	79 (1.1)	75 (1.3)	1,052 (0.6)
**Outcomes**					
All-cause SEN	38,273 (27.0)	4,437 (35.1)	2,639 (37.7)	2163 (36.1)	47,512 (28.4)
ASD	2,706 (1.9)	319 (2.5)	204 (2.9)	166 (2.8)	3,416 (2.0)
Sensory impairment	1,533 (1.1)	143 (1.1)	83 (1.2)	92 (1.5)	1,851 (1.1)
Communication difficulties	15,228 (10.7)	1,780 (14.1)	1,044 (14.9)	884 (14.7)	18,936 (11.3)
Learning difficulties	21,289 (15.0)	2,435 (19.3)	1,393 (19.9)	1132 (18.9)	26,249 (15.7)
Physical & medical difficulties	2,495 (1.8)	298 (2.4)	181 (2.6)	145 (2.4)	3,119 (1.9)
BES difficulties	8,523 (6.0)	1,329 (10.5)	800 (11.4)	585 (9.8)	11,237 (6.7)
ADHD	2,290 (1.6)	361 (2.9)	233 (3.3)	166 (2.8)	3,050 (1.8)

^a^No record of antidepressant/anxiolytic/antipsychotic medication and no record of depression/anxiety/severe mental illness diagnosis.

^b^Record of depression diagnosis but no record of antidepressant medication.

^c^Record of depression diagnosis and record of antidepressant medication.

^d^Record of antidepressant medication without a record of depression.

^e^Missing values included as additional category. Chi-squared tests for association or trend for categorical variables, or t-tests for continuous variables, were performed comparing each exposure group to the comparison group. All *p*-values ≤0.001 with exception of sex (not significant for all exposure groups), 5-minute Apgar score (*p* = 0.713 for untreated depression), multiple births (not significant for all exposure groups), and sensory impairment (not significant for all exposure groups). SEN categories are not exclusive.

Abbreviations: SEN, special educational need; ADHD, attention deficit hyperactivity disorder; ASD, autism spectrum disorder; BES, behavioral-emotional-social; CS, cesarean section.

### Exposure to depression or antidepressant medications

The interaction between maternal depression and antidepressant medication exposure was significant for all-cause SEN in all models (unadjusted odds ratio (OR) 0.74 (95% CI [0.68,0.81]), S2 Appendix; adjusted OR (aOR) 0.81 (95% CI [0.73,0.88]), Table 2), suggesting that the effects of maternal depression and antidepressant medication exposure on SEN risk are not independent. With the interaction term included in the model, all-cause SEN was associated with both depression (aOR 1.25 (95% CI [1.20,1.31])) and antidepressants (aOR 1.46 (95% CI [1.38,1.56]; Table 2), referent to unexposed children. Looking at individual causes of SEN, depression was associated with all causes excluding sensory impairment, in particular, ASD (aOR 1.42 (95% CI [1.22,1.64])), behavioral-emotional-social difficulties (aOR 1.56 (95% CI [1.45,1.69])) and ADHD (adjusted hazard ratio (aHR) 1.57 (95% CI [1.37,1.80]); Table 2), compared with unexposed children. Similarly, antidepressant medication exposure was associated with all causes of SEN, in particular, ASD (aOR 1.59 (95% CI [1.31,1.92])), behavioral-emotional-social difficulties (aOR 1.70 (95% CI [1.52,1.90])) and ADHD (aHR 1.89 (95% CI [1.57,2.27]); Table 2), referent to unexposed children.

**Table 2:** Association between record of depression or antidepressant medications with special educational need.

	All-cause SEN		ASD		Sensory impairment		Communication difficulties		Learning difficulties		PMED		BESD		ADHD^a^	
	*N* = 623,941		*N* = 623,941		*N* = 623,941		*N* = 623,941		*N* = 623,941		*N* = 623,941		*N* = 623,941		*N* = 132,779	
Exposure	aOR	95% CI	aOR	95% CI	aOR	95% CI	aOR	95% CI	aOR	95% CI	aOR	95% CI	aOR	95% CI	aHR	95% CI
***No interaction***																
Depression	1.19	1.15, 1.24	1.31	1.15, 1.50	0.87	0.71, 1.05	1.10	1.04, 1.17	1.12	1.06, 1.18	1.12	0.97, 1.29	1.45	1.35, 1.56	1.40	1.24,1.60
Antidepressants	1.33	1.27, 1.39	1.37	1.17, 1.59	1.29	1.04, 1.60	1.22	1.14, 1.31	1.24	1.17, 1.32	1.23	1.05, 1.46	1.43	1.31, 1.55	1.50	1.29, 1.74
***Interaction***																
Depression	1.25	1.20, 1.31	1.42	1.22, 1.64	0.89	0.71, 1.11	1.16	1.08, 1.23	1.16	1.09, 1.22	1.18	1.01, 1.37	1.56	1.45, 1.69	1.57	1.37, 1.80
Antidepressants	1.46	1.38, 1.56	1.59	1.31, 1.92	1.34	1.03, 1.75	1.35	1.23, 1.48	1.33	1.23, 1.44	1.36	1.09, 1.68	1.70	1.52, 1.90	1.89	1.57, 2.27
Depression × Antidepressants	0.81	0.73, 0.88	0.72	0.54, 0.97	0.90	0.59, 1.39	0.81	0.71, 0.93	0.86	0.76, 0.96	0.81	0.59, 1.11	0.71	0.61, 0.83	0.62	0.47, 0.82

Odds ratios estimated using generalized estimating equation models, based on annual school census records, with each child contributing one observation per school year. Hazard ratios (ADHD) estimated using Cox analysis, one record per child. A single model was fitted for each outcome of interest, including terms for maternal depression and antidepressant exposure, with and without an interaction term. The referent group was children of mothers with no antidepressant exposure or depression diagnosis. Adjusted for sex, deprivation quintile, ethnicity, age, maternal age, maternal smoking status, parity, multiple birth and maternal epilepsy.

^a^Ethnicity collapsed into 4 categories due to small numbers. × denotes interaction term between maternal depression and antidepressant exposure.

Abbreviations: aOR, adjusted odds ratio; CI, confidence interval; aHR, adjusted hazard ratio; SEN, special educational need; ASD, autism spectrum disorder; PMED, physical and medical difficulties; BESD, behavioral-emotional-social difficulties; ADHD, attention deficit hyperactivity disorder; *N*, total number of observations.

In order to disentangle the contributions of maternal depression and antidepressant exposure to the increased risk of SEN, we estimated the predicted probabilities for each combination (Table 3). For all-cause SEN, depression alone increased the risk from 20 cases per 100 children (95% CI [19.8,20.2]) in those unexposed to depression or antidepressant medication, to 23.6 cases per 100 children (95% CI [22.9,24.3]; Table 3). Antidepressant medication exposure increased the risk similarly whether in combination with depression (26.5 cases per 100 children (95% CI [25.5,27.5])) or without (26.3 cases per 100 children (95% CI [25.3,27.4]); Table 3). Using marginal effects, the difference in predicted probabilities between treated and untreated depression was significant (2.9 additional cases per 100 children (95% CI [1.7,4.1])), although the difference was smaller than that observed among women prescribed antidepressants without any record of diagnosed depression (6.3 additional cases per 100 children (95% CI [5.2,7.4])), due to the higher baseline risk (Table 3).

**Table 3:** Absolute risk of special educational need by record of depression or antidepressants and risk difference.

	Exposure		Absolute risk		Risk difference^a^	
Outcome	Depression	Antidepressants	Predicted probability (cases per 100 children)	95% CI	Marginal effect (additional cases per 100 children)	95% CI
**All-cause SEN**	No	No	20.0	19.8, 20.2		
	No	Yes	26.3	25.3, 27.4	6.3	5.2, 7.4
	Yes	No	23.6	22.9, 24.3		
	Yes	Yes	26.5	25.5, 27.5	2.9	1.7, 4.1
**ASD**	No	No	1.4	1.4, 1.5		
	No	Yes	2.2	1.8, 2.6	0.8	0.4, 1.2
	Yes	No	2.0	1.7, 2.3		
	Yes	Yes	2.3	1.9, 2.7	0.3	−0.2, 0.7
**Sensory impairment**	No	No	0.9	0.8, 0.9		
	No	Yes	1.1	0.9, 1.4	0.3	0.0, 0.6
	Yes	No	0.8	0.6, 0.9		
	Yes	Yes	0.9	0.7, 1.2	0.2	−0.1, 0.5
**Communication difficulties**	No	No	7.4	7.2, 7.5		
	No	Yes	9.6	8.9, 10.3	2.2	1.5, 3.0
	Yes	No	8.4	7.9, 8.8		
	Yes	Yes	9.0	8.4, 9.7	0.7	−0.1, 1.5
**Learning difficulties**	No	No	10.1	10.0, 10.3		
	No	Yes	12.9	12.0, 13.7	2.8	1.9, 3.6
	Yes	No	11.4	10.9, 12.0		
	Yes	Yes	12.8	12.0, 13.5	1.3	0.4, 2.2
**PMED**	No	No	1.3	1.2, 1.4		
	No	Yes	1.8	1.4, 2.1	0.5	0.1, 0.8
	Yes	No	1.5	1.3, 1.7		
	Yes	Yes	1.7	1.4, 2.0	0.1	−0.2, 0.5
**BESD**	No	No	3.9	3.8, 4.0		
	No	Yes	6.4	5.8, 7.0	2.4	1.8, 3.1
	Yes	No	5.9	5.5, 6.3		
	Yes	Yes	7.0	6.4, 7.6	1.1	0.4, 1.8

*N* = 623,941. Depression (record of depression) and antidepressants included in the generalized estimating equation model as binary terms, referent to unexposed. Model fully adjusted for sex, deprivation quintile, ethnicity, age, maternal age, maternal smoking status, parity, multiple birth and comorbid maternal epilepsy. Predicted probabilities represent the estimated probability of a SEN diagnosis for each combination of maternal depression and antidepressant exposure. Marginal effects represent the difference in predicted probabilities, estimated separately for children of mothers with and without a depression diagnosis.

^a^Difference relative to no antidepressants.

Abbreviations: CI, confidence interval; SEN, special educational need; ASD, autism spectrum disorder; PMED, physical and medical difficulties; BESD, behavioral-emotional-social difficulties.

When individual causes of SEN were examined, similar trends were observed whereby antidepressants were associated with a greater increase in risk of SEN among those not exposed to depression compared to those exposed to depression. In particular, learning difficulties showed significant differences in predicted probabilities between treated and untreated depression (1.3 additional cases per 100 children (95% CI [0.4,2.2])), and BES difficulties showed a similar difference (1.1 additional cases per 100 children (95% CI [0.4,1.8]); Table 3). No significant differences were observed for PMED and sensory impairment which could be due to low numbers (Table 3). Flexible parametric modeling was used to estimate cumulative incidence of ADHD and it followed a similar pattern to the other SEN outcomes, with the highest predicted cumulative incidences occurring in the no depression, antidepressant exposed group (3.73 cases per 100 children (95% CI [3.13,4.45]) at age 12) and the treated depression group (3.66 cases per 100 children (95% CI [3.12,4.29]) at age 12; Table 4). Risk difference was also calculated and there was significant difference in risk between those exposed and unexposed to antidepressants in the non-depressed groups at ages 5, 8 and 12 at similar levels to the other SEN outcomes, however, this was attenuated and became non-significant in the depression groups due to the higher baseline risk (Table 4).

**Table 4:** Predicted cumulative incidence of ADHD diagnosis by maternal depression and antidepressant medication exposure and risk difference.

Exposure	Age 5 years		Age 8 years		Age 12 years	
	Predicted cumulative incidence, per 100 children	95% CI	Predicted cumulative incidence, per 100 children	95% CI	Predicted cumulative incidence, per 100 children	95% CI
No depression, no antidepressants	0.07	0.06, 0.09	0.77	0.72, 0.82	2.01	1.89, 2.13
No depression, antidepressants	0.14	0.11, 0.18	1.44	1.20, 1.73	3.73	3.13, 4.45
Depression, no antidepressants	0.12	0.10, 0.14	1.21	1.06, 1.37	3.13	2.75, 3.55
Depression, antidepressants	0.14	0.11, 0.17	1.42	1.20, 1.67	3.66	3.12, 4.29
	**Risk difference, per 100 children^a^**	**95% CI**	**Risk difference, per 100 children^a^**	**95% CI**	**Risk difference, per 100 children^a^**	**95% CI**
Antidepressants effect at Depression = 0	0.07	0.04, 0.09	0.67	0.41, 0.93	1.72	1.06, 2.38
Antidepressants effect at Depression = 1	0.02	−0.01, 0.05	0.21	−0.06, 0.48	0.53	−0.15, 1.22

*N* = 132,779. Predicted cumulative incidence and risk differences estimated using flexible parametric survival models (stpm3) with regression standardization. All models include an interaction term between maternal depression and antidepressant exposure and are adjusted for sex, deprivation quintile, ethnicity, age, maternal age, maternal smoking, parity, multiple birth, and maternal epilepsy.

^a^Risk difference represents the difference in predicted cumulative incidence of ADHD diagnosis between antidepressant-exposed and unexposed children, estimated separately for children of mothers with and without a depression diagnosis.

Abbreviations: ADHD, attention deficit hyperactivity disorder; CI, confidence interval.

### Exposure to different antidepressant classes and individual antidepressants

Further analyses were performed to explore the association between different classes and individual antidepressant medications and SEN risk. Following GEE analysis, adjusted odds ratios were generally similar across the different antidepressant classes and individual antidepressants for all-cause SEN as well as specific causes of SEN (Fig 1A, S5 Appendix). Estimates for less commonly prescribed antidepressants were imprecise, reflected by wide confidence intervals (Fig 1A, S5 Appendix). Similarly, there were no marked differences in adjusted hazard ratios for ADHD between antidepressant classes and individual antidepressants (Fig 1B, S6 Appendix). The adjusted estimates for maternal depression remained consistent across all models for individual antidepressant types in each outcome (S5 and S6 Appendices).

**Fig 1:**
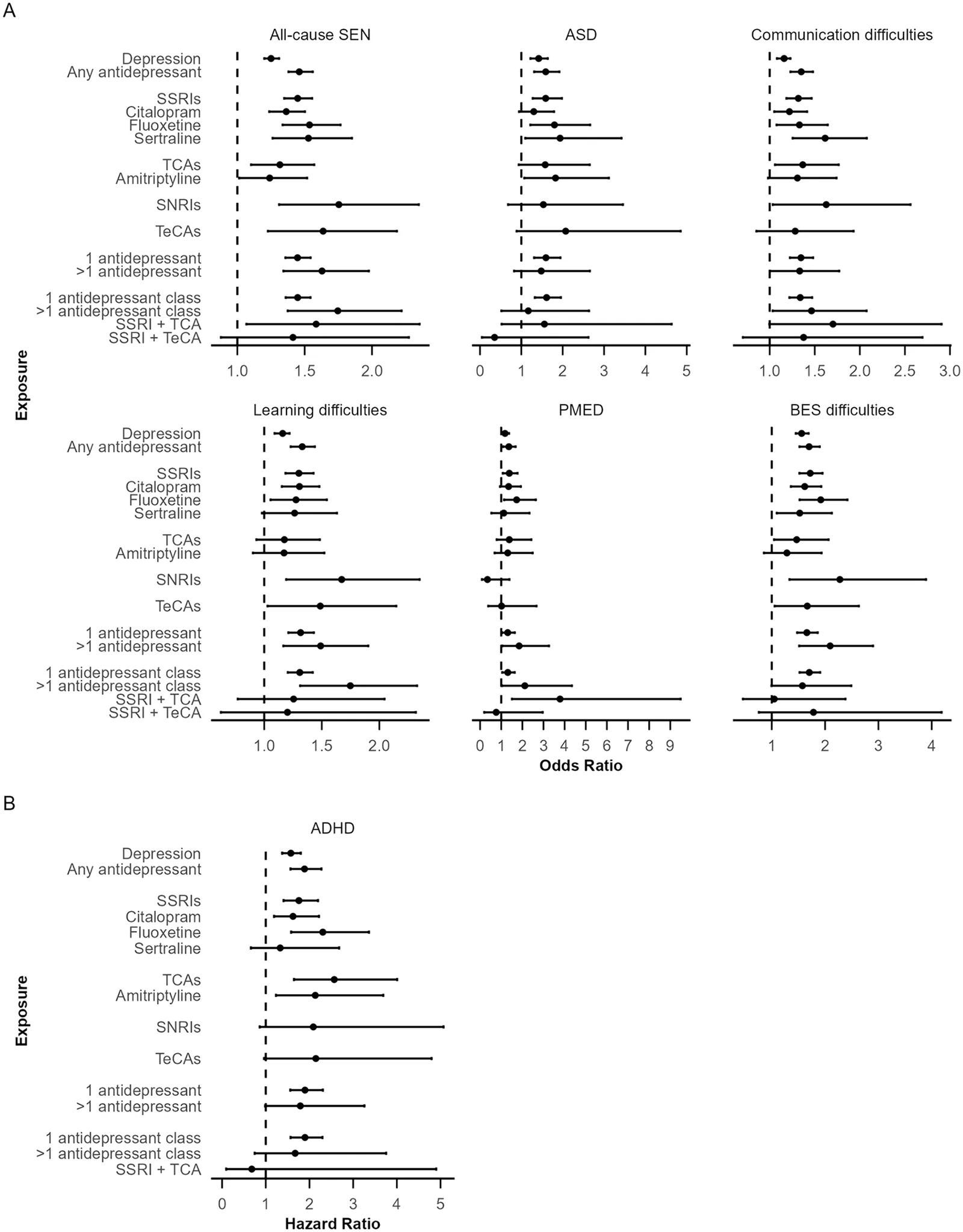
Associations between antidepressant class and individual antidepressant medications and special educational need. **A.** Generalized estimating equation (GEE) analysis; **B.** Cox proportional hazards analysis of associations between antidepressants and ADHD. Odds ratios/hazard ratios and 95% confidence intervals shown, for children exposed to treated depression compared to unexposed. Adjusted for sex, deprivation quintile, ethnicity, age, maternal age, maternal smoking status, parity, multiple birth and maternal epilepsy. For ADHD, ethnicity was collapsed into 4 categories (White; Black/Asian/Mixed; Other; Missing). SEN, special educational need; ASD, autism spectrum disorder; PMED, physical and medical difficulties; BES, behavioral-emotional-social; ADHD, attention deficit hyperactivity disorder; SSRIs, selective serotonin reuptake inhibitors; TCAs, tricyclic antidepressants; TeCAs, tetracyclic antidepressants; SNRIs, serotonin and norepinephrine reuptake inhibitors.

Predicted probability of all-cause SEN was estimated for each antidepressant exposure group. SSRIs, both at class level, as well as individual SSRIs, followed the same pattern as antidepressants as a whole; absolute risk was greatest in those exposed to antidepressants with or without depression (Table 5). However, for TCAs, SNRIs and multi-class combinations, the marginal effect of antidepressants was similar, or greater, in those children exposed to depression compared to those not exposed (Table 5). This was not significant for SSRIs + TeCAs, likely due to very small numbers, reflected by the imprecise estimates (Table 5). These differences could reflect confounding by severity.

**Table 5:** Absolute risk of all-cause special educational need by record of depression or antidepressants and risk difference for individual antidepressants.

	Exposure		Absolute risk		Risk difference^a^	
Antidepressant (N antidepressant exposed/N total observations)	Depression	Antidepressants	Predicted probability (cases per 100 children)	95% CI	Marginal effect (additional cases per 100 children)	95% CI
SSRI only (34,929/614,202)	No	No	20.0	19.8, 20.2		
	No	Yes	26.1	24.9, 27.4	6.1	4.8, 7.4
	Yes	No	23.5	22.8, 24.2		
	Yes	Yes	25.5	24.4, 26.6	2.0	0.7, 3.2
Citalopram only (18,043/597,316)	No	No	19.9	19.7, 20.1		
	No	Yes	25.0	23.3, 26.7	5.0	3.3, 6.7
	Yes	No	23.4	22.7, 24.1		
	Yes	Yes	25.5	24.0, 27.1	2.1	0.4, 3.8
Fluoxetine only (9,027/588,300)	No	No	19.9	19.7, 20.1		
	No	Yes	27.1	24.5, 29.6	7.2	4.6, 9.7
	Yes	No	23.4	22.7, 24.1		
	Yes	Yes	26.0	23.9, 28.1	2.6	0.4, 4.8
Sertraline only (4,441/583,714)	No	No	19.9	19.7, 20.1		
	No	Yes	27.0	23.5, 30.5	7.1	3.6, 10.6
	Yes	No	23.4	22.7, 24.1		
	Yes	Yes	24.5	21.8, 27.3	1.2	−1.7, 4.0
TCA only (3,516/582,789)	No	No	19.9	19.7, 20.1		
	No	Yes	24.3	21.3, 27.4	4.4	1.4, 7.5
	Yes	No	23.3	22.6, 24.1		
	Yes	Yes	32.1	26.6, 37.7	8.8	3.2, 14.4
Amitriptyline only (1,763/581,744)	No	No	19.9	19.7, 20.1		
	No	Yes	23.3	19.9, 26.7	3.4	0.0, 6.8
	Yes	No	23.3	22.6, 24.0		
	Yes	Yes	31.4	23.3, 39.4	8.0	−0.1, 16.1
SNRI only (1,500/580,773)	No	No	19.9	19.7, 20.1		
	No	Yes	29.6	23.9, 35.2	9.7	4.0, 15.3
	Yes	No	23.3	22.6, 24.0		
	Yes	Yes	32.2	26.0, 38.3	8.8	2.6, 15.0
TeCA only (1,815/581,088)	No	No	19.9	19.7, 20.1		
	No	Yes	28.3	22.8, 33.7	8.4	2.9, 13.8
	Yes	No	23.4	22.7, 24.1		
	Yes	Yes	26	21.0, 30.9	2.6	−2.4, 7.6
1 antidepressant only (39,668/618,941)	No	No	20	19.8, 20.2		
	No	Yes	26.1	25.0, 27.3	6.1	5.0, 7.3
	Yes	No	23.6	22.8, 24.3		
	Yes	Yes	26.3	25.2, 27.4	2.7	1.5, 4.0
>1 antidepressant (5,000/584,273)	No	No	19.9	19.7, 20.1		
	No	Yes	28.2	24.5, 31.8	8.3	4.6, 11.9
	Yes	No	23.4	22.7, 24.1		
	Yes	Yes	27.1	24.4, 29.8	3.7	1.0, 6.5
1 class only (41,988/621,261)	No	No	20.0	19.8, 20.2		
	No	Yes	26.1	25.0, 27.3	6.1	5.0, 7.3
	Yes	No	23.6	22.9, 24.3		
	Yes	Yes	26.1	25.1, 27.1	2.5	1.3, 3.8
>1 class (2,680/581,953)	No	No	19.9	19.7, 20.1		
	No	Yes	29.5	24.9, 34.1	9.6	5.0, 14.2
	Yes	No	23.4	22.6, 24.1		
	Yes	Yes	31.3	27.3, 35.4	8.0	3.9, 12.1
SSRI + TCA only (1,048/580,321)	No	No	19.9	19.7, 20.1		
	No	Yes	27.6	20.3, 35.0	7.7	0.4, 15.1
	Yes	No	23.3	22.6, 24.0		
	Yes	Yes	28.4	21.6, 35.1	5.0	−1.7, 11.8
SSRI + TeCA only (582/579,855)	No	No	19.9	19.7, 20.1		
	No	Yes	25.5	17.1, 34.0	5.7	−2.8, 14.1
	Yes	No	23.3	22.6, 24.0		
	Yes	Yes	29.4	22.1, 36.6	6.0	−1.2, 13.3

Predicted probabilities represent the estimated probability of a SEN diagnosis for each combination of maternal depression and antidepressant exposure. Marginal effects represent the difference in predicted probabilities, estimated separately for children of mothers with and without a depression diagnosis.

^a^Difference relative to no antidepressants. Depression (record of depression) and antidepressants included in the generalized estimating equation model as binary terms. An interaction term between maternal depression and antidepressant exposure is included in all models. Model fully adjusted for sex, deprivation quintile, ethnicity, age, maternal age, maternal smoking status, parity, multiple birth and comorbid maternal epilepsy.

Abbreviations: CI, confidence interval; SSRI, selective serotonin reuptake inhibitors; TCA, tricyclic antidepressants; SNRI, serotonin and norepinephrine reuptake inhibitors; TeCA, tetracyclic antidepressants.

## Discussion

In this large population-based cohort of over 167,000 children, we found that prenatal exposure to maternal depression and/or antidepressant medications was associated with increased risk of special educational need in offspring; in particular, ASD, ADHD and behavioral-emotional-social difficulties. These associations remained significant after adjusting for confounders. Using marginal effects to allow direct comparison between treated depression, untreated depression, and antidepressant exposure without depression, we found that antidepressant exposure was associated with an additional 6.3 cases per 100 children (95% CI [5.2,7.4]) in those not exposed to depression, and an additional 2.9 cases per 100 children (95% CI [1.7,4.1]) in those exposed to depression. This reflects a higher baseline risk of all-cause SEN in the depression exposed group, highlighting that the risks associated with maternal depression cannot be attributed solely to antidepressants. This pattern may indicate additional risks from exposure to antidepressants or simply demonstrate that medication use serves as a marker of more severe depression. Antidepressant medication exposure without a record of depression was also associated with increased risk, but it is likely that many of these mothers had unrecorded depression (or anxiety), although TCAs and SNRIs are also prescribed for other indications including migraine, fibromyalgia and neuropathic pain [33,34]. This finding was broadly replicated across all SEN outcomes, with a significant marginal effect of antidepressant medication in those not exposed to depression, but only learning difficulties and behavioral-emotional-social difficulties showed small significant differences in the marginal effect of antidepressants in depression exposed groups. Exposure to TCAs and SNRIs in particular had the biggest differences in risk of SEN between treated and untreated depression.

Our finding that maternal depression itself was associated with SEN concurs with previous studies [10,14,35–38]. Specifically, previous systematic reviews and meta-analyses have found that untreated poor perinatal maternal mental health is associated with adverse neurodevelopment in school-age and adolescent children [8,38,39]. We found differences in risk of all-cause SEN across the exposure groups and showed that antidepressants were associated with a 2.9% increase in absolute risk of SEN in those exposed to treated depression, compared to untreated depression. While we found significant associations between antidepressants and all SEN types studied, marginal effects demonstrated no significant differences in ASD or ADHD risk between untreated depression and treated depression, in line with several previous studies [12,14–17,38,40–45]. However, many studies have also reported an association between antidepressant exposure and ADHD [14,46–50]. One small cohort study reported a higher risk of ADHD in a sub-group of children aged 7–9 years, similar age to the children in our study, which was not fully explained by maternal depression/anxiety [51]. Likewise, many earlier studies examining ASD risk also found an association with antidepressant exposure [10,11,36], and a more recent cohort study using multiple methods found some evidence of an association with ASD in children of women who had initiated antidepressant treatment during pregnancy compared to non-initiators, but not continuers [12]. A recent large meta-analysis found a small association between prenatal antidepressant exposure and ASD and ADHD, but not intellectual disability, motor or speech and language difficulties [52]. The meta-analysis reported a pooled risk ratio (RR) of 1.69 (95% CI [1.24,2.30]) for ASD and 1.35 (95% CI [1.24,1.47]) for ADHD following any antidepressant exposure, which was similar to our estimate for ASD (aOR 1.59, (95% CI [1.31,1.92])), although our estimate for ADHD was higher (aHR 1.89 (95% CI [1.57,2.27])). However, after adjusting for confounding by indication, including restricting to women with mental disorders, sibling analyses and using discontinuers as the referent group, a significant association remained only for TCAs, although they could still not rule out residual confounding by severity [52]. Studies employing paternal antidepressant use as a negative control were included and found to largely be associated with increased risk of ADHD and ASD [13,42,52,53], although not all found an association [36]. Sibling comparison studies to address residual confounding have generally found that any associations between antidepressant exposure and child neurodevelopment are no longer significant [13,36,42,45,50,53–55], although in one study the association remained [56]. However, confounding bias can remain in sibling studies, and environmental and health factors can vary between pregnancies [57,58]. Discontinuer referent groups have also been employed as a means of minimizing bias and have generally shown similar adjusted odds ratios for those who discontinued and those who continued [12,45,52,59]. Together these results suggest that for most antidepressants, although potentially not TCAs, the increased risk is due to genetic or familial factors.

All antidepressant classes studied were associated with greater adjusted odds of all-cause SEN and for most, the marginal effect of antidepressant exposure on all-cause SEN was much smaller in depression-exposed children than non-exposed. However, TCAs, SNRIs and multi-class combinations showed similar or greater risk difference for treated compared to untreated depression. Since SSRIs are generally used as first-line therapy, with TCAs and SNRIs typically reserved for treatment-resistant or more severe depression [60], these differences may represent genuine differences in risk between drugs and drug classes or alternatively, may reflect underlying differences in disease severity. Relatively few studies have examined associations with classes of antidepressants other than SSRIs, or individual antidepressant medications. Whilst some studies have reported no increased risk with TCAs [11,41,43,45,61] or SNRIs [43,45,53], others have reported an association between ASD and TCAs or multi-class combinations [35,47], and TCAs and ADHD [46]. However, in many of these studies the numbers were small, and/or confounding by indication could not be ruled out. As described above, in their meta-analysis, Chan and colleagues found that TCAs were the only antidepressant class for which associations remained significant [52]. To our knowledge, only one sibling analysis has reported estimates specifically for TCAs and it was underpowered [55]. Further research into TCAs and SNRIs is warranted, particularly given that they are also prescribed during pregnancy for other indications.

Our study found an overall association between antidepressant exposure and all-cause SEN, but only learning difficulties and behavioral-emotional-social difficulties showed differences in risk between treated and untreated depression following exposure to antidepressant medication during pregnancy. Few previous studies have examined neurodevelopmental outcomes aside from ASD and ADHD. Heuvelman and colleagues looked at multiple neurodevelopmental disorders but found no associations with antidepressants overall after adjustment for maternal mental health disorders [12,45]. Two studies reported no association between antidepressant exposure and intellectual disability [40,45], however, in one the outcome was very rare (0.5%) [40]. Lupatelli and colleagues found an association between exposure to depression/anxiety treated with SSRIs and increased risk of anxious/depressed behaviors in children at age 5 years [62], also adjusting for maternal mental illness. Bliddal and colleagues also reported an association between SSRI use and emotional disorders in children, although driven at least partly by parental depression [63]. Brandlistuen and colleagues found an association between antidepressant use in pregnancy and internalizing behaviors in children, specifically anxiety [56]. This outcome-specific pattern suggests that the combined effect of treated depression on SEN risk is not uniform across all causes of SEN, although the mechanism is unclear.

Our study was a large, population-wide study across the whole of Wales, providing power to fill existing evidence gaps by comparing a range of medications and medication classes and a range of outcomes that extended beyond ASD and ADHD. The baseline rate of special educational need in our cohort was in line with a recent report on special educational need in Wales [64]. We adjusted for several potential child and maternal confounders. A particular strength of our study was the inclusion of a group exposed to untreated maternal depression. Previous systematic reviews and meta-analyses have highlighted that analysis of untreated depression is required rather than simply adjusting for maternal depression [65–67]. Our unified model with an interaction term between depression and antidepressant medications allowed us to separate out those different exposure groups. Routinely collected data eliminated selection and recall biases and ascertainment from primary care records ensured the inclusion of less severe disease.

We cannot rule out confounding by severity of indication as we lacked data on depression severity. Untreated depression may be less severe. However, one study found that the untreated group had more severe symptoms [68]. Unmeasured confounders and residual confounding may also have influenced our results. We did not have data on alcohol or illicit drug use which could be associated with both maternal depression and subsequent child neurodevelopment. Some children may have been further exposed to medication postnatally via breastmilk, however, breastfeeding status was poorly recorded in the available datasets therefore it was not possible to examine this. However, studies have shown that women with depression, treated and untreated, have lower breastfeeding initiation [6,26]. Conversely, breastfeeding has been associated with reduced risk of postnatal depression [69,70], which could also mitigate the effects on child development. Postnatal or paternal depression could also impact child neurodevelopment, but we did not have access to these data. This could be considered in future study designs to mitigate residual confounding, as well as including other unmeasured confounders, such as data around family history of mental illness and environment. ADHD and ASD can be heritable conditions but we did not have data on maternal neurodevelopmental conditions [71]. Since recent studies in the UK reported prevalences of 0.07% and 0.2% for ADHD and ASD, respectively, in adult women [72,73] any effect is likely to have been small. There is a risk of exposure misclassification due to non-adherence with prescribed medication, however, this would likely result in an underestimation of effect. There is also a small risk of exposure misclassification due to women being prescribed antidepressants in settings other than primary care, such as private practice or during inpatient hospital treatment, or alternatively during the period of transferring between primary care practices, and being classed as non-exposed, but this is likely to be low. A lookback period of 2 years prior to delivery was used to include women who were diagnosed with depression prior to pregnancy, on the assumption that the depression was also present during pregnancy, however, this is a limitation of the study. The Welsh population is predominantly White, therefore our study may not be generalizable to more multi-ethnic populations. This is particularly relevant considering that ethnic minority groups are less likely to present at healthcare providers and therefore could be more likely to be misclassified as non-depressed in our study [74].

In the UK, SSRIs are recommended as the first-line treatment of depression during pregnancy [60]. Of the 7.8% of pregnant women in our cohort who were prescribed antidepressants, 83% were prescribed SSRIs, 10% were prescribed TCAs and 5% were prescribed SNRIs. Prenatal exposure to depression treated with SSRIs was associated with a slightly higher risk of special educational need compared to unexposed, but in terms of absolute risk this was not significantly different to untreated maternal depression. TCAs, SNRIs and multi-class combinations potentially confer a small increase in risk of SEN compared to untreated depression. However, any potential risks of treatment need to be balanced against the risks of untreated maternal depression which was itself associated with higher risk of neurodevelopmental outcomes in offspring and which has been previously shown to be associated with higher risks of postnatal depression [75]. Our findings suggest that there is a small increase in risk of SEN with antidepressant exposure, but we cannot rule out that this is due to more severe depression.

## Supporting information

S1 Checklist STROBE checklist for reporting of observational studies. Completed in accordance with: von Elm E, Altman DG, Egger M, Pocock SJ, Gøtzsche PC, Vandenbroucke JP, et al. (2007) The Strengthening the Reporting of Observational Studies in Epidemiology (STROBE) Statement: Guidelines for Reporting Observational Studies. PLoS Med 4(10):e296. https://doi.org/10.1371/journal.pmed.0040296.
(PDF)

S1 Appendix Supplementary information. (PDF)

S2 Appendix Association between depression and antidepressant exposure and special educational need – unadjusted and partially adjusted models. (PDF)

S3 Appendix Absolute risk of SEN by record of depression or antidepressants and risk difference – unadjusted and partially adjusted models. (PDF)

S4 Appendix Predicted cumulative incidence of ADHD diagnosis by maternal depression and antidepressant medication exposure and risk difference – unadjusted and partially adjusted models. (PDF)

S5 Appendix Association between depression and types of antidepressants and special educational need. (PDF)

S6 Appendix Association between depression and types of antidepressants and ADHD. (PDF)

S7 Appendix Absolute risk of all-cause SEN and types of antidepressants - unadjusted and partially adjusted models. (PDF)

## Abbreviations

ADBE: Annual District Birth Extract
ADHD: attention deficit hyperactivity disorder
aHR: adjusted hazard ratio
ALF: anonymized linking field
aOR: adjusted odds ratio
ASD: autism spectrum disorder
BES: behavioral-emotional-social
BNF: British National Formulary
EDUW: Education Wales
GEE: generalized estimating equation
IGRP: Information Governance Review Panel
MIDS: Maternity Indicators Data Set
NCCHD: National Community Child Health Database
PLASC: Pupil Level Annual School Census
PMED: physical and medical difficulties
QIC: quasilikelihood under the independence model criterion
RR: risk ratio
SAIL: Secure Anonymised Information Linkage
SEN: special educational need
SNRIs: serotonin and norepinephrine reuptake inhibitors
SSRIs: selective serotonin-reuptake inhibitors
STROBE: Strengthening the Reporting of Observational Studies in Epidemiology
TCAs: tricyclic antidepressants
TeCA: tetracyclic antidepressants
WLGP: Welsh Longitudinal General Practice
