# Supplementary material for: Prenatal exposure to maternal depression and antidepressants and neurodevelopmental outcomes: A population cohort study
Source: PLoS Med. 2026 Sep 29;23(9):e1004658. doi: 10.1371/journal.pmed.1004658 (PMC13623103; doi:10.1371/journal.pmed.1004658)
Supplement: Supplementary file 2 [file pmed.1004658.s002.pdf]

Prenatal exposure to maternal depression and antidepressants and neurodevelopmental outcomes: a population cohort study

S1 Appendix. Supplementary information

Saraid McIlvride[1*], Neha Rao[1], Sarjit Singh[2], Scott M Nelson[2], Jill P Pell[1], Michael Fleming[1]

[1]School of Health and Wellbeing, University of Glasgow, Glasgow, UK; [2]School of Medicine, Dentistry and Nursing, University of Glasgow, Glasgow, UK.

S1 Figure. Flow diagram of cohort selection

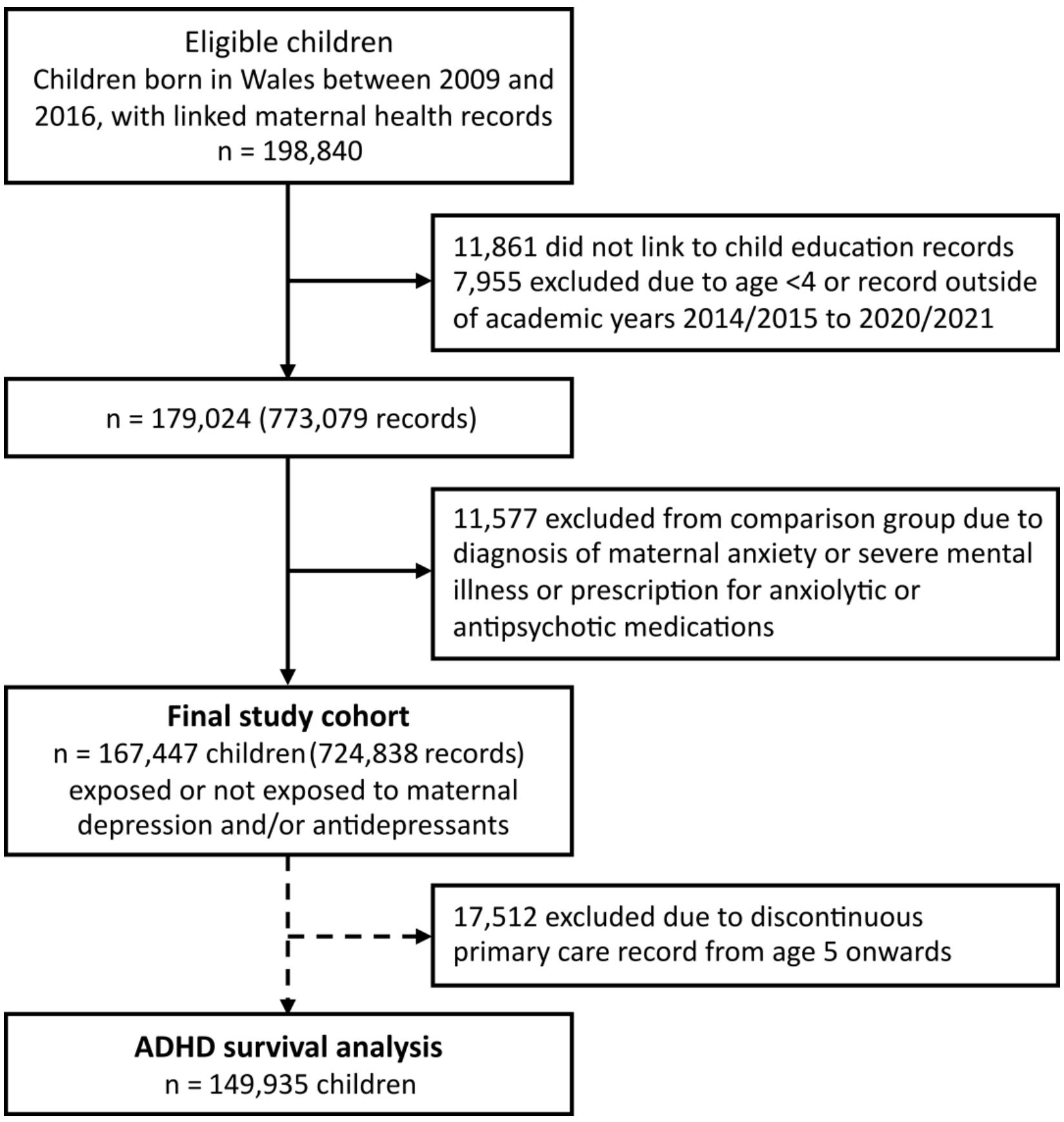

S2 Figure. Simplified Directed Acyclic Graph depicting causal associations between relevant covariates. Minimal sufficient adjustment sets shown.

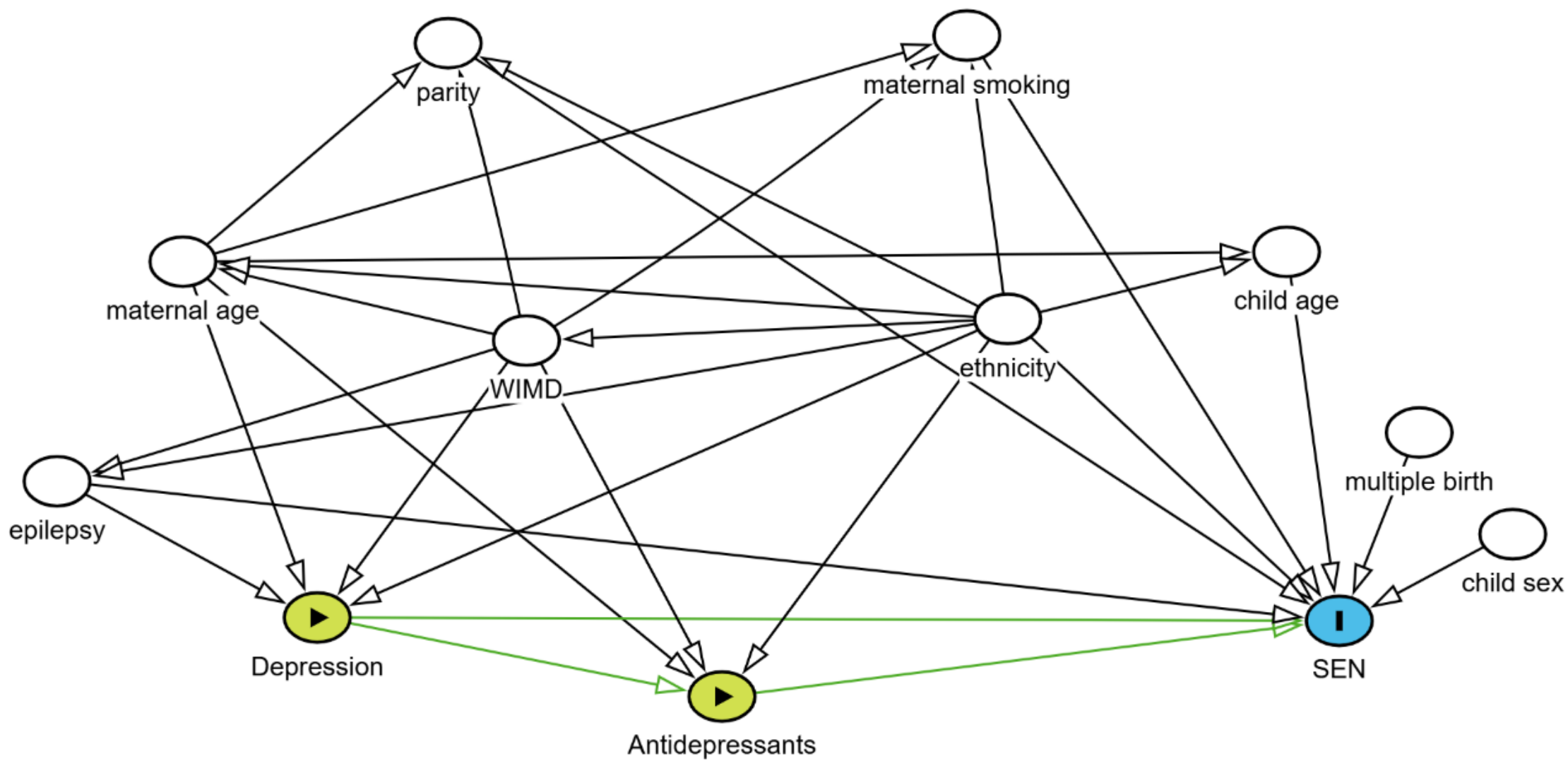

Table A. **Frequencies of psychotropic medications prescribed during pregnancy**

Medication	Total (n=167,447)
Antidepressants	13,001 (7.8%)
SSRIs	10,840 (6.5%)
Citalopram	5,603 (3.3%)
Fluoxetine	3,381 (2.0%)
Sertraline	2,252 (1.3%)
Paroxetine	250 (0.1%)
Escitalopram	123 (0.1%)
TCAs	1,355 (0.8%)
Amitriptyline	1,014 (0.6%)
Lofepramine	104 (0.1%)
Dosulepin	81 (0.0%)
Clomipramine	66 (0.0%)
Imipramine	56 (0.0%)
Nortriptyline	49 (0.0%)
TeCAs (Mirtazapine)	871 (0.5%)
SNRIs	657 (0.4%)
Venlafaxine	416 (0.2%)
Duloxetine	233 (0.1%)
SARIs	110 (0.1%)
1 antidepressant only	11,490 (6.9%)
>1 antidepressant	1,511 (0.9%)
1 antidepressant class only	12,177 (7.27%)
>1 antidepressant class	824 (0.5%)
SSRIs + TCAs only	291 (0.2%)
SSRIs + TeCAs (Mirtazapine) only	204 (0.1%)
Anxiolytics	1,459 (0.9%)
Antipsychotics	1,595 (1.0%)
Antipsychotics excl. prochlorperazine	477 (0.3%)

Categories are not exclusive unless otherwise stated. SSRIs, selective serotonin reuptake inhibitors; TCAs, tricyclic antidepressants; TeCAs, tetracyclic antidepressants; SNRIs, serotonin and norepinephrine reuptake inhibitors; SARIs, serotonin antagonist and reuptake inhibitors.

Table B. Depression and ADHD Read code list (see [https://github.com/smc25/antideps_code.git] for full Read code lists).

Read code (v2)	Description	Sub-category	Category
d9…	Compound antidepressants drugs	Compound	Antidepressants
d91..	Compound antidepressants A-Z	Compound	Antidepressants
d81..	Phenelzine	MAOI	Antidepressants
d82..	Iproniazid	MAOI	Antidepressants
d83..	Isocarboxid	MAOI	Antidepressants
d84..	Tranylcypromine	MAOI	Antidepressants
d85..	Moclobemide	MAOI	Antidepressants
da1..	Flupentixol [antidepressant]	Other	Antidepressants
da2..	Tryptophan	Other	Antidepressants
daD..	Agomelatine	Other	Antidepressants
d7e..	Trazodone hydrochloride	SARI	Antidepressants
da8.	Nefazodone	SARI	Antidepressants
d7g..	Viloxazine	SNRI	Antidepressants
da7..	Venlafaxine	SNRI	Antidepressants
daA..	Reboxetine	SNRI	Antidepressants
gde..	Duloxetine	SNRI	Antidepressants
da3..	Fluvoxamine	SSRI	Antidepressants
da4..	Fluoxetine	SSRI	Antidepressants
da5..	Sertraline	SSRI	Antidepressants
da6..	Paroxetine	SSRI	Antidepressants
da9..	Citalopram	SSRI	Antidepressants
daC..	Escitalopram	SSRI	Antidepressants
daE..	Vortioxetine	SSRI	Antidepressants
d71..	Amitriptyline hydrochloride	TCA	Antidepressants
d72..	Butriptyline	TCA	Antidepressants
d73..	Clomipramine Hydrochloride	TCA	Antidepressants
d74..	Desipramine	TCA	Antidepressants
d75..	Dosulepin hydrochloride	TCA	Antidepressants
d76..	Doxepin	TCA	Antidepressants
d77..	Imipramine hydrochloride	TCA	Antidepressants
d78..	Iprindole	TCA	Antidepressants
d79..	Lofepramine	TCA	Antidepressants
d7c..	Nortriptyline	TCA	Antidepressants
d7d..	Protriptyline	TCA	Antidepressants
d7f..	Trimipramine	TCA	Antidepressants
d7h..	Amoxapine	TCA	Antidepressants
d7a..	Maprotiline hydrochloride	TeCA	Antidepressants
d7b..	Mianserin hydrochloride	TeCA	Antidepressants
daB..	Mirtazapine	TeCA	Antidepressants
gd1..	*amitriptyline hcl [enuresis]	TCA	Antidepressants
gd5..	*imipramine hydrochloride [enuresis]	TCA	Antidepressants
66590	Antidepressant drug treatment started		Depression
1465.	H/O: depression		Depression

1B17.	Depressed		Depression
1B1U	Symptoms of depression		Depression
1BQ..	Loss of capacity for enjoyment		Depression
1BT..	Depressed mood		Depression
1BU..	Loss of hope for the future		Depression
1JJ	Suspected depression		Depression
2257.	O/E - depressed		Depression
62T1.	Puerperal depression		Depression
6G00.	Postnatal depression counselling		Depression
8BK0.	Depression management programme		Depression
8CAa.	Patient given advice about management of depression		Depression
8HHq.	Referral for guided self-help for depression		Depression
9H90.	Depression annual review		Depression
9H91.	Depression medication review		Depression
9H92.	Depression interim review		Depression
9HA0.	On depression register		Depression
9kQ..	On full dose long term treatment depression - enh serv admin		Depression
9Ov..	Depression monitoring administration		Depression
E02y3	Drug-induced depressive state		Depression
E112.	Single major depressive episode		Depression
E113.	Recurrent major depressive episode		Depression
E118.	Seasonal affective disorder		Depression
E11y2	Atypical depressive disorder		Depression
E11z2	Masked depression		Depression
E130.	Reactive depressive psychosis		Depression
E135.	Agitated depression		Depression
E2003	Anxiety with depression		Depression
E204.	Neurotic depression reactive type		Depression
E290.	Brief depressive reaction		Depression
E2900	Grief reaction		Depression
E290z	Brief depressive reaction NOS		Depression
E291.	Prolonged depressive reaction		Depression
E2B..	Depressive disorder NEC		Depression
E2B0.	Postviral depression		Depression
E2B1.	Chronic depression		Depression
Eu204	[X]Post-schizophrenic depression		Depression
Eu251	[X]Schizoaffective disorder, depressive type		Depression
Eu32.	[X]Depressive episode		Depression
Eu33.	[X]Recurrent depressive disorder		Depression
Eu3y1	[X]Other recurrent mood affective disorders		Depression
Eu412	[X]Mixed anxiety and depressive disorder		Depression
Eu530	[X] Mental and behaviour disorders assoc with the puerperium NEC		Depression
Eu920	[X]Depressive conduct disorder		Depression
6A61.	Attention deficit hyperactivity disorder annual review		ADHD diagnosis

8BPT.	Drug therapy for ADHD (attention deficit hyperactivity disorder)		ADHD diagnosis
8BPT0	Stimulant drug therapy for ADHD (attention deficit hyperactivity disorder)		ADHD diagnosis
8BPT1	Non-stimulant drug therapy for ADHD (attention deficit hyperactivity disorder)		ADHD diagnosis
9Ngp.	On drug therapy for ADHD (attention deficit hyperactivity disorder)		ADHD diagnosis
9Ngp0	On stimulant drug therapy for ADHD (attention deficit hyperactivity disorder)		ADHD diagnosis
9Ngp1	On non-stimulant drug therapy for ADHD (attention deficit hyperactivity disorder)		ADHD diagnosis
9Ol8.	Attention deficit hyperactivity disorder monitoring invitation first letter		ADHD diagnosis
9Ol9.	Attention deficit hyperactivity disorder monitoring invitation second letter		ADHD diagnosis
9OlA.	Attention deficit hyperactivity disorder monitoring invitation third letter		ADHD diagnosis
E2E..	Childhood hyperkinetic syndrome		ADHD diagnosis
E2E0.	Child attention deficit disorder		ADHD diagnosis
E2E00	Attention deficit without hyperactivity		ADHD diagnosis
E2E01	Attention deficit with hyperactivity		ADHD diagnosis
E2E0z	Child attention deficit disorder NOS		ADHD diagnosis
E2E1.	Hyperkinesis with developmental delay		ADHD diagnosis
E2E2.	Hyperkinetic conduct disorder		ADHD diagnosis
E2Ey.	Other hyperkinetic manifestation		ADHD diagnosis
E2Ez.	Hyperkinetic syndrome NOS		ADHD diagnosis
Eu90.	[X]Hyperkinetic disorders		ADHD diagnosis
Eu900	[X]Disturbance of activity and attention		ADHD diagnosis
Eu901	[X]Hyperkinetic conduct disorder		ADHD diagnosis
Eu902	Deficits in attention motor control and perception		ADHD diagnosis
Eu90y	[X]Other hyperkinetic disorders		ADHD diagnosis
Eu90z	[X]Hyperkinetic disorder, unspecified		ADHD diagnosis
Eu9y7	Attention deficit disorder		ADHD diagnosis
db...	Weak central nervous system stimulants		ADHD medication
db1..	*Pemoline		ADHD medication
db2..	Weak central nervous system stimulant + vitamins		ADHD medication
dc1..	Dexamfetamine sulfate		ADHD medication
dw...	Drugs used to treat hyperactivity disorders		ADHD medication
dw1..	Methylphenidate		ADHD medication
dw2..	Atomoxetine		ADHD medication
dw3..	Lisdexamfetamine		ADHD medication
dw4..	Guanfacine		ADHD medication
dz...	Other central nervous system stimulants		ADHD medication
dz1..	Modafinil		ADHD medication
