# Supplementary material for: Prenatal exposure to maternal depression and antidepressants and neurodevelopmental outcomes: A population cohort study
Source: PLoS Med. 2026 Sep 29;23(9):e1004658. doi: 10.1371/journal.pmed.1004658 (PMC13623103; doi:10.1371/journal.pmed.1004658)
Supplement: Supplementary file 3 [file pmed.1004658.s003.pdf]

Prenatal exposure to maternal depression and antidepressants and neurodevelopmental outcomes: a population cohort study

Saraid McIlvride[1]*, Neha Rao[1], Sarjit Singh[2], Scott M Nelson[2], Jill P Pell[1], Michael Fleming[1]

[1]School of Health and Wellbeing, University of Glasgow, Glasgow, UK; [2]School of Medicine, Dentistry and Nursing, University of Glasgow, Glasgow, UK.

S2 Appendix

	**All-cause SEN**	**ASD**	**Sensory impairment**	**Communication problems**	**Learning difficulties**	**PMED**	**BESD**	**ADHD[a]**
	**OR (95% CI)**	**OR (95% CI)**	**OR (95% CI)**	**OR (95% CI)**	**OR (95% CI)**	**OR (95% CI)**	**OR (95% CI)**	**HR (95% CI)**
	N = 724,838	N = 724,838	N = 724,838	N = 724,838	N = 724,838	N = 724,838	N = 724,838	N = 149,935
No interaction								
Depression	1.38 (1.33-1.43)	1.27 (1.12-1.44)	0.99 (0.82-1.19)	1.27 (1.20-1.34)	1.28 (1.23-1.34)	1.19 (1.05-1.36)	1.74 (1.63-1.86)	1.67 (1.49-1.87)
Antidepressants	1.39 (1.33-1.45)	1.37 (1.19-1.58)	1.34 (1.10-1.65)	1.32 (1.24-1.41)	1.28 (1.21-1.35)	1.32 (1.13-1.54)	1.43 (1.32-1.54)	1.58 (1.39-1.81)
Interaction								
Depression	1.47 (1.41-1.53)	1.37 (1.20-1.57)	1.08 (0.89-1.32)	1.35 (1.28-1.44)	1.35 (1.28-1.42)	1.26 (1.09-1.45)	1.91 (1.78-2.05)	1.83 (1.62-2.07)
Antidepressants	1.59 (1.50-1.68)	1.59 (1.33-1.90)	1.55 (1.21-1.97)	1.52 (1.40-1.65)	1.42 (1.32-1.53)	1.46 (1.20-1.78)	1.78 (1.61-1.97)	1.91 (1.61-2.26)
Depression × Antidepressants	0.74 (0.68-0.81)	0.72 (0.55-0.95)	0.7 (0.47-1.04)	0.74 (0.65-0.84)	0.8 (0.72-0.89)	0.80 (0.60-1.07)	0.65 (0.56-0.75)	0.69 (0.54-0.88)

Table A. Association between record of depression or antidepressant medications with special educational need – unadjusted model

Odds ratios estimated using generalized estimating equation models, based on annual school census records, with each child contributing one observation per school year. Hazard ratios (ADHD) estimated using Cox analysis, one record per child. A single model was fitted for each outcome of interest, including terms for maternal depression and antidepressant exposure, with and without an interaction term between Depression and Antidepressants. The referent group is children of mothers with no antidepressant exposure or depression diagnosis. [a]Ethnicity collapsed into 4 categories due to small numbers. × denotes the interaction term between maternal depression and antidepressant exposure.

OR, odds ratio; CI confidence interval; HR, hazard ratio; SEN special educational need; ASD autism spectrum disorder; PMED, physical and medical difficulties; BESD, behavioral, emotional, social difficulties; ADHD, attention deficit hyperactivity disorder; N, total number of observations.

Table B. Association between record of depression or antidepressant medications with special educational need – partially adjusted model

	All-cause SEN	ASD	Sensory impairment	Communication problems	Learning difficulties	PMED	BESD	ADHD[a]
	aOR (95% CI)	aOR (95% CI)	aOR (95% CI)	aOR (95% CI)	aOR (95% CI)	aOR (95% CI)	aOR (95% CI)	aHR (95% CI)
	N = 724,595	N = 724,595	N = 724,595	N = 724,595	N = 724,595	N = 724,595	N = 724,595	N = 149,935
No interaction								
Depression	1.32 (1.27-1.37)	1.25 (1.11-1.42)	0.97 (0.81-1.17)	1.21 (1.14-1.28)	1.22 (1.17-1.28)	1.17 (1.03-1.33)	1.65 (1.54-1.76)	1.67 (1.49-1.87)
Antidepressants	1.38 (1.32-1.44)	1.37 (1.19-1.58)	1.34 (1.10-1.65)	1.27 (1.19-1.36)	1.29 (1.22-1.37)	1.31 (1.13-1.53)	1.40 (1.30-1.52)	1.58 (1.39-1.81)
Interaction								
Depression	1.40 (1.34-1.46)	1.35 (1.18-1.55)	1.06 (0.86-1.30)	1.28 (1.21-1.37)	1.28 (1.21-1.34)	1.23 (1.06-1.41)	1.79 (1.67-1.92)	1.83 (1.62-2.07)
Antidepressants	1.55 (1.47-1.65)	1.58 (1.32-1.89)	1.54 (1.21-1.97)	1.43 (1.32-1.56)	1.41 (1.31-1.52)	1.44 (1.18-1.75)	1.70 (1.54-1.89)	1.91 (1.61-2.26)
Depression# Antidepressants	0.77 (0.70-0.83)	0.73 (0.56-0.96)	0.71 (0.47-1.05)	0.76 (0.67-0.87)	0.83 (0.74-0.92)	0.81 (0.61-1.09)	0.68 (0.59-0.79)	0.69 (0.54-0.88)

Odds ratios estimated using generalized estimating equation models, based on annual school census records, with each child contributing one observation per school year. Hazard ratios (ADHD) estimated using Cox analysis, one record per child. A single model was fitted for each outcome of interest, including terms for maternal depression and antidepressant exposure, with and without an interaction term between Depression and Antidepressants. Adjusted for sex, deprivation quintile, ethnicity, and age. The referent group is children of mothers with no antidepressant exposure or depression diagnosis. [a]Ethnicity collapsed into 4 categories due to small numbers.

OR, odds ratio; CI, confidence interval; HR, hazard ratio; SEN, special educational need; ASD, autism spectrum disorder; PMED, physical and medical difficulties; BESD, behavioral-emotional-social difficulties; ADHD, attention deficit hyperactivity disorder; N, total number of observations.
