# Supplementary material for: Prenatal exposure to maternal depression and antidepressants and neurodevelopmental outcomes: A population cohort study
Source: PLoS Med. 2026 Sep 29;23(9):e1004658. doi: 10.1371/journal.pmed.1004658 (PMC13623103; doi:10.1371/journal.pmed.1004658)
Supplement: Supplementary file 4 [file pmed.1004658.s004.pdf]

Prenatal exposure to maternal depression and antidepressants and neurodevelopmental outcomes: a population cohort study

Saraid McIlvride[1]*, Neha Rao[1], Sarjit Singh[2], Scott M Nelson[2], Jill P Pell[1], Michael Fleming[1]

[1]School of Health and Wellbeing, University of Glasgow, Glasgow, UK; [2]School of Medicine, Dentistry and Nursing, University of Glasgow, Glasgow, UK.

S3 Appendix

Table A. Predicted probabilities of SEN diagnosis by antidepressants and maternal depression status - unadjusted model

	Exposure	Predicted probability (cases per 100 children)	95% CI		P value	Marginal effect (additional cases per 100 children)[a]	95% CI		P value
**All-cause SEN**	No depression, no antidepressants	0.20	0.20	0.20	<0.001				
	No depression, antidepressants	0.28	0.27	0.29	<0.001	0.08	0.06	0.09	<0.001
	Depression, no antidepressants	0.27	0.26	0.27	<0.001				
	Depression, antidepressants	0.30	0.29	0.31	<0.001	0.03	0.02	0.04	<0.001
**ASD**	No depression, no antidepressants	0.014	0.014	0.015	<0.001				
	No depression, antidepressants	0.022	0.019	0.026	<0.001	0.008	0.004	0.012	<0.001
	Depression, no antidepressants	0.019	0.017	0.022	<0.001				
	Depression, antidepressants	0.022	0.019	0.026	<0.001	0.003	-0.001	0.007	0.175
**Sensory impairment**	No depression, no antidepressants	0.008	0.008	0.009	<0.001				
	No depression, antidepressants	0.013	0.010	0.016	<0.001	0.004	0.001	0.007	0.004
	Depression, no antidepressants	0.009	0.007	0.011	<0.001				
	Depression, antidepressants	0.010	0.007	0.012	<0.001	0.001	-0.002	0.004	0.598
**Communication difficulties**	No depression, no antidepressants	0.070	0.069	0.071	<0.001				
	No depression, antidepressants	0.103	0.095	0.110	<0.001	0.027	0.020	0.034	<0.001
	Depression, no antidepressants	0.093	0.088	0.098	<0.001				
	Depression, antidepressants	0.103	0.096	0.110	<0.001	0.008	0.000	0.015	0.062
**Learning difficulties**	No depression, no antidepressants	0.100	0.099	0.102	<0.001				
	No depression, antidepressants	0.137	0.128	0.145	<0.001	0.034	0.026	0.042	<0.001
	Depression, no antidepressants	0.131	0.125	0.136	<0.001				
	Depression, antidepressants	0.146	0.138	0.153	<0.001	0.017	0.008	0.026	<0.001
**PMED**	No depression, no antidepressants	0.013	0.013	0.014	<0.001				
	No depression, antidepressants	0.019	0.015	0.023	<0.001	0.006	0.002	0.009	0.002
	Depression, no antidepressants	0.016	0.014	0.019	<0.001				
	Depression, antidepressants	0.019	0.016	0.022	<0.001	0.003	-0.001	0.006	0.168
**BESD**	No depression, no antidepressants	0.038	0.037	0.039	<0.001				
	No depression, antidepressants	0.066	0.060	0.072	<0.001	0.025	0.019	0.030	<0.001
	Depression, no antidepressants	0.071	0.067	0.075	<0.001				
	Depression, antidepressants	0.081	0.075	0.087	<0.001	0.010	0.003	0.016	0.007

N = 724,595. Depression (record of depression) and antidepressants included in the generalized estimating equation (GEE) model as binary terms, referent to unexposed. Unadjusted GEE model with a binomial family and logit link. Predicted probabilities represent the estimated probability of a SEN diagnosis for each combination of maternal depression and antidepressant exposure. Marginal effects represent the difference in predicted probabilities, estimated separately for children of mothers with and without a depression diagnosis. [a]Difference relative to no antidepressants.

CI, confidence interval; SEN, special educational need; ASD, autism spectrum disorder; PMED, physical and medical difficulties; BESD, behavioural-emotional-social difficulties.

Table B. Predicted probabilities of SEN diagnosis by antidepressant type and maternal depression status - partially adjusted model

	Exposure	Predicted probability (cases per 100 children)	95% CI		P value	Marginal effect (additional cases per 100 children)[a]	95% CI		P value
**All-cause SEN**	No depression, no antidepressants	0.20	0.20	0.20	<0.001				
	No depression, antidepressants	0.27	0.26	0.28	<0.001	0.08	0.06	0.09	<0.001
	Depression, no antidepressants	0.25	0.25	0.26	<0.001				
	Depression, antidepressants	0.29	0.28	0.30	<0.001	0.03	0.02	0.04	<0.001
**ASD**	No depression, no antidepressants	0.014	0.014	0.015	<0.001				
	No depression, antidepressants	0.022	0.019	0.026	<0.001	0.008	0.004	0.012	<0.001
	Depression, no antidepressants	0.019	0.017	0.022	<0.001				
	Depression, antidepressants	0.022	0.019	0.025	<0.001	0.003	-0.001	0.007	0.175
**Sensory impairment**	No depression, no antidepressants	0.008	0.008	0.009	<0.001				
	No depression, antidepressants	0.013	0.01	0.016	<0.001	0.004	0.001	0.007	0.004
	Depression, no antidepressants	0.009	0.007	0.011	<0.001				
	Depression, antidepressants	0.010	0.007	0.012	<0.001	0.001	-0.002	0.004	0.598
**Communication difficulties**	No depression, no antidepressants	0.071	0.07	0.072	<0.001				
	No depression, antidepressants	0.098	0.091	0.105	<0.001	0.027	0.020	0.034	<0.001
	Depression, no antidepressants	0.089	0.084	0.093	<0.001				
	Depression, antidepressants	0.096	0.09	0.103	<0.001	0.008	0.000	0.015	0.062
**Learning difficulties**	No depression, no antidepressants	0.101	0.099	0.102	<0.001				
	No depression, antidepressants	0.135	0.127	0.143	<0.001	0.034	0.026	0.042	<0.001
	Depression, no antidepressants	0.124	0.119	0.129	<0.001				
	Depression, antidepressants	0.141	0.134	0.149	<0.001	0.017	0.008	0.026	<0.001
**PMED**	No depression, no antidepressants	0.013	0.013	0.014	<0.001				
	No depression, antidepressants	0.019	0.015	0.022	<0.001	0.006	0.002	0.009	0.002
	Depression, no antidepressants	0.016	0.014	0.018	<0.001				
	Depression, antidepressants	0.019	0.016	0.022	<0.001	0.003	-0.001	0.006	0.168
**BESD**	No depression, no antidepressants	0.039	0.038	0.04	<0.001				
	No depression, antidepressants	0.063	0.058	0.069	<0.001	0.025	0.019	0.03	<0.001
	Depression, no antidepressants	0.066	0.062	0.07	<0.001				
	Depression, antidepressants	0.076	0.07	0.082	<0.001	0.010	0.003	0.016	0.007

N = 724,595. Depression (record of depression) and antidepressants included in the generalized estimating equation model as binary terms, referent to unexposed.
GEE model with a binomial family and logit link, adjusted for sex, age, ethnicity and deprivation index.
Predicted probabilities represent the estimated probability of a SEN diagnosis for each combination of maternal depression and antidepressant exposure.
Marginal effects represent the difference in predicted probabilities, estimated separately for children of mothers with and without a depression diagnosis.
[a]Difference relative to no antidepressants.
CI, confidence interval; SEN, special educational need; ASD, autism spectrum disorder; PMED, physical and medical difficulties; BESD, behavioural-emotional-social difficulties.
