# Supplementary material for: Prenatal exposure to maternal depression and antidepressants and neurodevelopmental outcomes: A population cohort study
Source: PLoS Med. 2026 Sep 29;23(9):e1004658. doi: 10.1371/journal.pmed.1004658 (PMC13623103; doi:10.1371/journal.pmed.1004658)
Supplement: Supplementary file 5 [file pmed.1004658.s005.pdf]

Prenatal exposure to maternal depression and antidepressants and neurodevelopmental outcomes: a population cohort study

Saraid McIlvride[1]*, Neha Rao[1], Sarjit Singh[2], Scott M Nelson[2], Jill P Pell[1], Michael Fleming[1]

[1]School of Health and Wellbeing, University of Glasgow, Glasgow, UK; [2]School of Medicine, Dentistry and Nursing, University of Glasgow, Glasgow, UK.

S4 Appendix. Predicted cumulative incidence of ADHD diagnosis by maternal depression and antidepressant medication exposure and risk difference – unadjusted and partially adjusted models

Table A. Unadjusted model

	Age 5 years			Age 8 years			Age 12 years		
	Predicted cumulative incidence, per 100 children	95% CI		Predicted cumulative incidence, per 100 children	95% CI		Predicted cumulative incidence, per 100 children	95% CI	
No depression, unexposed	0.07	0.06	0.08	0.76	0.72	0.81	2.13	2.03	2.23
No depression, antidepressants	0.14	0.11	0.17	1.45	1.22	1.72	4.02	3.41	4.72
Depression, unexposed	0.13	0.11	0.16	1.39	1.24	1.57	3.86	3.45	4.31
Depression, antidepressants	0.17	0.14	0.21	1.82	1.58	2.10	5.03	4.39	5.77
	Risk difference per 100 children[a]	95% CI		Risk difference per 100 children[a]	95% CI		Risk difference per 100 children[a]	95% CI	
Antidepressants effect at Depression=0	0.07	0.04	0.09	0.69	0.44	0.93	1.89	1.23	2.55
Antidepressants effect at Depression=1	0.04	0.01	0.07	0.43	0.14	0.73	1.18	0.37	1.98

N = 149,935. Predicted cumulative incidence estimated using flexible parametric survival models (stpm3) with regression standardization.
Model includes an interaction term between maternal depression and antidepressant exposure.
Risk difference represents the difference in predicted cumulative incidence between antidepressant-exposed and unexposed children, estimated separately for children of mothers with and without a depression diagnosis.
ADHD, attention deficit hyperactivity disorder; CI, confidence interval.

Table B. Partially adjusted model

	Age 5 years			Age 8 years			Age 12 years		
	Predicted cumulative incidence, per 100 children	95% CI		Predicted cumulative incidence, per 100 children	95% CI		Predicted cumulative incidence, per 100 children	95% CI	
No depression, unexposed	0.07	0.06	0.08	0.76	0.71	0.80	2.03	1.93	2.14
No depression, antidepressants	0.13	0.11	0.16	1.37	1.15	1.62	3.64	3.09	4.29
Depression, unexposed	0.13	0.11	0.15	1.33	1.18	1.50	3.54	3.16	3.97
Depression, antidepressants	0.16	0.14	0.20	1.71	1.48	1.97	4.53	3.95	5.19
	Risk difference per 100 children[a]	95% CI		Risk difference per 100 children[a]	95% CI		Risk difference per 100 children[a]	95% CI	
Antidepressants effect at Depression=0	0.07	0.04	0.09	0.61	0.38	0.84	1.61	1.01	2.20
Antidepressants effect at Depression=1	0.04	0.01	0.07	0.38	0.10	0.66	0.99	0.26	1.71

N = 149,908. Predicted cumulative incidence estimated using flexible parametric survival models (stpm3) with regression standardization.
Model includes an interaction term between maternal depression and antidepressant exposure. Partially adjusted for sex, deprivation, ethnicity, age.
[a]Risk difference represents the difference in predicted cumulative incidence between antidepressant-exposed and unexposed children, estimated separately for children of mothers with and without a depression diagnosis.
ADHD, attention deficit hyperactivity disorder; CI, confidence interval.
