# Supplementary material for: Prenatal exposure to maternal depression and antidepressants and neurodevelopmental outcomes: A population cohort study
Source: PLoS Med. 2026 Sep 29;23(9):e1004658. doi: 10.1371/journal.pmed.1004658 (PMC13623103; doi:10.1371/journal.pmed.1004658)
Supplement: Supplementary file 6 [file pmed.1004658.s006.pdf]

Prenatal exposure to maternal depression and antidepressants and neurodevelopmental outcomes: a population cohort study

Saraid McIlvride[1]*, Neha Rao[1], Sarjit Singh[2], Scott M Nelson[2], Jill P Pell[1], Michael Fleming[1]

[1]School of Health and Wellbeing, University of Glasgow, Glasgow, UK; [2]School of Medicine, Dentistry and Nursing, University of Glasgow, Glasgow, UK.

S5 Appendix

Table A. Association between individual antidepressants and SEN outcomes - unadjusted model

**Antidepressant**		**All-cause SEN**				**ASD**				**Sensory impairment**				**Communication problems**				**Learning difficulties**				**PMED**				**BESD**			
N antidepressant exposed / N total observations		OR	95% CI		P value	OR	95% CI		P value	OR	95% CI		P value	OR	95% CI		P value	OR	95% CI		P value	OR	95% CI		P value	OR	95% CI		P value
SSRI only	Depression	1.47	1.41	1.53	<0.001	1.37	1.20	1.57	<0.001	1.08	0.89	1.32	0.437	1.36	1.28	1.44	<0.001	1.35	1.28	1.42	<0.001	1.26	1.09	1.45	0.002	1.91	1.79	2.05	<0.001
(40,224 / 713,567)	SSRI only	1.57	1.47	1.68	<0.001	1.58	1.28	1.94	<0.001	1.64	1.25	2.16	<0.001	1.49	1.35	1.64	<0.001	1.39	1.28	1.51	<0.001	1.52	1.21	1.91	<0.001	1.80	1.61	2.02	<0.001
	Depression x SSRI interaction	0.71	0.65	0.78	<0.001	0.71	0.52	0.95	0.024	0.70	0.46	1.08	0.105	0.71	0.62	0.82	<0.001	0.80	0.71	0.90	<0.001	0.75	0.54	1.04	0.088	0.59	0.50	0.70	<0.001
Citalopram only	Depression	1.47	1.41	1.53	<0.001	1.37	1.20	1.57	<0.001	1.08	0.89	1.32	0.437	1.36	1.28	1.44	<0.001	1.35	1.28	1.42	<0.001	1.26	1.09	1.45	0.002	1.91	1.79	2.05	<0.001
(20,628 / 693,971)	Citalopram only	1.46	1.33	1.60	<0.001	1.38	1.02	1.86	0.036	1.37	0.92	2.03	0.12	1.34	1.16	1.53	<0.001	1.36	1.21	1.52	<0.001	1.59	1.15	2.18	0.005	1.63	1.39	1.93	<0.001
	Depression x citalo interaction	0.78	0.69	0.88	<0.001	0.80	0.53	1.22	0.3	0.81	0.44	1.47	0.483	0.75	0.62	0.91	0.003	0.87	0.75	1.02	0.091	0.77	0.49	1.19	0.235	0.62	0.50	0.78	<0.001
Fluoxetine only	Depression	1.47	1.41	1.53	<0.001	1.37	1.20	1.57	<0.001	1.08	0.89	1.32	0.437	1.36	1.28	1.44	<0.001	1.35	1.28	1.42	<0.001	1.26	1.09	1.45	0.002	1.91	1.79	2.05	<0.001
(10,597 / 683,940)	fluox only	1.65	1.45	1.88	<0.001	1.72	1.19	2.50	0.004	1.52	0.84	2.73	0.163	1.57	1.29	1.90	<0.001	1.37	1.16	1.62	<0.001	1.69	1.13	2.54	0.011	2.02	1.64	2.50	<0.001
	Depression x fluox interaction	0.72	0.60	0.85	<0.001	0.66	0.39	1.11	0.116	0.98	0.45	2.12	0.957	0.73	0.57	0.95	0.019	0.79	0.63	0.99	0.036	0.60	0.34	1.06	0.078	0.60	0.46	0.80	<0.001
Sertraline only	Depression	1.47	1.41	1.53	<0.001	1.37	1.20	1.57	<0.001	1.08	0.89	1.32	0.437	1.36	1.28	1.44	<0.001	1.35	1.28	1.42	<0.001	1.26	1.09	1.45	0.002	1.91	1.79	2.05	<0.001
(4,997 / 678,340)	sert only	1.67	1.40	1.99	<0.001	1.69	0.96	2.98	0.07	1.43	0.67	3.10	0.358	1.95	1.54	2.46	<0.001	1.29	1.01	1.64	0.040	1.28	0.66	2.47	0.471	1.56	1.14	2.14	0.005
	Depression x sert interaction	0.60	0.48	0.76	<0.001	0.56	0.26	1.19	0.132	0.53	0.18	1.58	0.257	0.60	0.43	0.83	0.002	0.72	0.53	0.99	0.040	0.87	0.38	2.02	0.753	0.65	0.43	0.98	0.037
TCA only	Depression	1.47	1.41	1.53	<0.001	1.37	1.20	1.57	<0.001	1.08	0.89	1.32	0.437	1.36	1.28	1.44	<0.001	1.35	1.28	1.42	<0.001	1.26	1.09	1.45	0.002	1.91	1.79	2.05	<0.001
(3,977 / 677,320)	TCA only	1.38	1.17	1.63	<0.001	1.86	1.15	3.02	0.012	1.79	0.93	3.45	0.084	1.52	1.20	1.92	<0.001	1.15	0.93	1.43	0.203	1.48	0.88	2.47	0.138	1.50	1.10	2.06	0.012
	Depression x TCA interaction	1.20	0.89	1.62	0.233	0.65	0.27	1.57	0.339	0.41	0.12	1.37	0.145	0.96	0.63	1.46	0.848	1.07	0.71	1.62	0.752	1.05	0.38	2.87	0.928	1.19	0.72	1.95	0.498
Amitriptyline only	Depression	1.47	1.41	1.53	<0.001	1.37	1.20	1.57	<0.001	1.08	0.89	1.32	0.437	1.36	1.28	1.44	<0.001	1.35	1.28	1.42	<0.001	1.26	1.09	1.45	0.002	1.91	1.79	2.05	<0.001
(2,776 / 676,119)	amitrip only	1.26	1.05	1.52	0.014	2.15	1.30	3.54	0.003	1.87	0.93	3.78	0.081	1.46	1.13	1.89	0.004	1.10	0.86	1.40	0.438	1.29	0.71	2.33	0.409	1.23	0.85	1.79	0.272
	Depression x amitrip interaction	1.30	0.87	1.96	0.199	0.61	0.17	2.16	0.445	0.35	0.07	1.75	0.199	1.00	0.57	1.75	0.995	0.99	0.54	1.79	0.961	1.30	0.35	4.93	0.696	1.68	0.90	3.12	0.103
SNRI only	Depression	1.47	1.41	1.53	<0.001	1.37	1.20	1.57	<0.001	1.08	0.89	1.32	0.437	1.36	1.28	1.44	<0.001	1.35	1.28	1.42	<0.001	1.26	1.09	1.45	0.002	1.91	1.79	2.05	<0.001
(1,763 / 675106)	SNRI only	1.93	1.49	2.49	<0.001	1.54	0.73	3.24	0.256	0.95	0.25	3.63	0.938	1.75	1.15	2.66	0.009	1.93	1.45	2.57	<0.001	0.30	0.08	1.19	0.087	2.18	1.37	3.49	0.001
	Depression x SNRI interaction	0.81	0.54	1.19	0.280	0.40	0.10	1.61	0.196	1.57	0.23	10.48	0.645	0.92	0.52	1.63	0.774	0.72	0.46	1.12	0.145	3.26	0.53	19.97	0.202	0.59	0.29	1.20	0.143
TeCA only	Depression	1.47	1.41	1.53	<0.001	1.37	1.20	1.57	<0.001	1.08	0.89	1.32	0.437	1.36	1.28	1.44	<0.001	1.35	1.28	1.42	<0.001	1.26	1.09	1.45	0.002	1.91	1.79	2.05	<0.001
(2,105 / 675448)	TeCA only	2.02	1.57	2.59	<0.001	1.81	0.80	4.12	0.157	1.35	0.44	4.13	0.600	1.54	1.05	2.25	0.027	1.87	1.36	2.58	<0.001	1.09	0.46	2.60	0.849	2.14	1.44	3.18	<0.001
	Depression x TeCA interaction	0.64	0.45	0.91	0.012	0.69	0.23	2.01	0.493	0.51	0.11	2.46	0.402	0.85	0.51	1.43	0.548	0.65	0.42	1.02	0.060	1.20	0.36	4.05	0.770	0.73	0.42	1.25	0.248
1 antidepressant only	Depression	1.47	1.41	1.53	<0.001	1.37	1.20	1.57	<0.001	1.08	0.89	1.32	0.437	1.36	1.28	1.44	<0.001	1.35	1.28	1.42	<0.001	1.26	1.09	1.45	0.002	1.91	1.79	2.05	<0.001
(45,764 / 719107)	one_antidep only	1.57	1.48	1.67	<0.001	1.61	1.34	1.94	<0.001	1.53	1.19	1.97	0.001	1.51	1.39	1.65	<0.001	1.40	1.30	1.51	<0.001	1.43	1.16	1.77	0.001	1.74	1.57	1.94	<0.001
	Depression x one_antidep interaction	0.75	0.69	0.82	<0.001	0.68	0.51	0.91	0.008	0.72	0.48	1.09	0.124	0.73	0.64	0.83	<0.001	0.82	0.73	0.92	<0.001	0.84	0.61	1.14	0.254	0.65	0.56	0.76	<0.001
>1 antidepressant	Depression	1.47	1.41	1.53	<0.001	1.37	1.20	1.57	<0.001	1.08	0.89	1.32	0.437	1.36	1.28	1.44	<0.001	1.35	1.28	1.42	<0.001	1.26	1.09	1.45	0.002	1.91	1.79	2.05	<0.001
(5,731 / 679074)	>1 antidep	1.78	1.50	2.12	<0.001	1.42	0.81	2.52	0.226	1.72	0.77	3.84	0.186	1.58	1.21	2.06	0.001	1.59	1.28	1.99	<0.001	1.74	0.99	3.06	0.056	2.18	1.59	2.99	<0.001
	Depression x two_antidep interaction	0.67	0.53	0.83	<0.001	1.06	0.51	2.18	0.875	0.54	0.19	1.55	0.252	0.78	0.56	1.09	0.147	0.65	0.49	0.86	0.003	0.56	0.26	1.22	0.142	0.61	0.42	0.89	0.011
1 class only	Depression	1.47	1.41	1.53	<0.001	1.37	1.20	1.57	<0.001	1.08	0.89	1.32	0.437	1.36	1.28	1.44	<0.001	1.35	1.28	1.42	<0.001	1.26	1.09	1.45	0.002	1.91	1.79	2.05	<0.001
(48,410 / 721753)	1 class only	1.57	1.48	1.66	<0.001	1.62	1.35	1.94	<0.001	1.60	1.25	2.05	<0.001	1.50	1.38	1.64	<0.001	1.40	1.30	1.51	<0.001	1.43	1.17	1.75	0.001	1.79	1.61	1.99	<0.001
	Depression x oneclass interaction	0.74	0.68	0.81	<0.001	0.69	0.52	0.91	0.009	0.70	0.47	1.04	0.077	0.73	0.64	0.83	<0.001	0.81	0.72	0.90	<0.001	0.82	0.61	1.11	0.201	0.63	0.54	0.73	<0.001
>1 class	Depression	1.47	1.41	1.53	<0.001	1.37	1.20	1.57	<0.001	1.08	0.89	1.32	0.437	1.36	1.28	1.44	<0.001	1.35	1.28	1.42	<0.001	1.26	1.09	1.45	0.002	1.91	1.79	2.05	<0.001
(3,085 / 676428)	>1 class only	1.94	1.57	2.40	<0.001	1.15	0.52	2.57	0.732	0.59	0.14	2.54	0.476	1.82	1.32	2.51	<0.001	1.85	1.43	2.40	<0.001	2.02	0.98	4.14	0.056	1.55	0.99	2.42	0.058
	Depression x multiclass interaction	0.74	0.56	0.98	0.037	1.41	0.52	3.83	0.497	1.12	0.17	7.35	0.91	0.80	0.53	1.22	0.300	0.68	0.48	0.97	0.033	0.54	0.19	1.48	0.228	1.08	0.64	1.83	0.775
SSRI + TCA only	Depression	1.47	1.41	1.53	<0.001	1.37	1.20	1.57	<0.001	1.08	0.89	1.32	0.437	1.36	1.28	1.44	<0.001	1.35	1.28	1.42	<0.001	1.26	1.09	1.45	0.002	1.91	1.79	2.05	<0.001
(1,155 / 674498)	ssri_tca only	1.70	1.19	2.43	0.003	1.61	0.56	4.64	0.374	0.99	0.14	7.13	0.995	2.02	1.23	3.33	0.006	1.20	0.75	1.92	0.440	3.76	1.48	9.54	0.005	1.03	0.44	2.38	0.95
	Depression x ssri_tca interaction	0.67	0.41	1.09	0.104	1.26	0.31	5.12	0.747	0.66	0.04	10.80	0.772	0.48	0.24	0.99	0.047	0.87	0.46	1.65	0.666	0.34	0.09	1.33	0.120	1.46	0.55	3.87	0.445
SSRI + TeCA only	Depression	1.47	1.41	1.53	<0.001	1.37	1.20	1.57	<0.001	1.08	0.89	1.32	0.437	1.36	1.28	1.44	<0.001	1.35	1.28	1.42	<0.001	1.26	1.09	1.45	0.002	1.91	1.79	2.05	<0.001
(701 / 674044)	ssri_teca only	1.90	1.19	3.02	0.007	0.31	0.04	2.25	0.247	1.07	0.15	7.78	0.945	2.29	1.16	4.53	0.018	1.66	0.89	3.12	0.113	0.68	0.17	2.72	0.583	1.80	0.83	3.92	0.138
	Depression x ssri_teca interaction	0.80	0.44	1.43	0.448	0.34	0.02	5.57	0.451	1.09	0.07	17.77	0.954	0.98	0.43	2.22	0.961	0.82	0.37	1.80	0.618	1.32	0.17	10.26	0.794	0.80	0.31	2.04	0.635

Odds ratios (OR) estimated using gerneralised estimating equations with a binomial family and logit link. Interaction term included. The referent group is children of mothers with no antidepressant exposure and no depression diagnosis.

SEN, special educational needs; ASD, autism spectrum disorder; PMED, physical and medical difficulties; BESD, behavioral-emotional-social difficulties; OR, odds ratio; CI, confidence interval;

SSRI, selective serotonin reuptake inhibitor; TCA, tricyclic antidepressants; SNRI. Seretonin norpepinephrine reuptake inhibitor; TeCA, tetracyclic antidepressants.

Children exposed to other antidepressant groups were excluded from each analysis.

Table B. Association between individual antidepressants and SEN outcomes - partially adjusted model

Antidepressant		All-cause SEN				ASD				Sensory impairment				Communication problems				Learning difficulties				PMED				BESD			
N antidepressant exposed / N total observations		OR	95% CI		P value	OR	95% CI		P value	OR	95% CI		P value	OR	95% CI		P value	OR	95% CI		P value	OR	95% CI		P value	OR	95% CI		P value
SSRI only	Depression	1.40	1.34	1.46	<0.001	1.35	1.18	1.55	<0.001	1.06	0.87	1.30	0.584	1.28	1.21	1.36	<0.001	1.28	1.21	1.35	<0.001	1.23	1.06	1.41	0.005	1.79	1.67	1.92	<0.001
(40,219 / 713,327)	SSRI only	1.55	1.45	1.66	<0.001	1.58	1.28	1.95	<0.001	1.64	1.24	2.15	<0.001	1.43	1.29	1.57	<0.001	1.39	1.27	1.51	<0.001	1.51	1.20	1.90	<0.001	1.75	1.55	1.96	<0.001
	Depression x SSRI interaction	0.73	0.66	0.80	<0.001	0.71	0.52	0.96	0.026	0.71	0.46	1.09	0.115	0.73	0.63	0.84	<0.001	0.82	0.73	0.93	0.001	0.76	0.55	1.06	0.103	0.62	0.52	0.73	<0.001
Citalopram only	Depression	1.40	1.34	1.46	<0.001	1.35	1.18	1.55	<0.001	1.06	0.87	1.30	0.58	1.28	1.21	1.36	<0.001	1.28	1.21	1.34	<0.001	1.23	1.07	1.42	0.005	1.79	1.67	1.92	<0.001
(20,628 / 693,736)	Citalopram only	1.44	1.32	1.58	<0.001	1.39	1.03	1.88	0.03	1.36	0.92	2.02	0.129	1.29	1.12	1.48	<0.001	1.35	1.20	1.51	<0.001	1.58	1.15	2.17	0.005	1.59	1.34	1.88	<0.001
	Depression x citalo interaction	0.80	0.71	0.91	0.001	0.81	0.53	1.23	0.319	0.81	0.44	1.48	0.498	0.77	0.64	0.94	0.009	0.90	0.77	1.06	0.202	0.78	0.50	1.20	0.258	0.65	0.52	0.82	<0.001
Fluoxetine only	Depression	1.40	1.34	1.46	<0.001	1.35	1.18	1.55	<0.001	1.06	0.86	1.29	0.587	1.28	1.21	1.36	<0.001	1.28	1.21	1.34	<0.001	1.23	1.07	1.42	0.004	1.79	1.67	1.92	<0.001
(10,597 / 683,705	fluox only	1.61	1.41	1.83	<0.001	1.68	1.16	2.44	0.006	1.51	0.84	2.71	0.172	1.48	1.22	1.81	<0.001	1.35	1.14	1.60	0.001	1.67	1.11	2.51	0.013	1.92	1.55	2.38	<0.001
	Depression x fluox interaction	0.73	0.61	0.87	<0.001	0.66	0.39	1.11	0.119	0.98	0.45	2.13	0.967	0.76	0.58	0.98	0.034	0.81	0.64	1.02	0.067	0.60	0.34	1.07	0.083	0.63	0.47	0.83	0.001
Sertraline only	Depression	1.40	1.34	1.46	<0.001	1.35	1.18	1.55	<0.001	1.06	0.86	1.29	0.588	1.28	1.21	1.36	<0.001	1.28	1.21	1.34	<0.001	1.23	1.07	1.42	0.004	1.79	1.67	1.92	<0.001
(4,997 / 678,105)	sert only	1.69	1.41	2.02	<0.001	1.75	1.00	3.08	0.051	1.49	0.69	3.22	0.307	1.76	1.38	2.24	<0.001	1.39	1.09	1.77	0.007	1.29	0.67	2.50	0.45	1.54	1.12	2.12	0.007
	Depression x sert interaction	0.62	0.49	0.78	<0.001	0.56	0.26	1.20	0.134	0.55	0.18	1.62	0.276	0.61	0.44	0.85	0.003	0.76	0.55	1.04	0.081	0.89	0.39	2.06	0.785	0.69	0.45	1.04	0.073
TCA only	Depression	1.40	1.34	1.46	<0.001	1.35	1.18	1.55	<0.001	1.06	0.87	1.30	0.582	1.28	1.21	1.36	<0.001	1.28	1.21	1.34	<0.001	1.23	1.07	1.42	0.004	1.79	1.66	1.92	<0.001
(3,974 / 677,082)	TCA only	1.33	1.13	1.57	0.001	1.82	1.12	2.95	0.015	1.79	0.93	3.47	0.083	1.41	1.11	1.78	0.005	1.14	0.91	1.42	0.25	1.45	0.87	2.44	0.155	1.42	1.04	1.94	0.029
	Depression x TCA interaction	1.17	0.86	1.60	0.314	0.59	0.24	1.44	0.245	0.39	0.12	1.32	0.131	0.97	0.63	1.49	0.873	1.01	0.66	1.55	0.966	1.02	0.37	2.80	0.966	1.13	0.69	1.86	0.62
Amitriptyline only	Depression	1.40	1.34	1.46	<0.001	1.35	1.18	1.55	<0.001	1.06	0.86	1.30	0.586	1.28	1.21	1.36	<0.001	1.28	1.21	1.34	<0.001	1.23	1.07	1.42	0.004	1.79	1.66	1.92	<0.001
(2,776 / 675,884)	amitrip only	1.24	1.03	1.50	0.024	2.18	1.32	3.61	0.002	1.90	0.94	3.84	0.073	1.36	1.05	1.77	0.021	1.13	0.88	1.44	0.351	1.28	0.71	2.33	0.417	1.21	0.83	1.75	0.326
	Depression x amitrip interaction	1.32	0.87	2.00	0.186	0.56	0.16	2.00	0.368	0.34	0.07	1.71	0.19	1.03	0.58	1.84	0.917	0.95	0.52	1.74	0.866	1.28	0.34	4.83	0.715	1.66	0.90	3.08	0.107
SNRI only	Depression	1.40	1.34	1.46	<0.001	1.35	1.18	1.55	<0.001	1.06	0.87	1.30	0.581	1.28	1.21	1.36	<0.001	1.28	1.21	1.34	<0.001	1.23	1.07	1.42	0.004	1.79	1.67	1.92	<0.001
(1,763 / 674,871)	SNRI only	1.84	1.40	2.40	<0.001	1.46	0.69	3.13	0.324	0.93	0.24	3.55	0.911	1.65	1.07	2.53	0.022	1.85	1.37	2.50	<0.001	0.29	0.07	1.16	0.081	2.01	1.23	3.30	0.005
	Depression x SNRI interaction	0.86	0.58	1.28	0.465	0.41	0.10	1.67	0.215	1.60	0.24	10.70	0.63	0.98	0.55	1.76	0.954	0.76	0.48	1.20	0.237	3.36	0.55	20.70	0.192	0.64	0.31	1.32	0.226
TeCA only	Depression	1.40	1.34	1.46	<0.001	1.35	1.18	1.55	<0.001	1.06	0.86	1.30	0.586	1.28	1.21	1.36	<0.001	1.28	1.21	1.34	<0.001	1.23	1.07	1.42	0.004	1.79	1.67	1.92	<0.001
(2,105 / 675,213)	TeCA only	1.83	1.41	2.39	<0.001	1.75	0.76	4.08	0.192	1.32	0.43	4.05	0.632	1.31	0.89	1.93	0.166	1.77	1.27	2.46	0.001	1.05	0.44	2.50	0.92	1.88	1.24	2.84	0.003
	Depression x TeCA interaction	0.69	0.48	1.00	0.049	0.72	0.24	2.16	0.558	0.54	0.11	2.59	0.437	0.91	0.53	1.55	0.729	0.72	0.46	1.13	0.154	1.25	0.37	4.24	0.715	0.82	0.47	1.43	0.479
1 antidepressant only	Depression	1.40	1.34	1.46	<0.001	1.35	1.18	1.55	<0.001	1.06	0.87	1.30	0.572	1.28	1.21	1.37	<0.001	1.28	1.21	1.35	<0.001	1.23	1.06	1.41	0.005	1.79	1.67	1.92	<0.001
(45,761 / 718,869)	one_antidep only	1.54	1.45	1.63	<0.001	1.60	1.33	1.93	<0.001	1.53	1.18	1.97	0.001	1.43	1.31	1.57	<0.001	1.39	1.29	1.50	<0.001	1.42	1.15	1.74	0.001	1.67	1.49	1.86	<0.001
	Depression x one_antidep interaction	0.77	0.71	0.84	<0.001	0.69	0.52	0.91	0.01	0.73	0.48	1.11	0.137	0.75	0.66	0.86	<0.001	0.85	0.76	0.95	0.004	0.85	0.62	1.16	0.296	0.68	0.58	0.80	<0.001
>1 antidepressant	Depression	1.40	1.34	1.46	<0.001	1.35	1.18	1.55	<0.001	1.06	0.86	1.29	0.596	1.28	1.21	1.36	<0.001	1.28	1.21	1.34	<0.001	1.23	1.07	1.42	0.005	1.79	1.67	1.92	<0.001
(5,726 / 678,834)	>1 antidep	1.74	1.45	2.08	<0.001	1.38	0.77	2.47	0.274	1.73	0.77	3.85	0.184	1.44	1.09	1.89	0.009	1.62	1.29	2.03	<0.001	1.72	0.98	3.04	0.06	2.10	1.52	2.90	<0.001
	Depression x two_antidep interaction	0.69	0.55	0.87	0.002	1.12	0.54	2.32	0.768	0.55	0.19	1.56	0.26	0.83	0.59	1.18	0.301	0.66	0.50	0.89	0.006	0.57	0.26	1.23	0.152	0.65	0.44	0.96	0.029
1 class only	Depression	1.40	1.34	1.46	<0.001	1.35	1.18	1.55	<0.001	1.06	0.87	1.30	0.576	1.28	1.21	1.37	<0.001	1.28	1.21	1.35	<0.001	1.23	1.06	1.41	0.005	1.79	1.67	1.92	<0.001
(48,402 / 721,510)	1 class only	1.54	1.45	1.63	<0.001	1.61	1.34	1.93	<0.001	1.59	1.25	2.04	<0.001	1.43	1.31	1.56	<0.001	1.39	1.29	1.50	<0.001	1.41	1.15	1.73	0.001	1.72	1.55	1.91	<0.001
	Depression x oneclass interaction	0.76	0.70	0.83	<0.001	0.69	0.53	0.92	0.011	0.70	0.47	1.05	0.086	0.75	0.66	0.86	<0.001	0.83	0.74	0.93	0.001	0.83	0.62	1.13	0.237	0.65	0.56	0.76	<0.001
>1 class	Depression	1.40	1.34	1.46	<0.001	1.35	1.18	1.55	<0.001	1.06	0.86	1.29	0.59	1.28	1.21	1.36	<0.001	1.28	1.21	1.34	<0.001	1.23	1.07	1.42	0.004	1.79	1.67	1.92	<0.001
(3,085 / 676,193)	>1 class only	1.85	1.48	2.30	<0.001	1.09	0.49	2.45	0.835	0.59	0.14	2.54	0.475	1.59	1.15	2.22	0.006	1.87	1.43	2.44	<0.001	1.98	0.96	4.08	0.063	1.42	0.91	2.22	0.126
	Depression x multiclass interaction	0.82	0.61	1.10	0.177	1.60	0.58	4.38	0.365	1.14	0.17	7.56	0.89	0.91	0.60	1.41	0.682	0.71	0.49	1.03	0.068	0.55	0.20	1.53	0.254	1.26	0.74	2.14	0.394
SSRI + TCA only	Depression	1.40	1.34	1.46	<0.001	1.35	1.18	1.55	<0.001	1.06	0.86	1.29	0.589	1.28	1.21	1.36	<0.001	1.28	1.21	1.34	<0.001	1.23	1.07	1.42	0.004	1.79	1.67	1.92	<0.001
(1,155 / 674,263)	ssri_tca only	1.67	1.15	2.43	0.007	1.54	0.53	4.54	0.43	1.01	0.14	7.25	0.995	1.82	1.09	3.03	0.022	1.25	0.77	2.02	0.362	3.76	1.48	9.55	0.005	0.99	0.44	2.26	0.987
	Depression x ssri_tca interaction	0.73	0.44	1.21	0.219	1.43	0.35	5.93	0.618	0.67	0.04	11.03	0.781	0.56	0.27	1.17	0.126	0.89	0.46	1.72	0.733	0.35	0.09	1.35	0.127	1.69	0.65	4.43	0.286
SSRI + TeCA only	Depression	1.40	1.34	1.46	<0.001	1.35	1.18	1.55	<0.001	1.06	0.86	1.30	0.587	1.28	1.21	1.36	<0.001	1.28	1.21	1.34	<0.001	1.23	1.07	1.42	0.004	1.79	1.67	1.92	<0.001
(701 / 673,809)	ssri_teca only	1.79	1.15	2.80	0.01	0.30	0.04	2.20	0.234	1.05	0.14	7.72	0.959	2.04	1.03	4.07	0.042	1.63	0.88	3.03	0.122	0.67	0.16	2.70	0.569	1.63	0.72	3.67	0.239
	Depression x ssri_teca interaction	0.81	0.45	1.45	0.479	0.35	0.02	5.73	0.459	1.12	0.07	18.47	0.937	0.99	0.43	2.26	0.978	0.85	0.39	1.84	0.672	1.33	0.17	10.41	0.787	0.83	0.31	2.25	0.72

Odds ratios (OR) estimated using gerneralised estimating equations with a binomial family and logit link. Interaction term included. The referent group is children of mothers with no antidepressant exposure and no depression diagnosis.

Partially adjusted models shown - adjusted for sex, deprivation quintile, ethnicity, age.

SEN, special educational needs; ASD, autism spectrum disorder; PMED, physical and medical difficulties; BESD, behavioral-emotional-social difficulties; OR, odds ratio; CI, confidence interval;

SSRI, selective serotonin reuptake inhibitor; TCA, tricyclic antidepressants; SNRI. Seretonin norpepinephrine reuptake inhibitor; TeCA, tetracyclic antidepressants.

Children exposed to other antidepressant groups were excluded from each analysis.

Table C. Association between individual antidepressants and SEN outcomes - fully adjusted model

Antidepressant		All-cause SEN				ASD				Sensory impairment				Communication problems				Learning difficulties				PMED				BESD			
N antidepressant exposed / N total observations		OR	95% CI		P value	OR	95% CI		P value	OR	95% CI		P value	OR	95% CI		P value	OR	95% CI		P value	OR	95% CI		P value	OR	95% CI		P value
SSRI only	Depression	1.25	1.19	1.30	<0.001	1.41	1.22	1.64	<0.001	0.89	0.71	1.12	0.311	1.15	1.08	1.23	<0.001	1.16	1.09	1.22	<0.001	1.17	1.00	1.37	0.044	1.56	1.44	1.69	<0.001
(34,929 / 614,202)	SSRI only	1.45	1.35	1.56	<0.001	1.59	1.28	1.98	<0.001	1.45	1.07	1.95	0.015	1.32	1.19	1.47	<0.001	1.30	1.19	1.43	<0.001	1.38	1.08	1.77	0.011	1.72	1.52	1.95	<0.001
	Depression x SSRI interaction	0.77	0.70	0.86	<0.001	0.71	0.52	0.98	0.038	0.89	0.56	1.42	0.625	0.78	0.67	0.90	0.001	0.86	0.75	0.98	0.021	0.78	0.55	1.11	0.169	0.65	0.55	0.78	<0.001
Citalopram only	Depression	1.25	1.19	1.30	<0.001	1.41	1.22	1.63	<0.001	0.89	0.71	1.12	0.329	1.15	1.08	1.23	<0.001	1.15	1.09	1.22	<0.001	1.17	1.00	1.37	0.045	1.55	1.44	1.68	<0.001
(18,043 / 597,316)	Citalopram only	1.36	1.24	1.50	<0.001	1.30	0.95	1.79	0.107	1.18	0.78	1.79	0.443	1.22	1.05	1.42	0.009	1.31	1.15	1.48	<0.001	1.35	0.94	1.93	0.1	1.62	1.36	1.93	<0.001
	Depression x citalo interaction	0.83	0.72	0.95	0.007	0.86	0.55	1.34	0.503	0.99	0.52	1.88	0.978	0.82	0.67	1.01	0.065	0.91	0.77	1.08	0.291	0.73	0.44	1.18	0.199	0.65	0.51	0.83	0.001
Fluoxetine only	Depression	1.24	1.19	1.30	<0.001	1.41	1.22	1.63	<0.001	0.89	0.71	1.12	0.314	1.15	1.08	1.23	<0.001	1.15	1.09	1.22	<0.001	1.17	1.00	1.37	0.047	1.56	1.44	1.68	<0.001
(9,027 / 588,300)	fluox only	1.54	1.34	1.77	<0.001	1.80	1.22	2.66	0.003	1.29	0.66	2.52	0.461	1.33	1.08	1.64	0.008	1.28	1.06	1.54	0.012	1.73	1.13	2.64	0.011	1.92	1.52	2.42	<0.001
	Depression x fluox interaction	0.76	0.63	0.91	0.004	0.64	0.37	1.11	0.113	1.28	0.53	3.07	0.578	0.79	0.59	1.04	0.09	0.82	0.63	1.05	0.112	0.65	0.35	1.19	0.158	0.67	0.49	0.91	0.01
Sertraline only	Depression	1.24	1.19	1.30	<0.001	1.41	1.22	1.63	<0.001	0.89	0.71	1.12	0.33	1.15	1.08	1.23	<0.001	1.15	1.09	1.22	<0.001	1.17	1.00	1.37	0.044	1.55	1.44	1.68	<0.001
(4,441 / 583,714)	sert only	1.53	1.26	1.85	<0.001	1.94	1.10	3.42	0.023	1.61	0.75	3.49	0.224	1.62	1.26	2.08	<0.001	1.26	0.98	1.63	0.072	1.12	0.54	2.34	0.762	1.53	1.09	2.13	0.013
	Depression x sert interaction	0.70	0.55	0.90	0.006	0.52	0.24	1.13	0.100	0.64	0.21	1.92	0.425	0.67	0.47	0.94	0.022	0.85	0.61	1.19	0.348	1.10	0.44	2.74	0.838	0.76	0.49	1.18	0.217
TCA only	Depression	1.24	1.19	1.30	<0.001	1.41	1.22	1.63	<0.001	0.89	0.71	1.12	0.322	1.15	1.08	1.23	<0.001	1.15	1.09	1.22	<0.001	1.17	1.00	1.37	0.045	1.55	1.43	1.67	<0.001
(3,516 / 582,789)	TCA only	1.32	1.10	1.57	0.002	1.58	0.94	2.65	0.086	1.43	0.67	3.05	0.35	1.37	1.06	1.77	0.016	1.18	0.93	1.48	0.173	1.38	0.79	2.42	0.262	1.47	1.04	2.07	0.028
	Depression x TCA interaction	1.22	0.88	1.70	0.235	0.68	0.26	1.76	0.422	0.45	0.11	1.84	0.267	1.08	0.68	1.69	0.755	0.91	0.57	1.46	0.707	1.25	0.45	3.53	0.67	1.14	0.67	1.93	0.641
Amitriptyline only	Depression	1.24	1.19	1.30	<0.001	1.41	1.22	1.63	<0.001	0.89	0.71	1.12	0.323	1.15	1.08	1.23	<0.001	1.15	1.09	1.22	<0.001	1.17	1.00	1.37	0.045	1.55	1.43	1.67	<0.001
(1,763 / 581,744)	amitrip only	1.24	1.01	1.52	0.037	1.83	1.07	3.12	0.027	1.77	0.81	3.86	0.151	1.31	0.98	1.74	0.066	1.17	0.90	1.52	0.235	1.31	0.69	2.49	0.413	1.28	0.85	1.93	0.231
	Depression x amitrip interaction	1.25	0.79	1.96	0.34	0.65	0.16	2.55	0.532	0.46	0.09	2.42	0.361	1.09	0.59	2.00	0.79	0.78	0.39	1.58	0.492	1.44	0.37	5.61	0.602	1.30	0.65	2.57	0.461
SNRI only	Depression	1.24	1.19	1.30	<0.001	1.41	1.22	1.63	<0.001	0.89	0.71	1.12	0.325	1.15	1.08	1.23	<0.001	1.15	1.09	1.22	<0.001	1.17	1.00	1.37	0.044	1.55	1.43	1.67	<0.001
(1,500 / 580,773)	SNRI only	1.75	1.31	2.35	<0.001	1.53	0.68	3.46	0.303	0.81	0.15	4.32	0.808	1.63	1.03	2.56	0.036	1.67	1.19	2.35	0.003	0.35	0.09	1.39	0.134	2.28	1.33	3.89	0.003
	Depression x SNRI interaction	0.92	0.60	1.40	0.694	0.24	0.05	1.23	0.086	1.70	0.16	18.60	0.666	0.95	0.51	1.79	0.879	0.92	0.55	1.53	0.743	3.25	0.53	19.96	0.204	0.51	0.23	1.15	0.103
TeCA only	Depression	1.24	1.19	1.30	<0.001	1.41	1.22	1.63	<0.001	0.89	0.71	1.12	0.322	1.15	1.08	1.23	<0.001	1.15	1.09	1.22	<0.001	1.17	1.00	1.37	0.046	1.55	1.44	1.68	<0.001
(1,815 / 581,088)	TeCA only	1.64	1.23	2.19	0.001	2.07	0.89	4.85	0.093	1.51	0.49	4.60	0.471	1.28	0.85	1.93	0.231	1.49	1.03	2.15	0.035	1.02	0.39	2.67	0.974	1.67	1.05	2.63	0.029
	Depression x TeCA interaction	0.71	0.48	1.06	0.094	0.55	0.18	1.74	0.311	0.58	0.12	2.82	0.502	0.80	0.45	1.42	0.442	0.80	0.48	1.32	0.375	0.96	0.28	3.35	0.949	0.78	0.42	1.46	0.441
1 antidepressant only	Depression	1.25	1.20	1.31	<0.001	1.42	1.22	1.64	<0.001	0.89	0.71	1.12	0.315	1.16	1.08	1.23	<0.001	1.16	1.09	1.22	<0.001	1.18	1.01	1.37	0.042	1.56	1.45	1.69	<0.001
(39,668 / 618,941)	one_antidep only	1.45	1.36	1.54	<0.001	1.60	1.31	1.95	<0.001	1.32	1.00	1.73	0.053	1.35	1.23	1.48	<0.001	1.32	1.21	1.43	<0.001	1.31	1.04	1.64	0.022	1.66	1.48	1.86	<0.001
	Depression x one_antidep interaction	0.81	0.73	0.89	<0.001	0.69	0.51	0.93	0.015	0.92	0.58	1.44	0.706	0.79	0.68	0.91	0.001	0.88	0.77	0.99	0.037	0.87	0.62	1.21	0.409	0.70	0.59	0.83	<0.001
>1 antidepressant	Depression	1.24	1.19	1.30	<0.001	1.41	1.22	1.63	<0.001	0.89	0.71	1.12	0.312	1.15	1.08	1.23	<0.001	1.15	1.09	1.22	<0.001	1.17	1.00	1.37	0.045	1.55	1.43	1.67	<0.001
(5,000 / 584,273)	>1 antidep	1.63	1.34	1.98	<0.001	1.48	0.83	2.66	0.188	1.62	0.67	3.91	0.285	1.33	1.00	1.77	0.047	1.49	1.17	1.90	0.001	1.85	1.05	3.26	0.034	2.10	1.51	2.90	<0.001
	Depression x two_antidep interaction	0.76	0.59	0.97	0.028	1.00	0.47	2.14	0.996	0.79	0.26	2.39	0.669	0.94	0.66	1.35	0.755	0.71	0.52	0.98	0.036	0.46	0.20	1.03	0.059	0.72	0.48	1.07	0.108
1 class only	Depression	1.25	1.20	1.31	<0.001	1.42	1.22	1.64	<0.001	0.89	0.71	1.11	0.307	1.16	1.08	1.23	<0.001	1.16	1.09	1.22	<0.001	1.18	1.01	1.37	0.042	1.56	1.45	1.69	<0.001
(41, 988 / 621,261)	1 class only	1.45	1.36	1.54	<0.001	1.61	1.33	1.96	<0.001	1.41	1.08	1.84	0.013	1.34	1.22	1.47	<0.001	1.31	1.21	1.42	<0.001	1.31	1.05	1.64	0.018	1.70	1.52	1.91	<0.001
	Depression x oneclass interaction	0.80	0.73	0.88	<0.001	0.70	0.52	0.94	0.016	0.88	0.57	1.36	0.563	0.79	0.69	0.91	0.001	0.86	0.76	0.97	0.017	0.85	0.61	1.18	0.325	0.68	0.58	0.80	<0.001
>1 class	Depression	1.24	1.19	1.30	<0.001	1.41	1.22	1.63	<0.001	0.89	0.71	1.12	0.32	1.15	1.08	1.23	<0.001	1.15	1.09	1.22	<0.001	1.17	1.00	1.37	0.045	1.55	1.44	1.68	<0.001
(2,680 / 581,953)	>1 class only	1.75	1.37	2.22	<0.001	1.17	0.52	2.64	0.709	0.21	0.03	1.50	0.12	1.46	1.03	2.08	0.032	1.75	1.31	2.33	<0.001	2.12	1.03	4.35	0.041	1.58	1.00	2.49	0.052
	Depression x multiclass interaction	0.89	0.65	1.22	0.449	1.35	0.47	3.90	0.581	4.25	0.43	42.25	0.217	1.06	0.67	1.66	0.81	0.75	0.51	1.12	0.16	0.41	0.14	1.21	0.107	1.30	0.75	2.25	0.352
SSRI + TCA only	Depression	1.24	1.19	1.30	<0.001	1.41	1.21	1.63	<0.001	0.89	0.71	1.12	0.32	1.15	1.08	1.23	<0.001	1.15	1.09	1.22	<0.001	1.17	1.00	1.37	0.045	1.55	1.43	1.67	<0.001
(1,048 / 580,321)	ssri_tca only	1.59	1.07	2.35	0.023	1.56	0.52	4.64	0.426	0.91	0.13	6.59	0.924	1.70	1.00	2.91	0.052	1.26	0.77	2.05	0.363	3.78	1.51	9.49	0.005	1.05	0.46	2.38	0.914
	Depression x ssri_tca interaction	0.84	0.49	1.43	0.513	1.26	0.28	5.63	0.764	.	.	.	.	0.66	0.31	1.43	0.295	0.98	0.50	1.94	0.959	0.41	0.10	1.58	0.192	1.81	0.68	4.84	0.235
SSRI + TeCA only	Depression	1.24	1.19	1.30	<0.001	1.41	1.22	1.63	<0.001	0.89	0.71	1.12	0.324	1.15	1.08	1.23	<0.001	1.15	1.09	1.22	<0.001	1.17	1.00	1.37	0.046	1.55	1.43	1.67	<0.001
(582 / 579,855)	ssri_teca only	1.41	0.88	2.27	0.155	0.35	0.05	2.62	0.309	1.26	0.17	9.29	0.823	1.38	0.70	2.69	0.352	1.20	0.62	2.32	0.582	0.76	0.20	2.96	0.695	1.78	0.76	4.19	0.185
	Depression x ssri_teca interaction	0.99	0.54	1.82	0.972	0.34	0.02	5.58	0.447	1.19	0.07	19.45	0.903	1.58	0.69	3.61	0.28	0.88	0.38	2.03	0.762	1.32	0.17	10.26	0.788	0.83	0.29	2.39	0.733

Odds ratios (OR) estimated using gerneralised estimating equations with a binomial family and logit link. Interaction term included. The referent group is children of mothers with no antidepressant exposure and no depression diagnosis.

Fully adjusted models shown - adjusted for sex, deprivation quintile, ethnicity, age, maternal age, maternal smoking status, parity, multiple birth, maternal epilepsy.

SEN, special educational needs; ASD, autism spectrum disorder; PMED, physical and medical difficulties; BESD, behavioral-emotional-social difficulties; OR, odds ratio; CI, confidence interval;

SSRI, selective serotonin reuptake inhibitor; TCA, tricyclic antidepressants; SNRI. Seretonin norpepinephrine reuptake inhibitor; TeCA, tetracyclic antidepressants.

Children exposed to other antidepressant groups were excluded from each analysis.
