# Supplementary material for: Prenatal exposure to maternal depression and antidepressants and neurodevelopmental outcomes: A population cohort study
Source: PLoS Med. 2026 Sep 29;23(9):e1004658. doi: 10.1371/journal.pmed.1004658 (PMC13623103; doi:10.1371/journal.pmed.1004658)
Supplement: Supplementary file 7 [file pmed.1004658.s007.pdf]

Prenatal exposure to maternal depression and antidepressants and neurodevelopmental outcomes: a population cohort study

Saraid McIlvride[1]*, Neha Rao[1], Sarjit Singh[2], Scott M Nelson[2], Jill P Pell[1], Michael Fleming[1]

[1]School of Health and Wellbeing, University of Glasgow, Glasgow, UK; [2]School of Medicine, Dentistry and Nursing, University of Glasgow, Glasgow, UK.

S6 Appendix. Hazard ratios for ADHD diagnosis by individual antidepressant type - all models

**SSRI only vs unexposed**	**Unadjusted**				**Partially adjusted**				**Fully adjusted**			
	HR	95% CI		P value	HR	95% CI		P value	HR	95% CI		P value
Depression	1.83	1.62	2.07	<0.001	1.76	1.56	1.99	<0.001	1.57	1.37	1.80	<0.001
SSRI only	1.80	1.47	2.21	<0.001	1.72	1.40	2.11	<0.001	1.76	1.41	2.20	<0.001
Depression x SSRI interaction	0.72	0.54	0.95	0.021	0.74	0.56	0.98	0.036	0.65	0.47	0.88	0.006
Observations	147,372				147,345				130,488			
Number of events	2,480				2,480				2,061			
N exposed SSRI only	9,044				9,043				8,122			

**Citalopram only vs unexposed**	**Unadjusted**				**Partially adjusted**				**Fully adjusted**			
	HR	95% CI		P value	HR	95% CI		P value	HR	95% CI		P value
Depression	1.83	1.62	2.07	<0.001	1.76	1.55	1.99	<0.001	1.56	1.36	1.78	<0.001
Citalopram only	1.60	1.19	2.14	0.002	1.53	1.14	2.05	0.005	1.62	1.19	2.22	0.002
Depression x citalo interaction	0.73	0.50	1.08	0.115	0.77	0.52	1.14	0.186	0.68	0.45	1.04	0.072
Observations	142,680				142,654				126,296			
Number of events	2,340				2,340				1,956			
N exposed citalopram only	4,352				4,352				3,930			

**Fluoxetine only vs unexposed**	**Unadjusted**				**Partially adjusted**				**Fully adjusted**			
	HR	95% CI		P value	HR	95% CI		P value	HR	95% CI		P value
Depression	1.83	1.62	2.07	<0.001	1.76	1.55	1.99	<0.001	1.56	1.36	1.78	<0.001
fluox only	2.38	1.69	3.36	<0.001	2.25	1.59	3.18	<0.001	2.31	1.58	3.36	<0.001
Depression x fluox interaction	0.58	0.36	0.92	0.02	0.58	0.37	0.93	0.024	0.46	0.27	0.78	0.004
Observations	140,645				140,619				124,404			
Number of events	2,299				2,299				1,915			
N exposed fluoxetine only	2,317				2,317				2,038			

Sertraline only vs unexposed	Unadjusted				Partially adjusted				Fully adjusted			
	HR	95% CI		P value	HR	95% CI		P value	HR	95% CI		P value
Depression	1.83	1.62	2.07	<0.001	1.76	1.56	1.99	<0.001	1.55	1.35	1.77	<0.001
sert only	1.59	0.85	2.95	0.144	1.50	0.81	2.81	0.2	1.33	0.66	2.68	0.425
Depression x sert interaction	0.95	0.45	1.99	0.891	0.98	0.47	2.06	0.958	1.01	0.44	2.34	0.975
Observations	139,849				139,823				123,764			
Number of events	2,256				2,256				1,884			
N exposed sertraline only	1,521				1,521				1,398			

TCA only vs unexposed	Unadjusted				Partially adjusted				Fully adjusted			
	HR	95% CI		P value	HR	95% CI		P value	HR	95% CI		P value
Depression	1.83	1.62	2.07	<0.001	1.76	1.55	1.99	<0.001	1.55	1.35	1.77	<0.001
TCA only	2.44	1.60	3.72	<0.001	2.30	1.51	3.51	<0.001	2.57	1.65	4.01	<0.001
Depression x TCA interaction	0.75	0.35	1.59	0.448	0.71	0.33	1.51	0.374	0.56	0.24	1.32	0.183
Observations	139,189				139,163				123,140			
Number of events	2,253				2,253				1,884			
N exposed TCA only	861				861				774			

Amirtiptyline only vs unexposed	Unadjusted				Partially adjusted				Fully adjusted			
	HR	95% CI		P value	HR	95% CI		P value	HR	95% CI		P value
Depression	1.83	1.62	2.07	<0.001	1.76	1.55	1.99	<0.001	1.55	1.35	1.77	<0.001
amitrip only	1.95	1.15	3.30	0.013	1.88	1.11	3.18	0.019	2.13	1.23	3.69	0.007
Depression x amitrip interaction	1.00	0.36	2.78	0.994	0.98	0.36	2.72	0.975	0.61	0.18	2.11	0.432
Observations	138,977				138,951				122,953			
Number of events	2,240				2,240				1,873			
N exposed amitriptyline only	649				649				587			

SNRI only vs unexposed	Unadjusted				Partially adjusted				Fully adjusted			
	HR	95% CI		P value	HR	95% CI		P value	HR	95% CI		P value
Depression	1.83	1.62	2.07	<0.001	1.76	1.55	1.99	<0.001	1.55	1.35	1.77	<0.001
SNRI only	1.93	0.86	4.30	0.109	1.84	0.82	4.13	0.139	2.09	0.86	5.07	0.104
Depression x SNRI interaction	0.36	0.09	1.46	0.154	0.38	0.09	1.52	0.17	0.44	0.11	1.86	0.265
Observations	138,713				138,687				122,708			
Number of events	2,230				2,230				1,865			
N exposed SNRI only	385				385				342			

TeCA only	Unadjusted				Partially adjusted				Fully adjusted			
	HR	95% CI		P value	HR	95% CI		P value	HR	95% CI		P value
Depression	1.83	1.62	2.07	<0.001	1.76	1.55	1.99	<0.001	1.6	1.4	1.8	<0.001
TeCA only	2.01	0.91	4.48	0.086	1.78	0.80	3.99	0.16	2.1	1.0	4.8	0.063
Depression x TeCA interaction	0.79	0.29	2.18	0.646	0.84	0.30	2.34	0.74	0.7	0.2	1.9	0.453
Observations	138,851				138,825				122,835			
Number of events	2,237				2,237				1,872			
N exposed TeCA only	523				523				469			

1 antidepressant only	Unadjusted				Partially adjusted				Fully adjusted			
	HR	95% CI		P value	HR	95% CI		P value	HR	95% CI		P value
Depression	1.83	1.62	2.07	<0.001	1.76	1.56	1.99	<0.001	1.57	1.37	1.80	<0.001
one_antidep only	1.89	1.58	2.27	<0.001	1.80	1.51	2.16	<0.001	1.90	1.56	2.31	<0.001
Depression x one_antidep interaction	0.69	0.53	0.90	0.005	0.71	0.55	0.93	0.011	0.61	0.46	0.81	0.001
Observations	148,607				148,581				131,582			
Number of events	2,521				2,521				2,097			
N exposed 1 antidepressant only	10,279				10,279				9,216			

>1 antidepressant	Unadjusted				Partially adjusted				Fully adjusted			
	HR	95% CI		P value	HR	95% CI		P value	HR	95% CI		P value
Depression	1.83	1.62	2.07	<0.001	1.76	1.56	1.99	<0.001	1.55	1.35	1.78	<0.001
>1 antidep	2.06	1.19	3.56	0.01	1.90	1.09	3.28	0.023	1.79	0.99	3.26	0.056
Depression x two_antidep interaction	0.66	0.34	1.29	0.224	0.70	0.35	1.37	0.292	0.76	0.36	1.58	0.461
Observations	139,656				139,629				123,563			
Number of events	2,261				2,261				1,891			
N exposed >1 antidepressant	1,328				1,327				1,197			

1 class only	Unadjusted				Partially adjusted				Fully adjusted			
	HR	95% CI		P value	HR	95% CI		P value	HR	95% CI		P value
Depression	1.83	1.62	2.07	<0.001	1.76	1.56	1.99	<0.001	1.57	1.37	1.80	<0.001
oneclass only	1.89	1.59	2.26	<0.001	1.81	1.51	2.15	<0.001	1.90	1.57	2.30	<0.001
Depression x oneclass interaction	0.69	0.53	0.89	0.004	0.71	0.55	0.91	0.008	0.61	0.46	0.80	<0.001
Observations	149,206				149,179				132,121			
Number of events	2,538				2,538				2,112			
N exposed 1 class only	10,878				10,877				9,755			

>1 class	Unadjusted				Partially adjusted				Fully adjusted			
	HR	95% CI		P value	HR	95% CI		P value	HR	95% CI		P value
Depression	1.83	1.62	2.07	<0.001	1.76	1.55	1.99	<0.001	1.55	1.35	1.78	<0.001
>1 class only	2.16	1.08	4.34	0.03	1.92	0.95	3.87	0.068	1.67	0.75	3.76	0.212
Depression x multiclass interaction	0.68	0.28	1.61	0.378	0.78	0.32	1.86	0.57	0.94	0.35	2.51	0.902
Observations	139,057				139,031				123,024			
Number of events	2,244				2,244				1,876			
N exposed >1 class	729				729				658			

SSRI+TCA only	Unadjusted				Partially adjusted				Fully adjusted			
	HR	95% CI		P value	HR	95% CI		P value	HR	95% CI		P value
Depression	1.83	1.62	2.07	<0.001	1.76	1.55	1.99	<0.001	1.55	1.35	1.77	<0.001
ssri_tca only	0.66	0.09	4.67	0.678	0.60	0.08	4.28	0.607	0.68	0.09	4.90	0.702
Depression x ssri_tca interaction	2.50	0.30	20.68	0.395	2.97	0.35	24.97	0.317	3.13	0.37	26.41	0.295
Observations	138,585				138,559				122,600			
Number of events	2,228				2,228				1,864			
N exposed SSRI+TCA only	257				257				234			

Cox proportional hazards model with robust standard errors including an interaction term between maternal depression and antidepressant exposure.
Women exposed to more than one antidepressant type are excluded.
Partially adjusted model includes child sex, deprivation quintile, ethnicity and age.
Fully adjusted model additionally includes maternal age, maternal smoking, parity, multiple birth and maternal epilepsy.
