# Supplementary material for: Prenatal exposure to maternal depression and antidepressants and neurodevelopmental outcomes: A population cohort study
Source: PLoS Med. 2026 Sep 29;23(9):e1004658. doi: 10.1371/journal.pmed.1004658 (PMC13623103; doi:10.1371/journal.pmed.1004658)
Supplement: Supplementary file 8 [file pmed.1004658.s008.pdf]

Prenatal exposure to maternal depression and antidepressants and neurodevelopmental outcomes: a population cohort study

Saraid McIlvride[1]*, Neha Rao[1], Sarjit Singh[2], Scott M Nelson[2], Jill P Pell[1], Michael Fleming[1]

[1]School of Health and Wellbeing, University of Glasgow, Glasgow, UK; [2]School of Medicine, Dentistry and Nursing, University of Glasgow, Glasgow, UK.

S7 Appendix

Table A. Predicted probabilities of SEN diagnosis by antidepressant type and maternal depression status - unadjusted model

		**All-cause SEN**			
		PP[a]	95% CI		P value
**SSRI only**	No depression, unexposed	19.6	19.5	19.8	<0.001
	No depression, SSRI only	27.8	26.5	29.0	<0.001
	Depression, unexposed	26.5	25.7	27.2	<0.001
	Depression, SSRI only	28.7	27.6	29.9	<0.001
**Citalopram only**	No depression, unexposed	19.6	19.5	19.8	<0.001
	No depression, citalo only	26.3	24.6	28.0	<0.001
	Depression, unexposed	26.5	25.7	27.2	<0.001
	Depression, citalo only	29.0	27.4	30.7	<0.001
**Fluoxetine only**	No depression, unexposed	19.6	19.5	19.8	<0.001
	No depression, fluox only	28.8	26.2	31.4	<0.001
	Depression, unexposed	26.5	25.7	27.2	<0.001
	Depression, fluox only	29.8	27.6	32.0	<0.001
**Sertraline only**	No depression unexposed	19.6	19.5	19.8	<0.001
	No depression sert only	29.0	25.4	32.6	<0.001
	Depression unexposed	26.5	25.7	27.2	<0.001
	Depression sert only	26.6	23.7	29.4	<0.001
**TCA only**	No depression unexposed	19.6	19.5	19.8	<0.001
	No depression TCA only	25.2	22.1	28.3	<0.001
	Depression unexposed	26.5	25.7	27.2	<0.001
	Depression TCA only	37.3	31.5	43.2	<0.001
**Amitriptyline only**	No depression unexposed	19.6	19.5	19.8	<0.001
	No depression amitrip only	23.6	20.2	26.9	<0.001
	Depression unexposed	26.5	25.7	27.2	<0.001
	Depression amitrip only	37.2	28.8	45.5	<0.001
**SNRI only**	No depression unexposed	19.6	19.5	19.8	<0.001
	No depression SNRI only	32.0	26.5	37.6	<0.001
	Depression unexposed	26.5	25.7	27.2	<0.001
	Depression SNRI only	35.8	29.0	42.6	<0.001
**TeCA only**	No depression unexposed	19.6	19.5	19.8	<0.001
	No depression TeCA only	33.0	27.5	38.6	<0.001
	Depression unexposed	26.5	25.7	27.2	<0.001
	Depression TeCA only	31.7	26.5	36.8	<0.001
**1 antidepressant only**	No depression unexposed	19.6	19.5	19.8	<0.001
	No depression one_antidep only	27.8	26.6	28.9	<0.001
	Depression unexposed	26.5	25.7	27.2	<0.001
	Depression one_antidep only	29.7	28.6	30.9	<0.001
**>1 antidepressant**	No depression unexposed	19.6	19.5	19.8	<0.001
	No depression two_antidep only	30.3	26.7	34.0	<0.001
	Depression unexposed	26.5	25.7	27.2	<0.001
	Depression two_antidep only	29.9	27.1	32.7	<0.001
**1 class only**	No depression unexposed	19.6	19.5	19.8	<0.001
	No depression oneclass only	27.7	26.6	28.9	<0.001
	Depression unexposed	26.5	25.7	27.2	<0.001
	Depression oneclass only	29.4	28.4	30.5	<0.001
**>1 class**	No depression unexposed	19.6	19.5	19.8	<0.001
	No depression multiclass only	32.2	27.6	36.8	<0.001
	Depression unexposed	26.5	25.7	27.2	<0.001
	Depression multiclass only	34.1	30.0	38.2	<0.001
**SSRI+TCA only**	No depression unexposed	19.6	19.5	19.8	<0.001
	No depression ssri_tca only	29.4	22.0	36.8	<0.001
	Depression unexposed	26.5	25.7	27.2	<0.001
	Depression ssri_tca only	29.0	22.2	35.8	<0.001
**SSRI+TeCA only**	No depression unexposed	19.6	19.5	19.8	<0.001
	No depression ssri_teca only	31.7	21.6	41.7	<0.001
	Depression unexposed	26.5	25.7	27.2	<0.001
	Depression ssri_teca only	35.2	27.0	43.4	<0.001

[a]Cases per 100 children. Predicted probabilities (PP) represent the estimated probability of a SEN diagnosis for each combination of maternal depression and antidepressant exposure.
Depression (record of depression) and antidepressants included in the GEE model as binary terms, referent to unexposed.
An interaction term between maternal depression and antidepressant exposure is included. CI, confidence interval; SEN, special educational need;
ASD, autistic spectrum disorder; PMED, physical & medical difficulties; BESD, behavioral-emotional-social; SSRI, selective serotonin reuptake inhibitors;
TCAs, tricyclic antidepressants; SNRIs, serotonin norepinephrine reuptake inhibitors; TeCAs, tetracyclic antidepressants.

Table B. Predicted probabilities of SEN diagnosis by antidepressant type and maternal depression status - partially adjusted model

		All-cause SEN			
		PP[a]	95% CI		P value
**SSRI only**	No depression, unexposed	19.8	19.6	20.0	<0.001
	No depression, SSRI only	27.2	26.0	28.5	<0.001
	Depression, unexposed	25.3	24.6	26.0	<0.001
	Depression, SSRI only	27.6	26.6	28.7	<0.001
**Citalopram only**	No depression, unexposed	19.8	19.6	19.9	<0.001
	No depression, citalo only	25.8	24.2	27.5	<0.001
	Depression, unexposed	25.3	24.6	26.0	<0.001
	Depression, citalo only	28.0	26.5	29.5	<0.001
**Fluoxetine only**	No depression, unexposed	19.8	19.6	19.9	<0.001
	No depression, fluox only	27.9	25.4	30.4	<0.001
	Depression, unexposed	25.3	24.6	26.0	<0.001
	Depression, fluox only	28.3	26.3	30.3	<0.001
**Sertraline only**	No depression unexposed	19.7	19.6	19.9	<0.001
	No depression sert only	28.8	25.4	32.2	<0.001
	Depression unexposed	25.3	24.6	26.0	<0.001
	Depression sert only	26.0	23.3	28.7	<0.001
**TCA only**	No depression unexposed	19.7	19.6	19.9	<0.001
	No depression TCA only	24.4	21.5	27.3	<0.001
	Depression unexposed	25.3	24.6	26.0	<0.001
	Depression TCA only	34.0	28.5	39.5	<0.001
**Amitriptyline only**	No depression unexposed	19.7	19.6	19.9	<0.001
	No depression amitrip only	23.2	20.0	26.4	<0.001
	Depression unexposed	25.3	24.6	26.0	<0.001
	Depression amitrip only	35.1	27.2	43.0	<0.001
**SNRI only**	No depression unexposed	19.7	19.6	19.9	<0.001
	No depression SNRI only	30.5	25.1	35.8	<0.001
	Depression unexposed	25.3	24.6	26.0	<0.001
	Depression SNRI only	34.3	28.1	40.5	<0.001
**TeCA only**	No depression unexposed	19.7	19.6	19.9	<0.001
	No depression TeCA only	30.4	25.2	35.7	<0.001
	Depression unexposed	25.3	24.6	26.0	<0.001
	Depression TeCA only	29.8	24.8	34.8	<0.001
**1 antidepressant only**	No depression unexposed	19.8	19.6	20.0	<0.001
	No depression one_antidep only	27.1	26.0	28.2	<0.001
	Depression unexposed	25.4	24.7	26.0	<0.001
	Depression one_antidep only	28.5	27.5	29.6	<0.001
**>1 antidepressant**	No depression unexposed	19.7	19.6	19.9	<0.001
	No depression two_antidep only	29.4	25.8	32.9	<0.001
	Depression unexposed	25.3	24.6	26.0	<0.001
	Depression two_antidep only	28.8	26.1	31.4	<0.001
**1 class only**	No depression unexposed	19.8	19.6	20.0	<0.001
	No depression oneclass only	27.1	26.0	28.2	<0.001
	Depression unexposed	25.4	24.7	26.1	<0.001
	Depression oneclass only	28.2	27.2	29.2	<0.001
**>1 class**	No depression unexposed	19.7	19.6	19.9	<0.001
	No depression multiclass only	30.6	26.2	35.0	<0.001
	Depression unexposed	25.3	24.6	26.0	<0.001
	Depression multiclass only	33.3	29.4	37.3	<0.001
**SSRI+TCA only**	No depression unexposed	19.7	19.5	19.9	<0.001
	No depression ssri_tca only	28.6	21.4	35.8	<0.001
	Depression unexposed	25.3	24.6	26.0	<0.001
	Depression ssri_tca only	29.0	22.5	35.5	<0.001
**SSRI+TeCA only**	No depression unexposed	19.7	19.6	19.9	<0.001
	No depression ssri_teca only	30.0	21.2	38.8	<0.001
	Depression unexposed	25.3	24.6	26.0	<0.001
	Depression ssri_teca only	32.5	24.8	40.2	<0.001

[a]Cases per 100 children. Predicted probabilities (PP) represent the estimated probability of a SEN diagnosis for each combination of maternal depression and antidepressant exposure.

Depression (record of depression) and antidepressants included in the GEE model as binary terms.

An interaction term between maternal depression and antidepressant exposure is included.

Partially adjusted model - adjusted for sex, deprivation quintile, ethnicity, age.

CI, confidence interval; SEN, special educational need; ASD, autistic spectrum disorder; PMED, physical & medical difficulties; BESD, behavioral-emotional-social;

SSRI, selective serotonin reuptake inhibitors; TCAs, tricyclic antidepressants; SNRIs, serotonin norepinephrine reuptake inhibitors; TeCAs, tetracyclic antidepressants.

Table C. Marginal effects of antidepressant exposure on probability of SEN diagnosis by maternal depression status - unadjusted model

		All-cause SEN			
		ME[b]	95% CI		P value
**SSRI only**	At Depression=0	8.1	6.8	9.4	<0.001
	At Depression=1	2.3	0.9	3.6	0.001
**Citalopram only**	At Depression=0	6.6	4.9	8.4	<0.001
	At Depression=1	2.6	0.8	4.4	0.004
**Fluoxetine only**	At Depression=0	9.1	6.5	11.7	<0.001
	At Depression=1	3.4	1.1	5.6	0.004
**Sertraline only**	At Depression=0	9.3	5.7	12.9	<0.001
	At Depression=1	0.1	-2.8	3.0	0.938
**TCA only**	At Depression=0	5.6	2.5	8.7	<0.001
	At Depression=1	10.9	5.0	16.8	<0.001
**Amitriptyline only**	At Depression=0	3.9	0.6	7.2	0.021
	At Depression=1	10.7	2.3	19.1	0.013
**SNRI only**	At Depression=0	12.4	6.8	17.9	<0.001
	At Depression=1	9.4	2.5	16.2	0.007
**TeCA only**	At Depression=0	13.4	7.8	18.9	<0.001
	At Depression=1	5.2	0.0	10.4	0.049
**1 antidepressant only**	At Depression=0	8.1	6.9	9.3	<0.001
	At Depression=1	3.3	2.0	4.6	<0.001
**>1 antidepressant**	At Depression=0	10.7	7.0	14.4	<0.001
	At Depression=1	3.5	0.6	6.3	0.018
**1 class only**	At Depression=0	8.1	7.0	9.2	<0.001
	At Depression=1	3.0	1.7	4.3	<0.001
**>1 class**	At Depression=0	12.5	7.9	17.1	<0.001
	At Depression=1	7.7	3.5	11.8	<0.001
**SSRI+TCA only**	At Depression=0	9.8	2.3	17.2	0.01
	At Depression=1	2.6	-4.3	9.4	0.462
**SSRI+TeCA only**	At Depression=0	12.0	2.0	22.1	0.019
	At Depression=1	8.8	0.5	17.0	0.037

Table D. Marginal effects of antidepressant exposure on probability of SEN diagnosis by maternal depression status - partially adjusted model

		All-cause SEN			
		ME[b]	95% CI		P value
**SSRI only**	At Depression=0	7.5	6.2	8.7	<0.001
	At Depression=1	2.3	1.0	3.6	<0.001
**Citalopram only**	At Depression=0	6.1	4.4	7.7	<0.001
	At Depression=1	2.7	1.0	4.4	0.002
**Fluoxetine only**	At Depression=0	8.2	5.7	10.6	<0.001
	At Depression=1	3.0	0.8	5.1	0.006
**Sertraline only**	At Depression=0	9.1	5.6	12.5	<0.001
	At Depression=1	0.7	-2.1	3.5	0.631
**TCA only**	At Depression=0	4.7	1.7	7.6	0.002
	At Depression=1	8.7	3.2	14.2	0.002
**Amitriptyline only**	At Depression=0	3.5	0.3	6.7	0.033
	At Depression=1	9.8	1.9	17.7	0.015
**SNRI only**	At Depression=0	10.7	5.4	16.1	<0.001
	At Depression=1	9.0	2.8	15.2	0.004
**TeCA only**	At Depression=0	10.7	5.4	15.9	<0.001
	At Depression=1	4.5	-0.6	9.5	0.082
**1 antidepressant only**	At Depression=0	7.3	6.2	8.4	<0.001
	At Depression=1	3.2	1.9	4.4	<0.001
**>1 antidepressant**	At Depression=0	9.6	6.1	13.2	<0.001
	At Depression=1	3.5	0.8	6.2	0.012
**1 class only**	At Depression=0	7.3	6.2	8.4	<0.001
	At Depression=1	2.9	1.7	4.1	<0.001
**>1 class**	At Depression=0	10.8	6.4	15.2	<0.001
	At Depression=1	8.0	4.0	12.0	<0.001
**SSRI+TCA only**	At Depression=0	8.9	1.7	16.1	0.015
	At Depression=1	3.7	-2.8	10.3	0.265
**SSRI+TeCA only**	At Depression=0	10.2	1.4	19.0	0.023
	At Depression=1	7.2	-0.5	14.9	0.067

[b]Additional cases per 100 children, relative to no antidepressants.

Marginal effects represent the difference in predicted probabilities, estimated separately for children of mothers with and without a depression diagnosis.

Depression (record of depression) and antidepressants included in the GEE model as binary terms.

Partially adjusted model- adjusted for sex, deprivation quintile, ethnicity and age.

An interaction term between maternal depression and antidepressant exposure is included.

CI, confidence interval; SEN, special educational need; ASD, autistic spectrum disorder; PMED, physical & medical difficulties; BESD, behavioral-emotional-social;

SSRI, selective serotonin reuptake inhibitors; TCAs, tricyclic antidepressants; SNRIs, serotonin norepinephrine reuptake inhibitors; TeCAs, tetracyclic antidepressants.
