# Supplementary material for: Prenatal exposure to maternal depression and antidepressants and neurodevelopmental outcomes: A population cohort study
Source: PLoS Med. 2026 Sep 29;23(9):e1004658. doi: 10.1371/journal.pmed.1004658 (PMC13623103; doi:10.1371/journal.pmed.1004658)
Supplement: Supplementary file 9 [file pmed.1004658.s012.pdf]

# Response to Reviewers

We thank the reviewers for their continued engagement with our manuscript and for their constructive feedback. We have carefully considered all comments raised in this round of review. Below we provide point-by-point responses (in red) to each comment, with corresponding line numbers referencing the clean version of the revised manuscript.

We recognise that a key concern shared across reviewers relates to the appropriateness of making comparative statements between exposure groups (e.g. treated vs untreated depression) when estimates are derived from separate models compared against a common reference group. We have addressed this substantively by restructuring our analyses: the interaction model results (previously in Table S5) have been promoted to the main text, and this model has been applied to the other analyses (causes of SEN and individual antidepressant medications/class). Comparative language throughout the manuscript has been revised accordingly. We believe these changes significantly strengthen the manuscript.

---

**Reviewer #1**

***1.*** *I had requested clarity around the causal question being asked - in particular about the timing of the outcome. This hasn't really been provided: further description of the analysis method has been provided (which is appreciated), but the research question of interest remains opaque. What is the research question that these methods have been applied to address? The time frame for the outcome needs to be provided: e.g. any SEN etc diagnosed during a child's school years. Without a clear specification of the research question it is difficult to determine whether the research methods used to answer that question are appropriate.*

We apologise that this was not sufficiently clear in the previous revision. We have now explicitly stated the research question in both the Introduction (lines 98-100) and Methods (lines 191-193) sections. Specifically, our research question is: "Is prenatal exposure to maternal depression and/or antidepressant medication associated with increased risk of special educational need (SEN) identified during childhood (up to age 12)?".

Children enter the cohort at birth and are followed until the study end date (2022), or they leave the Welsh education system, whichever comes first. The pupil level annual school census (PLASC) records SEN status (yes/no) annually for every child, as well as individual causes of SEN, and GEE models were used to account for the repeated annual observations per child. This approach captures any SEN diagnosed during the child's school years, without imposing an arbitrary age cut-off. We have revised the Methods section to make this timeframe explicit (lines 139-142).

***2.*** *The abstract states that "Untreated maternal depression was associated with higher risk of SEN … than unexposed children. Prenatal exposure to antidepressants and treated depression … were associated with even higher risks". Comparative statements such as these ("were associated with even higher risks") can only be made if the estimates all come from the same statistical model. However, I believe that these estimates come from fitting models to different subsets of the data. If the authors wish to compare results for these different exposure types, they must use the results of the analyses presented in Table S5 (the model including the interaction term - please note that I do not think testing the statistical significance of the interaction term is a useful thing to do). The treatment effects in different subgroups can be obtained as I noted in my previous review. A similar comment*

*holds for the rest of the comparative statements in the Methods and findings section of the abstract.*

We accept this point and agree that direct comparative statements between exposure groups require estimates from a single model. The interaction model (previously Table S5) has been promoted to the main results (Table 2). This model has now been applied to the analyses of individual antidepressants and antidepressant classes (Figure 1). Margins command has been used as suggested to discern the effects of antidepressants and depression and the combination, to enable valid between-group interactions (Tables 3 and 5). We have restructured the Results section accordingly (lines 265 onwards).

All comparative language in the Abstract, Author Summary, and Results has been revised. Where we describe differences between exposure groups (e.g. treated vs untreated depression), we now base these statements on the interaction model estimates and present the relevant contrasts with 95% confidence intervals.

Finally, we have removed the test of statistical significance for the interaction term, as the reviewer suggests. Instead, we present the subgroup-specific estimates and marginal effects derived from the unified model, which are more informative for clinical interpretation.

***3.*** *Estimates presented in the author summary should be accompanied by confidence intervals.*

Thank you, we have added 95% confidence intervals to all estimates reported in the Author Summary (lines 69-73).

***4.*** *It is stated that small p-values were used to justify the inclusion of variables in models as confounders, however, this is not the recommended approach for the selection of variables to include in regression models. Instead, the modern approach to the selection of variables for inclusion in regression models to adjust for recommends considering causal diagrams and confounding pathways.*

We appreciate this important methodological point. We wish to clarify that our selection of confounders was, in practice, informed by clinical and epidemiological knowledge of the causal structure, that is, variables known or plausibly related to both maternal depression/antidepressant use and child SEN. The p-values in Table 1 were included to describe the data rather than to formally justify confounder selection. We accept, however, that our original description of the confounder selection process was misleading in suggesting a purely data-driven approach.

We have revised the Methods section to describe our confounder selection as being based on a priori clinical knowledge of the causal structure, consistent with DAG-based reasoning. We have included a DAG in the Supplementary Material (S2 Figure in S1 Appendix) to make the assumed causal relationships explicit. The p-values in Table 1 are retained as descriptive summaries of group differences but are no longer cited as the basis for confounder selection (lines 158-161).

***5.*** *This is also related to my comment regarding inclusion of "potential mediators": something may still be a mediator (i.e. on the causal pathway between the exposure and outcome of interest) even if there is no significant attenuation of effect. Thus the analyses where "potential mediators" (which I take to mean variables that the authors may believe to be on the causal pathway) should not be conducted; I strongly recommend that these analyses be removed.*

We accept this recommendation, and have removed the mediator-adjusted models from the manuscript.

---

**Reviewer #2**

We thank the reviewer for their positive comments and continued constructive feedback.

***1.** In the abstract I don't think that there is adequate description of the methods.*

We have expanded the methods description in the Abstract to include the data sources (linked primary care, maternity, and education records) and more details of the statistical approaches used (GEE models, Cox proportional hazards regression, absolute risk estimates; lines 21-22, 28-34).

***2.** There is inadequate description of the method of handling missing data in the methods section. The authors state that complete case analysis was used but without justification for this.*

We apologise for the lack of clarity. Our approach to missing data was twofold. For three non-ordered categorical confounders (maternal smoking, ethnicity, and deprivation quintile) with higher rates of missingness, we used the missing indicator method, where missing values were included as a separate category. This approach was chosen because the data were unlikely to be missing completely at random (for example, mothers who smoke may be less likely to disclose their smoking status). For remaining variables, complete case analysis was used; missingness in these variables was low (<5%) and thus unlikely to introduce substantial bias. We have expanded the Methods section to provide this justification more clearly, including the rationale for the approach taken for each type of variable (lines 168-174).

***3.** Table 1 could be improved by adding a row above the categories of exposed and then for unexposed and total, to make it explicitly clear that there are multiple exposure groups.*

Table 1 has now been revised in line with the new analysis, and therefore the exposure groups are exclusive, which we hope makes it clearer.

***4.** For example, in line 247 in the tracked changes manuscript adjusted has been added to the odds ratio but the abbreviation still says OR. Using aOR for this and all adjusted analyses would improve clarity.*

We have changed the abbreviation to aOR throughout the manuscript for all adjusted analyses, and have defined the abbreviation at first use (line 37).

***5.** Line 254 states "Results are for repeated observations per child across years" under the table. I am unclear on what this means, could the authors please provide further clarification.*

We have revised this footnote to make clear that for the GEE models, each child contributes one observation per school year (lines 274-275).

***6.** From the other reviewer comments and author reply, I understand why p-values have been included in table 1. I am unclear however why they have been used in the main*

*analysis continued throughout the paper, when 95% CI are reported. Can the authors please provide further justification for this?*

We agree that when 95% confidence intervals are reported, p-values add limited additional information for the main analyses and so these have been removed. P-values are retained in Table 1 as descriptive summaries of group differences.

***7.*** *From the description in the methods, I understand that all comparisons are made to the unexposed group, however from line 260 onwards in the results section, it is unclear what the comparison group is. I think it reads as though the women with depression have been compared with women with untreated depression. Can the authors please revise the wording in the results to make this distinction clearer?*

The analyses structure has now been revised following reviewer feedback, and the referent group is unexposed for all comparisons in the main analyses (i.e. children of mothers with no record of depression or antidepressant prescriptions, or anxiety/severe mental illness, anxiolytics or antipsychotic medications). This has been made explicit in the Methods section (lines 211-212) and in the Results section (lines 266, 269, 272).

Where we make comparisons between exposure groups, we now use estimates from the interaction model.

***8.*** *Discussion: I thank the authors for addressing my comments about placing their study in context of the wider literature on the topic, however, I think this still requires further attention in the most recent draft. Would the authors consider being more specific with comparisons to other studies? There are recent studies that examine the association between antidepressant exposure in pregnancy and offspring neurodevelopmental outcomes, with several employing multiple subgroup approaches such as sibling-comparison analyses and not simply univariate analysis. I don't feel that the discussion in its current form addresses these adequately.*

Thank you for the suggestion. We have revised the Discussion to include specific comparisons with studies that have employed more rigorous causal inference approaches. In particular, we now discuss sibling-comparison studies, studies employing negative control/paternal exposure designs and discontinuation designs (lines 385 onwards). We believe that the discussion is now more balanced.

---

**Reviewer #3**

We thank the reviewer for their positive assessment and continued engagement with our manuscript.

***1.*** *The only point that still needs to be reviewed is the conclusions made in between groups [i.e., Figure 1]. I still think it is not fully accurate to make conclusions about comparisons between depressed untreated and treated when you are only comparing 1) depressed untreated vs. unexposed and 2) depressed treated vs. unexposed in your analyses. The analyses you have added to S5 would be more appropriate for your main findings if you want to conclude on these specific comparisons between treated/untreated.*

We agree with this point, which was also raised by Reviewer #1. As described in our response to Reviewer #1, the interaction model results (previously in Table S5) have been promoted to the main Results section (Table 2). Where we compare exposure groups (e.g. treated vs untreated depression), we now present the relevant contrasts derived from this

unified model. The Abstract and Author Summary have also been revised to ensure all comparative statements are supported by the appropriate analysis.

## Summary of key changes

In summary, the principal changes to the manuscript in this revision are:

**1.** Promotion of the interaction model (previously Table S5) to the main Results (Table 2), enabling valid between-group comparisons.

**2.** Unified interaction model used for individual antidepressants/classes analyses (Figure 2).

**3.** Absolute risk difference estimated for all-cause SEN (previously Table S7) and types of SEN and ADHD, as well as individual antidepressants and classes (Tables 3-5).

**4.** Revision of all comparative language (Abstract, Author Summary, Results, Discussion) to ensure between-group statements are based on the unified model.

**5.** Explicit statement of the research question including the outcome timeframe (any SEN during school years, ages 4–12).

**6.** Revised confounder selection rationale based on DAG-based reasoning, with a DAG included in Supplementary Material.

**7.** Removal of mediator-adjusted models from the main analyses.

**8.** Simplified the adjusted models by including maternal epilepsy in the fully adjusted model due to negligible impact on estimates in the previous iteration of the manuscript.

**9.** Addition of 95% CIs to Author Summary; removal of p-values from main analyses; use of aOR abbreviation throughout.

**10.** Expanded methods description in Abstract; improved clarity of missing data handling; updated Table 1 to reflect exposure groups in unified model.

**11.** Expanded Discussion including specific engagement with sibling-comparison studies and other causal inference approaches.

**12.** Clarification of comparison groups throughout Results section.

**13.** DAG included as supplementary figure.
