# Supplementary material for: Prenatal exposure to maternal depression and antidepressants and neurodevelopmental outcomes: A population cohort study
Source: PLoS Med. 2026 Sep 29;23(9):e1004658. doi: 10.1371/journal.pmed.1004658 (PMC13623103; doi:10.1371/journal.pmed.1004658)
Supplement: Supplementary file 10 [file pmed.1004658.s013.pdf]

Author response

We thank the editor and the reviewers for their positive feedback. Please find below our responses in red to each comment, with corresponding line numbers referencing the tracked version of the revised manuscript.

Requests from Editors:
GENERAL EDITORIAL REQUESTS

* Please confirm that your title complies with PLOS Medicine's style. Your title must be nondeclarative and not a question. It should begin with main concept if possible. "Effect of" should be used only if causality can be inferred, i.e., for an RCT. Please place the study design ("A randomized controlled trial," "A retrospective study," "A modelling study," etc.) in the subtitle (ie, after a colon).

*Please change your title to: “Prenatal exposure to maternal depression and antidepressants and neurodevelopmental outcomes: a population cohort study”

The title has been changed.

*Funding statement: thank you for including your funding statement. For HDR, please consider whether all the stated sources of funding are necessary to mention for this study.

The HDR UK funders have been shortened.

*Please ensure that your Data availability statement in the metadata is the same as in the manuscript.

*ETHICS: Thank you for stating that “Access to the data was approved by the SAIL Databank Information Governance Review Panel (IGRP).” Please also provide an approval number.
This has been added (line 131).

* ETHICS: Please specify whether informed consent was written or oral. Please ensure that the research complies with the PLOS policy in full: https://eur03.safelinks.protection.outlook.com/?url=https%3A%2F%2Fjournals.plos.org%2Fplosmedicine%2Fs%2Fhuman-subjects-research%23loc-patient-privacy-and-informed-consent-for-publication&data=05%7C02%7CSaraid.McIlvride%40glasgow.ac.uk%7C6d0fa8f3b6e14e42db1d08def240f56d%7C6e725c29763a4f5081f22e254f0133c8%7C1%7C0%7C639214557990409926%7CUnknown%7CTWFpbGZsb3d8eyJFbXB0eU1hcGkiOnRydWUsIlYiOiIwLjAuMDAwMCIsIlAiOiJXaW4zMiIsIkFOIjoiTWFpbCIsIldUIjoyfQ%3D%3D%7C0%7C%7C%7C&sdata=nmCF4VxgkHLGTmAisZDT18hOBwc9LINWq70PLFNIcDI%3D&reserved=0

Sentence regarding individual consent has been added to 131-132.

ABSTRACT

* Please confirm that your abstract complies with our requirements, including format (three sections: Background, Methods and Findings, and Conclusions) and providing all the information relevant to this study type https://eur03.safelinks.protection.outlook.com/?url=https%3A%2F%2Fjournals.plos.org%2Fplosmedicine%2Fs%2Fsubmission-guidelines%23loc-abstract&data=05%7C02%7CSaraid.McIlvride%40glasgow.ac.uk%7C6d0fa8f3b6e14e42db1d08def240f56d%7C6e725c29763a4f5081f22e254f0133c8%7C1%7C0%7C639214557990425691%7CUnknown%7CTWFpbGZsb3d8eyJFbXB0eU1hcGkiOnRydWUsIlYiOiIwLjAuMDAwMCIsIlAiOiJXaW4zMiIsIkFOIjoiTWFpbCIsIldUIjoyfQ%3D%3D%7C0%7C%7C%7C&sdata=MBlcRUZxrrsNDNX0qDMUpzLOBjGwZnYUzjvxnpuP0U4%3D&reserved=0

The abstract has been edited to bring the word count to 500 words following the requested additions.

* In the last sentence of the Abstract Methods and Findings section, please describe the main limitation(s) of the study's methodology.

This has been added (lines 55 – 57).

* In the abstract, please include the important dependent variables that are adjusted for in the analyses.

This has been added (lines 36 – 37).

AUTHOR SUMMARY:
-Why was this study done: we suggest to combine the 1st and 2nd bullet point. Combined.
-Why was this study done, 3rd bullet point: we suggest to either remove this, or combine with bullet point 4. Combined.
-What did the researchers do and find: we suggest to combine bullet point 1 and 2. Combined.

* In the author summary, in the final bullet point of 'What Do These Findings Mean?', please include the main limitations of the study in non-technical language.

This has been added (Line 87-89).

GENERAL

* Please ensure that the Introduction ends with a clear description of the study question or hypothesis. Second last sentence.

* Please ensure that all abbreviations are defined at first use throughout the text. Confirmed.

* Please confirm that all numbers presented in the abstract are present and identical to numbers presented in the main manuscript text. Confirmed.

* Please include a statement regarding use or non-use of AI tools and technologies at the end of the Methods. Please specify the name(s) and version(s) of any tool(s) used and a description of their use, including how the AI-assisted or generated content was evaluated by authors, and a clear statement of which aspects of the study, article contents, data, or supporting files were affected or generated

by AI tool usage. Please also include a statement specifying who retains responsibility for the final manuscript. See our guidelines for more information: https://eur03.safelinks.protection.outlook.com/?url=https%3A%2F%2Fjournals.plos.org%2Fplosmedicine%2Fs%2Fethical-publishing-practice%23loc-artificial-intelligence-tools-and-technologies&data=05%7C02%7CSaraid.McIlvride%40glasgow.ac.uk%7C6d0fa8f3b6e14e42db1d08def240f56d%7C6e725c29763a4f5081f22e254f0133c8%7C1%7C0%7C639214557990439734%7CUnknown%7CTWFpbGZsb3d8eyJFbXB0eU1hcGkiOnRydWUsIlYiOiIwLjAuMDAwMCIsIlAiOiJXaW4zMiIsIkFOIjoiTWFpbCIsIldUIjoyfQ%3D%3D%7C0%7C%7C%7C&sdata=qVgtP3eV1O81lRwbP2zkQL3tqjUQqwKjj5jYq0b8EcA%3D&reserved=0.

A new section 'AI Tools' has been added (lines 253-255).

A statement regarding responsibility has been added to the Author Contributions section (lines 535-536) : "SM and MF are guarantors of the study and take responsibility for the integrity of the data and the accuracy of the data analysis."

* Statistical reporting: Please revise throughout the manuscript, including tables and figures.
- Please report statistical information as follows to improve clarity for the reader ""22% (95% CI [13,28]; p</=)"". Parentheses have been added around the CIs and commas used instead of hyphens. % have been removed from within CI square brackets.
- Please separate upper and lower bounds with commas instead of hyphens as the latter can be confused with reporting of negative values. As above.
- Please repeat statistical definitions (HR, CI etc.) for each set of parentheses. Confirmed.

* Please remove the 'conclusions' subheading from the discussion. Please also remove any other subheadings from the discussion.

Subheadings have been removed.

* Thank you for including the STROBE checklist. However, could you attach the checklist as a separate file, rather than as part of the appendix?

This has been uploaded as S1 Checklist.

FIGURES AND TABLES

* Please provide the unadjusted comparisons as well as the adjusted comparisons in all relevant Tables.

Unadjusted comparisons are presented in the supplementary information (Appendices S2-S7), a sentence to this effect has been added to the Methods section (lines 217-218) and signposted in Results (line 281).

* Please specify the variables controlled for in all relevant Tables. Confirmed.

OBSERVATIONAL, COHORT, CROSS-SECTIONAL, AND CASE CONTROL STUDIES

* Did your study have a prospective protocol or analysis plan? Please state this (either way) early in the Methods section.

a) If a prospective analysis plan (from your funding proposal, IRB or other ethics committee

submission, study protocol, or other planning document written before analyzing the data) was used in designing the study, please include the relevant prospectively written document with your revised manuscript as a Supporting Information file to be published alongside your study, and cite it in the Methods section. A legend for this file should be included at the end of your manuscript.

b) If no such document exists, please make sure that the Methods section transparently describes when analyses were planned, and when/why any data-driven changes to analyses took place.

c) In either case, changes in the analysis-- including those made in response to peer review comments-- should be identified as such in the Methods section of the paper, with rationale.

I have detailed in the Methods section 'Statistical analyses' what we planned a priori to test and what was changed in response to reviewer comments (lines 206-215).

Comments from Reviewers:

Reviewer #1: I thank the authors for their responses to my comments. I have no further suggestions.

Reviewer #2: Thank you to the authors for addressing the concerns raised by the reviewers. I think that the study is much clearer now and that the design and analysis more logical following the last review. My recommendation is to publish the manuscript. Well done to the authorship team for the work that has gone into this study. I think that it adds evidence to an important aspect of maternity care that is often neglected.

The only minor suggestion that I have is that in the abstract, line 72 be revised. I think that saying 2.9% is unclear what effect estimate this is and therefore what is meant by it.

This has been reworded in the abstract (lines 47-49 and below) and in the author summary.

"Antidepressant exposure was associated with a 6.3% absolute increase in SEN risk (95% CI [5.2%,7.4%]) among children of mothers without depression and 2.9% absolute increase (95% CI [1.7%,4.1%]) among children of mothers with depression."

Reviewer #3: Thank you all my queries have been addressed.
