# Supplementary material for: Prenatal exposure to maternal depression and antidepressants and neurodevelopmental outcomes: A population cohort study
Source: PLoS Med. 2026 Sep 29;23(9):e1004658. doi: 10.1371/journal.pmed.1004658 (PMC13623103; doi:10.1371/journal.pmed.1004658)
Supplement: Supplementary file 11 [file pmed.1004658.s011.docx]

Comments from the academic editor:

This is an important topic, but the main analyses need to be re-designed and run again.

Thank you for the opportunity to revise our article, and thank you to the reviewers for their feedback and suggestions. We have addressed their comments below, including running additional analyses as suggested by reviewer #1, which support the findings of our main analyses. We believe our original study design reflects the full picture of associations with SEN across the exposure groups, and is now strengthened with the new data showing that antidepressant exposure confers an additional modest risk over depression, hence we have included the additional results as Supplementary Tables, with our original results remaining in the main paper. Please find responses in red below, with corresponding line numbers (of tracked changes version) provided to direct to relevant sections in the manuscript.

-----------------------------------------------------------
Comments from the reviewers:

Reviewer #1: This manuscript presents an analysis of data from 167,447 children born in Wales, between 2009 to 2016, investigating the impacts of maternal depression and maternal antidepressant prescription on special educational needs outcomes. My comments focus on the statistical aspects of this analysis. Given my interpretation of the statistical analysis as described in this manuscript, it is unclear whether the conclusions drawn are supported by the data.

1.      Here there are two exposures of interest: one or more prescriptions for antidepressant medication during pregnancy or in the month prior to conception, and a maternal depression diagnosis recorded in the two years before delivery. Here it seems that separate analyses have been conducted for different combinations of these two exposures. I have questions and recommendations for analysis of these two exposures.

a.      In Table 1, the number of women with depression is stated as 25631. I would expect that the number of women with depression would be equal to the number of women with untreated depression plus the number of women with treated depression. However, this does not add up to the number of women with treated depression (7005) plus the number with untreated depression (12630). In fact, 7005+12630 = 19635, almost 6000 fewer women than are in the depression group. I don't understand why there is this difference; can this be explained?

Apologies that this was not clearer. This is because the groups are not all mutually exclusive. The ‘Depression’ group includes all women who are taking antidepressants during pregnancy but may or may not have a depression diagnosis recorded, as well as women who have a diagnosis of depression, regardless of whether they are taking antidepressants or not. We wanted to include this wider group in the analysis as it is possible that women will have depression but the diagnosis was recorded outside of our 2-year window or not at all. However, it is also possible that they could be taking antidepressants due to anxiety or another condition e.g. OCD and therefore that is why we then derived the tighter definition group of ‘treated depression’ as these are all women who have a depression diagnosis as well as taking the medication. A group exposed to antidepressants regardless of diagnosis was also included. Hence, the number of women in the depression group (25,631) equals the number of women with untreated depression (12,630) plus the number of women treated with antidepressants (13,001). This has been clarified in the methods section (lines 144-155).

b.      Separate analyses for each exposure of interest have been conducted (as far as I can tell). Instead of conducting separate analyses for each of the cohorts of interest, it would make more sense to fit a model for the outcome, including a binary term for "presence of depression" (I'll call this Dep), a binary term for "antidepressant prescription" (I'll call this Ant), and an interaction between the two. The effect "treated depression" (Dep=Yes, Ant=Yes), "Untreated depression" (Dep=Yes, Ant=No) can then be extracted directly. The effect of "Depression" can also be extracted or of "Antidepressant use" would then be obtained by marginalising over the effect of the other exposure; in Stata this would be achieved using the margins command. This approach allows direct comparison of the effects of different combinations of these two exposures. If separate regression models have been fit for different subgroups then the comparisons made between results for women with treated and untreated depression, for example, are inappropriate: it is difficult to determine whether the estimates for children exposed to treated depression differ to a large degree from those for children exposed to untreated depression if the estimates for each are based on different statistical models.

Thank you for this suggestion. We have now included an additional analysis where binary terms for antidepressant medication and depression are included in the same GEE model, which can be seen in Supplementary material (S5 Table). We also included an interaction between the two which was significant (p<0.001). Following this we performed subgroup analyses where antidepressants were regressed on SEN in those exposed to depression or unexposed to depression (S6 Table). We also calculated marginal effects as suggested (S7 Table). The results show that antidepressant exposure increases the effect size both in those with and without depression.

c.      If analyses have been conducted separately for different exposures, then statements such as "associations were stronger for treated depression than untreated depression" may not be supported by the data.

Additional analyses have been performed using a single model as described above, and direct comparisons have been made more explicit, e.g. lines 297, 302

d.      If the above suggestion for analysis aligns with the analyses that were actually conducted, then this needs to be clarified in the Statistical analyses section.

More detail on the analyses has been provided in the methods section (lines 190-205).

e.      The above suggestion does not require any change to Table 1 (although, as I note below, standardised differences rather than p-values should be used to compare groups; and as noted above there is a discrepancy in the sample sizes of the different groups)

Thanks for your comments however for the demographic table we were simply interested in whether there were any significant associations between confounders and exposures (regardless of magnitude) as a means of justifying their inclusion in the later models. We have addressed the discrepancy in sample sizes in an earlier comment.

2.      It is stated that a GEE-based approach was used to account for repeated measures on the same child; but it is unclear to me what the timing of the primary outcome of interest actually is, and how the data were structured. That is, were outcomes available for each child in each year? Was the primary question of interest SEN by age 10 (or some other age)? I have difficulty determining what the actual research question of interest is here; this requires clear definition of the exposure (this seems clearly defined), the outcome (again, clearly defined), and also the time of the outcome (at 5 years of age? 10 years? Or the time to the occurrence of the outcome?) Please define the research question of interest clearly.

The School Census is conducted annually and includes data for every child attending school at that point in time. Therefore, every schoolchild has SEN (yes/no) recorded annually. Our interest was in whether a child went on to develop any type of SEN (all cause or cause specific). Some types of SEN are recorded consistently after initial diagnosis (e.g. autism) whilst some other types of SEN can arise and then disappear again (e.g. communication problems). Analysing the outcomes on a yearly basis using a panel design and GEE analyses was beneficial as it allowed us to include all records in the analyses (maximising power) rather than try to create an artificial summary of outcomes based on different ages. It also removed the issues of different amounts of follow up (in years) for different pupils due to children entering and leaving the cohort in different years. We have used these methods in several previous papers.

3.      It is stated that maternal and child confounders were adjusted for. However, the child-level confounders included child age - child age at what event? This seems as though it could instead be on the causal pathway from exposure to outcome (e.g. the age of a child when they first have SENs could be impacted by maternal depression and use of antidepressants), so should not be adjusted for.

As described above, the School Census is conducted annually, therefore, every schoolchild has SEN (yes/no) recorded annually. Some types of SEN are recorded consistently after initial diagnosis (e.g. autism) whilst some other types of SEN can arise and then disappear again (e.g. communication problems) and diagnosis can sometimes be delayed. We used the child’s age as recorded in each school census year. As children enter the cohort in different years, some children will be followed up to older ages than others and the former will have a greater opportunity to be recorded as SEN yes. Since clinical practice (diagnosis threshold or medication use) may change over time, age at recording of SEN is a potential confounder. However, it is not on the causal pathway since disease/medication are not causally associated with the duration of follow-up and hence age at recording of SEN.

4.      When it is stated that gestational age and birthweight were truncated, does this mean that babies with ages and weights outside this region were excluded? It would be inadvisable to modify the observed values of child characteristics.

Values outside of the biologically/clinically plausible ranges were converted to missing (not modified) due to likely being erroneous. This has been clarified on line 169.

5.      Please provide some justification for the missing-treated-as-separate category approach used for the analyses.

We did not consider the deletion method (complete case analysis) to be appropriate because of the reduction in statistical power and introduction of bias due to the data being unlikely to be missing completely at random (since extreme values have greater clinical importance and are therefore less likely to be missing). Multiple imputation and the missing indicator method (as used) are more appropriate for data that are not missing at random; from a clinical perspective, an example of this in our analyses is mothers being less likely to divulge their smoking status if they are a smoker compared to a non-smoker, making this confounding variable missing not at random . The additional computational complexity of multiple imputation is warranted where the variables with missing data are exposure variables and therefore it is necessary to derive an effect size for that variable. However, in our study the variables were confounders. As such, we merely needed to adjust for their effect and hence the more straightforward missing indicator method was both appropriate and adequate.

6.      Standardised differences would be more informative than hypothesis tests for determining which confounders have large differences between exposure groups. The reason for this is that standardised differences provide some measure of the magnitude of differences, while, for a large enough sample, any difference between groups, no matter how tiny, will have a small p-value.

Thanks for your comments however for the demographic table we were simply interested in whether there were any significant associations between confounders and exposures (regardless of magnitude) as a means of justifying their inclusion in the later models.

7.      It is stated that the final stage of models included potential mediators - however, when including potential mediators, the research question being asked is no longer about the impact of an exposure on an outcome; questions of how the effect of that exposure is mediated by the outcome are being assessed, and methods appropriate for mediation analyses are required. If interest is in separating out direct and indirect effects of the exposures of interest, then a formal mediation analysis would be required.

We included them in the model to investigate whether there was any indication of mediation. Had we observed a significant attenuation of effect size we would have proceeded to formal mediation analysis. However, we did not, so this was not necessary. We have added a note to this effect in the revised manuscript (line 214-216).

8.      How were the analyses for specific antidepressants conducted? Was "antidepressant" included as a categorical variable in the outcome regression models?

Analyses for specific antidepressants were run as separate models, with the specific antidepressant coded as a binary variable (yes/no). Women taking that specific antidepressant exclusively were compared with women taking no antidepressant. Women taking other antidepressants were excluded from the analysis. This has been clarified on lines 159-160, 196-199.

Following these analyses, subgroup analyses were carried out where only women with treated depression were included in the analysis, and specific antidepressants (TCAs and SNRIs) were compared directly to SSRIs in binary GEE models. Women taking other antidepressants/combinations were excluded from the analysis.


Reviewer #2: This paper addresses maternal mental health; a hugely important aspect of maternal health that is both under recognised and under researched. Well done to the authors on this population linkage study.  This study adds valuable insight and may assist women and their care providers in weighing up treatment options to optimise pregnancy outcomes for each individual woman. However, there are several points that, in my opinion warrant further attention:

Major points:

1.      Please report according to STROBE guidelines.

A completed STROBE checklist has been added to the Supplementary Material.

2.      Can the authors please provide clearer justification for the choice of analysis (i.e. the use of several comparison groups)? The outline of the exposure groups does not include sufficient detail, and it is not clear enough that women and offspring could be included in more than one group. Further, it is confusing why the totals don't add up, for example, I would have thought that treated depression + untreated depression + comparison group = total cohort. This does not seem to be the case though. Further detail on the division of groups would improve clarity.

The comparison (referent) group is common to all analyses and comprises women who have neither a record of depression nor a record of having been prescribed antidepressants (nor anxiolytics, antipsychotics or a record of anxiety or severe mental illness).

As stated by the referee, the exposure groups are not mutually exclusive. Firstly, we defined a wide definition of depression: women with a record of depression AND/OR antidepressants. This was because it is possible that women have depression but the diagnosis was recorded outside of our 2-year window or not at all. We then focused on associations with anti-depressant medication and whether any association was likely to be due to the medication or the medication was simply a proxy measure of the condition (confounding by indication/severity). To investigate medication we used both a tighter definition of treated depression and a wider definition (antidepressants even if the women was not recorded as having the depression). The latter was included because it is also possible that they could be taking antidepressants due to anxiety or another condition e.g. OCD. There are merits and weaknesses to both tight and wide definitions so the aim was to see if the results were robust to the definitions applied.

In relation to the specific query about the numbers not adding up, this is due to the groups not being mutually exclusive and is answered in more detail above (referee 1; Q1a)

To clarify the groups/definitions, further detail has been added to methods section (lines 144-155).

3.      Can the authors please provide further detail on each of the databases included in the study? The purpose and coverage of each database, and what percentage of data collected are missing. The authors state that the primary care data are available for 83% of the population but that is all. Please elaborate.

More detail has been added to this section where the information is available (lines 109 – 124).

4.      In the results, can the authors please explain why the four exposed groups are not all represented in figure 1? Why is the depression cohort not shown?

This was not included originally as the data for the depression cohort was shown in Table 2, but it has now been added to Figure 1.

5.      I think that the discussion needs some work and would benefit from deeper interrogation of previous studies on this topic. There needs to be a more thorough discussion of this study in the context of other studies examining SEN and maternal mental health, with a clearer distinction between how this study divides exposures to maternal depression and/or antidepressant use in pregnancy.

Thank you for this suggestion, we have expanded on this in the discussion, e.g. lines 333-343.

6.      While population wide observational studies are a vital method of examining perinatal exposures and longer-term outcomes, they have inherent limitations. The authors highlight that postnatal and paternal depression could also impact outcomes. This is a very important point that warrants further discussion. Unmeasured confounders and residual confounding due to imperfect measures are likely to be present. This is a well understood, unavoidable aspect of retrospective cohort studies, particularly of perinatal exposures. Despite this, discussion of this and potential impacts on findings are important. I recommend that the authors expand this point in the limitations section.

We agree that these are important considerations and so have expanded upon confounders in the discussion section (lines 405-417).

7.      Our findings support the recommendation that SSRIs should be the first line therapy where antidepressants are indicated during pregnancy. I think this statement needs revising; it overstates findings. I would argue that the study examines the risk of SEN in exposure groups and therefore recommending medications that treat depression in not an accurate conclusion of this study.

Thank you for this perspective, we have reworded our conclusion (line 441).


Minor points:

1.      Please provide further data on the handling of missing data. Was there a statistical analysis plan a priori, and did it specify how missing data would be dealt with?

Our a priori analysis plan stated that categorical variables which had high % of missing would be analysed as a dummy variable. We have made more explicit which analyses were planned a priori in the methods section (line 173). See also our response to Referee 1, Q5 which explains why we adopted our approach to handling missing data.

2.      Mothers were excluded if their primary care records had gaps of more than 30 days during this period (30 day maximum allows for transfer of records between primary care practices). Please clarify what this means and the potential impact that it has.

Mothers were excluded if they were not continuously registered with GPs participating in SAIL throughout the study period. This was required because only 83% of primary care practices contribute to SAIL Databank. Therefore, it was possible for a woman to have records missing during a period when she registered with a different GP. Gaps of less than 30 days during this period were permitted as it could take up to 30 days to transfer records when a patient moves between primary care practices. Further detail has been added to lines 136-139.

3.      Was a drug category system used? Could the authors please provide more detail.

Medications were classified according to the British National Formulary (BNF). This has been added to the Methods section (line 130).

4.      In the exposure to specific antidepressant section of the results, could the authors please include the absolute numbers of the exposed included in each analysis? e.g. (SNRIs; n, N, %,OR 2.00, 95% CI 1.47-2.71).

This information has been added to this section (lines 293-304).

5.      The authors state Some children may have been further exposed to medication postnatally via breastmilk, however studies have shown that women with depression, treated and untreated, have low breastfeeding initiation. I would recommend that the authors review this sentence and either provide a more balanced viewpoint on this point or revise the wording.

Thank you for suggesting we look at this again. We have reviewed this and included references in the discussion which show that breastfeeding is also associated with reduced postnatal depression which we hope provides more balance on this point (lines 410-412).

Reviewer #3: Thank you very much for the opportunity to review your very interesting work. This is an under-researched area of great clinical and social importance. The approach of using multiple linked, population-based, high-quality datasets is very powerful and well executed. The manuscript is very well written and easy to understand. I do have a few points I want to raise for further clarification:

Methods:
Inclusion Criteria and Definitions
*       Given a long time-window of up to 2 years, was there an approach to assess/validate whether the mother still had depression (without antidepressants) during the pregnancy?

This is a limitation of using routinely collected data as once the GP has recorded the diagnosis, there is no requirement to re-enter it every time the patient visits the GP. For example, in our dataset, less than 1 in 5 women who had depression recorded prior to pregnancy also had it recorded during pregnancy. Therefore we included a period prior to the pregnancy (15 months) in order to include women who were recorded as being depressed but might not have visited their GP about this specifically during their pregnancy. This limitation has now been highlighted in the discussion (line 425-428).

Covariates:
*       Was there a reason as to why other maternal comorbidities (other than epilepsy) were not included as covariates (having diabetes is linked to SEN too)?

Epilepsy was included as a covariate due to the crossover in medications used in epilepsy that could also be used to treat mental health disorders.

*       Similarly for childhood comorbidities (trisomies, inherited genetic conditions, epilepsy)?

These comorbidities were considered to be on the causal pathway to the outcomes of interest rather than confounders and therefore we did not include them. Moreover, childhood comorbidities that are diagnosed post-delivery cannot have affected pre-delivery antidepressant use; while we appreciate that inherited conditions and trisomy are present in-utero, they are again unlikely to affect the clinical choices of prescribing antidepressant therapy.

Analysis:
*       For missing data (there is about 5-10% missingness according to your Table 1 in parity and APGAR scores), did you use complete case analysis? This should be stated.

Yes, we used complete case analysis overall and this has been added to the Methods section (line 209). For three non-ordered categorical variables, the missing indicator method was used to handle missing data as using the deletion method (complete case analysis) for these variables which had higher missingness would have reduced statistical power and introduced bias due to the data being unlikely to be missing completely at random (since extreme values have greater clinical importance and are therefore less likely to be missing).

Results:
*       Can you clarify if the follow up of 5.68 years is from birth or from point of entry into the cohort/school year? The following sentence "The main cohort comprised 167,477 children, born in Wales between 2009 and 2016, with mean follow-up of 5.68 years (Table 1)." suggests its from birth and thus needs clarification.

Yes, the follow up period is from birth onwards. This has been clarified on line 222.

*       Table 1: Your missing % are stated in some variables (e.g., smoking) but not in others (e.g., parity). Is there a reason why they are not presented with %s throughout - could they all be included as an additional category (as per the comment under table 1, f)?

Missing data was included as a category only for the non-ordered categorical variables, and hence these are the only groups where the % missing was included in the total.

*       Table 2: Are the number of observations greater than Table 1 due to repeated observations per child? Could this be clarified.

Yes, Table 2 is presenting the results where there are repeated observations per child. This has now been made explicit in the methods section (line 121) and legend of Table 2.

*       Not sure if you can confidently conclude on comparisons across exposure categories (e.g., the anti-depressant category had a stronger association than the depressed-only category) as each exposure category is independently compared to the undepressed population and not to each other. Especially given the confidence intervals sometimes overlap (e.g., "The association with SEN was higher for… specifically amitriptyline, and serotonin-norepinephrine reuptake inhibitors (SNRIs; OR 2.00, 95% CI 1.47-2.71) than for SSRIs (OR 1.39, 95% CI 1.31-1.48)". Further analysis using a multinomial or ordinal logistic regression with repeated measures may be able to more directly answer this.

Apologies that this was not clearer, for individual antidepressants a subgroup analysis was carried out where the antidepressants were compared directly to SSRIs (table S11) but this has been made more explicit (lines 297-299, 302-304). We have also carried out further subgroup analyses where the effect of treatment is assessed in those exposed to maternal depressed and unexposed (S6-S7 Tables).

*       Small point: in the following sentence, the untreated depression confidence intervals are missing : "behavioral-emotional-social difficulties (treated depression OR 1.80, 95% ci 1.67-1.95; untreated depression OR 1.55, 95% CI 1.55, 95% CI 1.43-1.67; Figure 1, Tables S2-4)"

Thank you for pointing this out, this has been corrected (line 268) along with errors we noticed in the values reported in the line above (line 267).

Discussion:
*       Ascertainment of depressed women during pregnancy: I think an important limitation of using healthcare records is that you may have pregnant women with depression in the community who do not present to healthcare services (this does tend to be biased towards ethnic minority groups not presenting to their healthcare providers)

We agree that some undiagnosed women will be erroneously included in the comparison/referent group. The expected effect of this will be to underestimate any effect of depression. We have added these points to the discussion (lines 429-431). We also acknowledge that under-ascertainment of depression in routine data may be differential, with lower detection among women from ethnic minority groups, reflecting known inequalities in access to and engagement with mental health services. However, pregnancy represents a period of increased contact with healthcare services, and in the UK all women should be asked about mental health as part of antenatal booking/in early pregnancy, with further assessment recommended for those who screen positive. As a result, ascertainment of depression is likely to be higher during pregnancy than at other life stages. In addition, the study population was predominantly White (94%), which may limit to some degree the impact of differential under-ascertainment by ethnicity.

*       Ascertainment of antidepressants. Another limitation is that you are using the GP records to identify antidepressants, which is the most comprehensive way, but this may miss those who are prescribed privately or directly from hospital and could be a limitation.

Private practice is far less common in Wales than some other areas of the UK (e.g. South of England). We have added this point to the discussion (line 422-425). When anti-depressants are initiated during an in-patient stay, the responsibility for continued prescribing is handed over the general practitioner on discharge. Therefore, these women will be identified.

*       Residual confounding; you allude to this, important confounders cannot be quantified but with GP data could be strengthened - e.g., family history of mental health illness and environment (household size, sibling diagnoses etc).

We did not have access to these specific variables but have now listed them (lines 415-417) as potential sources of residual confounding which you rightly say is a potential limitation of any observational study.


Any attachments provided with reviews can be seen via the following link:
--------------------------------------------------------- ---
General editorial requests:
*       We ask every co-author listed on the manuscript to fill in a contributing author statement, making sure to declare all competing interests. If any of the co-authors have not filled in the statement, we will remind them to do so when the paper is revised. If all statements are not completed in a timely fashion this could hold up the re-review process. If new competing interests are declared later in the revision process, this may also hold up the submission. Should there be a problem getting one of your co-authors to fill in a statement we will be in contact. Please do not add or remove authors without first discussing this with the handling editor. You can see our competing interests policy here: <https://eur03.safelinks.protection.outlook.com/?url=http%3A%2F%2Fjournals.plos.org%2Fplosmedicine%2Fs%2Fcompeting-interests&data=05%7C02%7CSaraid.McIlvride%40glasgow.ac.uk%7C382bfe7a54f54fe9875408ddfc350f09%7C6e725c29763a4f5081f22e254f0133c8%7C1%7C0%7C638944027062801499%7CUnknown%7CTWFpbGZsb3d8eyJFbXB0eU1hcGkiOnRydWUsIlYiOiIwLjAuMDAwMCIsIlAiOiJXaW4zMiIsIkFOIjoiTWFpbCIsIldUIjoyfQ%3D%3D%7C0%7C%7C%7C&sdata=D6Y2DPVpXUCj2eNKNMIgFrix55WDR5Qu9bEcHXPcV6E%3D&reserved=0>.

We have added a Competing Interests section to the manuscript.

*       Please upload any figures associated with your paper as individual TIF or EPS files with 300dpi resolution at resubmission; please read our figure guidelines for more information on our requirements: <https://eur03.safelinks.protection.outlook.com/?url=http%3A%2F%2Fjournals.plos.org%2Fplosmedicine%2Fs%2Ffigures&data=05%7C02%7CSaraid.McIlvride%40glasgow.ac.uk%7C382bfe7a54f54fe9875408ddfc350f09%7C6e725c29763a4f5081f22e254f0133c8%7C1%7C0%7C638944027062814266%7CUnknown%7CTWFpbGZsb3d8eyJFbXB0eU1hcGkiOnRydWUsIlYiOiIwLjAuMDAwMCIsIlAiOiJXaW4zMiIsIkFOIjoiTWFpbCIsIldUIjoyfQ%3D%3D%7C0%7C%7C%7C&sdata=S%2BWOuS8h7L3MuSeaEUjeEC2RD8jtVetkX00Woia%2FMlo%3D&reserved=0>. While revising your submission, we strongly recommend that you use PLOS's NAAS tool (<https://eur03.safelinks.protection.outlook.com/?url=https%3A%2F%2Fngplosjournals.pagemajik.ai%2Fartanalysis&data=05%7C02%7CSaraid.McIlvride%40glasgow.ac.uk%7C382bfe7a54f54fe9875408ddfc350f09%7C6e725c29763a4f5081f22e254f0133c8%7C1%7C0%7C638944027062826331%7CUnknown%7CTWFpbGZsb3d8eyJFbXB0eU1hcGkiOnRydWUsIlYiOiIwLjAuMDAwMCIsIlAiOiJXaW4zMiIsIkFOIjoiTWFpbCIsIldUIjoyfQ%3D%3D%7C0%7C%7C%7C&sdata=C8eudu5qtMpP9DlrBGUfo9QzuWsO7GIUqPUbn7SI%2Fto%3D&reserved=0>) to test your figure files. NAAS can convert your figure files to the TIFF file type and meet basic requirements (such as print size, resolution), or provide you with a report on issues that do not meet our requirements and that NAAS cannot fix.

After uploading your figures to PLOS's NAAS tool - <https://eur03.safelinks.protection.outlook.com/?url=https%3A%2F%2Fngplosjournals.pagemajik.ai%2Fartanalysis&data=05%7C02%7CSaraid.McIlvride%40glasgow.ac.uk%7C382bfe7a54f54fe9875408ddfc350f09%7C6e725c29763a4f5081f22e254f0133c8%7C1%7C0%7C638944027062838486%7CUnknown%7CTWFpbGZsb3d8eyJFbXB0eU1hcGkiOnRydWUsIlYiOiIwLjAuMDAwMCIsIlAiOiJXaW4zMiIsIkFOIjoiTWFpbCIsIldUIjoyfQ%3D%3D%7C0%7C%7C%7C&sdata=ypWwxl%2F6T5SanD8iJiEgEPnlIpG2nCuCpLGQf9xbtEw%3D&reserved=0>, NAAS will process the files provided and display the results in the "Uploaded Files" section of the page as the processing is complete.
If the uploaded figures meet our requirements (or NAAS is able to fix the files to meet our requirements), the figure will be marked as "fixed" above. If NAAS is unable to fix the files, a red "failed" label will appear above.
When NAAS has confirmed that the figure files meet our requirements, please download the file via the download option, and include these NAAS processed figure files when submitting your revised manuscript.
Done.
*       We expect all researchers with submissions to PLOS in which author-generated code underpins the findings in the manuscript to make all author-generated code available without restrictions upon publication of the work. In cases where code is central to the manuscript, we may require the code to be made available as a condition of publication. Authors are responsible for ensuring that the code is reusable and well documented. Please make any custom code available, either as part of your data deposition or as a supplementary file. Please add a sentence to your data availability statement regarding any code used in the study, e.g. "The code used in the analysis is available from Github [URL] and archived in Zenodo [DOI link]" Please review our guidelines at <https://eur03.safelinks.protection.outlook.com/?url=https%3A%2F%2Fjournals.plos.org%2Fplosmedicine%2Fs%2Fmaterials-software-and-code-sharing&data=05%7C02%7CSaraid.McIlvride%40glasgow.ac.uk%7C382bfe7a54f54fe9875408ddfc350f09%7C6e725c29763a4f5081f22e254f0133c8%7C1%7C0%7C638944027062850256%7CUnknown%7CTWFpbGZsb3d8eyJFbXB0eU1hcGkiOnRydWUsIlYiOiIwLjAuMDAwMCIsIlAiOiJXaW4zMiIsIkFOIjoiTWFpbCIsIldUIjoyfQ%3D%3D%7C0%7C%7C%7C&sdata=4ykRtcQowKoP3QDf1GgXoMaMUKQivqj2SlEE%2Br3wgIk%3D&reserved=0> and ensure that your code is shared in a way that follows best practice and facilitates reproducibility and reuse. Because Github depositions can
be readily changed or deleted, we encourage you to make a permanent DOI'd copy (e.g. in Zenodo) and provide the URL.

The code used in the main analyses has been uploaded to github (<https://github.com/smc25/antideps_code.git>) and archived in Zenodo (<https://doi.org/10.5281/zenodo.18337551>).
*       Please ensure that the study is reported according to the STROBE guideline and include the completed STROBE checklist as Supporting Information. When completing the checklist, please use section and paragraph numbers, rather than page numbers. Please add the following statement, or similar, to the Methods: "This study is reported as per STROBE guideline (S1 Checklist)."

A completed Strobe checklist has been added to the supporting information (S1 Checklist).

FORMATTING - GENERAL

*       At this stage, we ask that you include a short, non-technical Author Summary of your research to make findings accessible to a wide audience that includes both scientists and non-scientists. The Author Summary should immediately follow the Abstract in your revised manuscript. This text is subject to editorial change and should be distinct from the scientific abstract. Ideally each sub-heading should contain 2-3 single sentence, concise bullet points containing the most salient points from your study. In the final bullet point of 'What Do These Findings Mean?', please include the main limitations of the study in non-technical language. Please see our author guidelines for more information: <https://eur03.safelinks.protection.outlook.com/?url=https%3A%2F%2Fjournals.plos.org%2Fplosmedicine%2Fs%2Frevising-your-manuscript%23loc-author-summary&data=05%7C02%7CSaraid.McIlvride%40glasgow.ac.uk%7C382bfe7a54f54fe9875408ddfc350f09%7C6e725c29763a4f5081f22e254f0133c8%7C1%7C0%7C638944027062867622%7CUnknown%7CTWFpbGZsb3d8eyJFbXB0eU1hcGkiOnRydWUsIlYiOiIwLjAuMDAwMCIsIlAiOiJXaW4zMiIsIkFOIjoiTWFpbCIsIldUIjoyfQ%3D%3D%7C0%7C%7C%7C&sdata=mxVGwigVaGgJwDIuANoD53xiS8r%2BFv8O%2BHHiV9DEu2E%3D&reserved=0>.

This has been inserted after the Abstract.

*       Please express the main results with 95% CIs as well as p values. When reporting p values please report as p<0.001 and where higher as the exact p value p=0.002, for example. Throughout, suggest reporting statistical information as follows to improve clarity for the reader "22% (95% CI [13%,28%]; p</=)". Please be sure to define all numerical values at first use.

This has been updated.

*       Please include page numbers and line numbers in the manuscript file. Use continuous line numbers (do not restart the numbering on each page).

Done.

*       Please cite the reference numbers in square brackets. Citations should precede punctuation. Done.

FIGURES AND TABLES

*      Please consider moving figure S1 to the main text.
We prefer to keep the current Figure 1 as the first figure in the main paper.

SUPPLEMENTARY MATERIAL
*       Please note that supplementary material will be posted as supplied by the authors. Therefore, please amend it according to the relevant comments outlined here.
Done.
*       Please cite your Supporting Information as outlined [here](https://eur03.safelinks.protection.outlook.com/?url=https%3A%2F%2Fjournals.plos.org%2Fplosmedicine%2Fs%2Fsupporting-information&data=05%7C02%7CSaraid.McIlvride%40glasgow.ac.uk%7C382bfe7a54f54fe9875408ddfc350f09%7C6e725c29763a4f5081f22e254f0133c8%7C1%7C0%7C638944027062885463%7CUnknown%7CTWFpbGZsb3d8eyJFbXB0eU1hcGkiOnRydWUsIlYiOiIwLjAuMDAwMCIsIlAiOiJXaW4zMiIsIkFOIjoiTWFpbCIsIldUIjoyfQ%3D%3D%7C0%7C%7C%7C&sdata=wsVIdrF5beFKXlH0Cw7jWvGonOO7S5175exCXynQJsE%3D&reserved=0)
Done.
REFERENCES
*       PLOS uses the numbered citation (citation-sequence) method and first six authors, et al.
Done.
*       Please ensure that journal name abbreviations match those found in the National Center for Biotechnology Information (NCBI) [databases](https://eur03.safelinks.protection.outlook.com/?url=http%3A%2F%2Fwww.ncbi.nlm.nih.gov%2Fnlmcatalog%2Fjournals&data=05%7C02%7CSaraid.McIlvride%40glasgow.ac.uk%7C382bfe7a54f54fe9875408ddfc350f09%7C6e725c29763a4f5081f22e254f0133c8%7C1%7C0%7C638944027062900837%7CUnknown%7CTWFpbGZsb3d8eyJFbXB0eU1hcGkiOnRydWUsIlYiOiIwLjAuMDAwMCIsIlAiOiJXaW4zMiIsIkFOIjoiTWFpbCIsIldUIjoyfQ%3D%3D%7C0%7C%7C%7C&sdata=2qk9iDS2%2FFbTz0poV9E4%2FWe4UgpNMY2Scj91jiEaLQ4%3D&reserved=0) and are appropriately formatted and capitalised.
Done.
*       Where website addresses are cited, please include the complete URL and specify the date of access (e.g. [accessed: 12/06/2023]).
Done.
*       Please also [see](https://eur03.safelinks.protection.outlook.com/?url=https%3A%2F%2Fjournals.plos.org%2Fplosmedicine%2Fs%2Fsubmission-guidelines%23loc-references&data=05%7C02%7CSaraid.McIlvride%40glasgow.ac.uk%7C382bfe7a54f54fe9875408ddfc350f09%7C6e725c29763a4f5081f22e254f0133c8%7C1%7C0%7C638944027062915376%7CUnknown%7CTWFpbGZsb3d8eyJFbXB0eU1hcGkiOnRydWUsIlYiOiIwLjAuMDAwMCIsIlAiOiJXaW4zMiIsIkFOIjoiTWFpbCIsIldUIjoyfQ%3D%3D%7C0%7C%7C%7C&sdata=t6CPjDTYReVytHbnjxc8DRQFfqerFQSWy8fYYmhbHpE%3D&reserved=0)  for further details on reference formatting.

OBSERVATIONAL STUDIES

*       Abstract: Please include the study design, population and setting, number of participants, years during which the study took place (enrollment and follow up), length of follow up, and main outcome measures.

Length of follow up has been added to the abstract.

*       Please ensure that the study is reported according to the STROBE (or appropriate STOBE extension) guideline (available [from](https://eur03.safelinks.protection.outlook.com/?url=https%3A%2F%2Fwww.equator-network.org%2Freporting-guidelines%2Fstrobe&data=05%7C02%7CSaraid.McIlvride%40glasgow.ac.uk%7C382bfe7a54f54fe9875408ddfc350f09%7C6e725c29763a4f5081f22e254f0133c8%7C1%7C0%7C638944027062929054%7CUnknown%7CTWFpbGZsb3d8eyJFbXB0eU1hcGkiOnRydWUsIlYiOiIwLjAuMDAwMCIsIlAiOiJXaW4zMiIsIkFOIjoiTWFpbCIsIldUIjoyfQ%3D%3D%7C0%7C%7C%7C&sdata=PUyxElEpwaDHoyVpPC7YLXURT%2FAd7HHL08ST2n0E2Uc%3D&reserved=0) and include the completed STROBE (or STROBE extension) checklist as Supporting Information. Please add the following statement, or similar, to the Methods: "This study is reported as per the Strengthening the Reporting of Observational Studies in Epidemiology (STROBE) guideline (S1 Checklist)." When completing the checklist, please use section and paragraph numbers, rather than page numbers.

A completed Strobe checklist has been added.

*       For all observational studies, in the manuscript text, please indicate: (1) the specific hypotheses you intended to test, (2) the analytical methods by which you planned to test them, (3) the analyses you actually performed, and (4) when reported analyses differ from those that were planned, transparent explanations for differences that affect the reliability of the study's results. If a reported analysis was performed based on an interesting but unanticipated pattern in the data, please be clear that the analysis was data driven.

The hypothesis has been added to the introduction (lines 89-91). Detail has been added to the methods to make clear which analyses were planned a priori (e.g. lines 190 onwards.)

*       Please state in the Methods section whether the study had a prospective protocol or analysis plan. If a prospective analysis plan (from your funding proposal, IRB or other ethics committee submission, study protocol, or other planning document written before analyzing the data) was used in designing the study, please include the relevant document(s) with your revised manuscript as a Supporting Information file to be published alongside your study and cite it in the Methods section. A legend for this file should be included at the end of your manuscript. If no such document exists, please make sure that the Methods section transparently describes when analyses were planned, and when/why any data-driven changes to analyses took place. Changes in the analysis, including those made in response to peer review comments, should be identified as such in the Methods section of the paper, with rationale.

A paragraph describing the planned analysis and changes has been added to the Methods section (line 190-205).
