# Supplementary material for: Prenatal exposure to maternal depression and antidepressants and neurodevelopmental outcomes: A population cohort study
Source: PLoS Med. 2026 Sep 29;23(9):e1004658. doi: 10.1371/journal.pmed.1004658 (PMC13623103; doi:10.1371/journal.pmed.1004658)
Supplement: Peer review history

# Editor report: Decision Letter 0

**DOI:** 10.1371/journal.pmed.1004658.r001

**Authors:** Louise Gaynor-Brook

30 May 2025

Dear Dr McIlvride,

Thank you for submitting your manuscript entitled "Prenatal exposure to maternal depression and antidepressants and neurodevelopmental outcomes: a population cohort study of 167,447 children" for consideration by PLOS Medicine.

Your manuscript has now been evaluated by the PLOS Medicine editorial staff as well as by an academic editor with relevant expertise and I am writing to let you know that we would like to send your submission out for external peer review.

However, before we can send your manuscript to reviewers, we need you to complete your submission by providing the metadata that is required for full assessment. To this end, please login to Editorial Manager where you will find the paper in the 'Submissions Needing Revisions' folder on your homepage. Please click 'Revise Submission' from the Action Links and complete all additional questions in the submission questionnaire.

For clinical studies, please upload a copy of your trial study protocol as a supporting information file. The study protocol should be the version submitted for approval to the institutional review board or ethics committee, should include any amendments to the study protocol, as well as the date of their approval by the institutional review or ethics committee. Please also detail any deviations from the study protocol in the Methods section of your manuscript. The editors will consider the protocol and study conduct prior to a final decision for external review.

Please re-submit your manuscript within two working days, i.e. by Jun 03 2025 11:59PM.

Login to Editorial Manager here: https://www.editorialmanager.com/pmedicine

Once your full submission is complete, your paper will undergo a series of checks in preparation for peer review. Once your manuscript has passed all checks it will be sent out for review.

Feel free to email us at plosmedicine@plos.org if you have any queries relating to your submission.

Kind regards,

Louise Gaynor-Brook, MBBS PhD

Senior Editor

PLOS Medicine

# Peer review: Decision Letter 1

**DOI:** 10.1371/journal.pmed.1004658.r002

**Authors:** Suzanne De Bruijn

25 Sep 2025

Dear Dr McIlvride,

Many thanks for submitting your manuscript "Prenatal exposure to maternal depression and antidepressants and neurodevelopmental outcomes: a population cohort study of 167,447 children" (PMEDICINE-D-25-01855R1) to PLOS Medicine. The paper has been reviewed by subject experts and a statistician; their comments are included below and can also be accessed here: [LINK]

As you will see, the reviewers think this is an important topic, but require clarifications regarding the analysis, as well as additional detail. Furthermore, the reviewers suggest that different exposures need to be analyses together to be able to draw comparative conclusions. After discussing the paper with the editorial team and an academic editor with relevant expertise, I'm pleased to invite you to revise the paper in response to the reviewers' comments. Please also address the editorial comments at the bottom of this email. We plan to send the revised paper to some or all of the original reviewers, and we cannot provide any guarantees at this stage regarding publication.

In addition to these revisions, you may need to complete some formatting changes, which you will receive in a follow up email. A member of our team will be in touch with a set of requests shortly. If you do not receive a separate email within a few days, please assume that checks have been completed, and no additional changes are required.

When you upload your revision, please include a point-by-point response that addresses all of the reviewer and editorial points, indicating the changes made in the manuscript and either an excerpt of the revised text or the location (eg: page and line number) where each change can be found. Please also be sure to check the general editorial comments at the end of this letter and include these in your point-by-point response. When you resubmit your paper, please include a clean version of the paper as the main article file and a version with changes tracked as a marked-up manuscript. It may also be helpful to check the guidelines for revised papers at http://journals.plos.org/plosmedicine/s/revising-your-manuscript for any that apply to your paper.

We ask that you submit your revision by Oct 16 2025 11:59PM. However, if this deadline is not feasible, please contact me by email, and we can discuss a suitable alternative.

Don't hesitate to contact me directly with any questions (sbruijn@plos.org).

Best regards,

Suzanne

Suzanne De Bruijn, PhD

Associate Editor

PLOS Medicine

sbruijn@plos.org

-----------------------------------------------------------

Comments from the academic editor:

This is an important topic, but the main analyses need to be re-designed and run again.

-----------------------------------------------------------

Comments from the reviewers:

Reviewer #1: This manuscript presents an analysis of data from 167,447 children born in Wales, between 2009 to 2016, investigating the impacts of maternal depression and maternal antidepressant prescription on special educational needs outcomes. My comments focus on the statistical aspects of this analysis. Given my interpretation of the statistical analysis as described in this manuscript, it is unclear whether the conclusions drawn are supported by the data.

1.Here there are two exposures of interest: one or more prescriptions for antidepressant medication during pregnancy or in the month prior to conception, and a maternal depression diagnosis recorded in the two years before delivery. Here it seems that separate analyses have been conducted for different combinations of these two exposures. I have questions and recommendations for analysis of these two exposures.

a.In Table 1, the number of women with depression is stated as 25631. I would expect that the number of women with depression would be equal to the number of women with untreated depression plus the number of women with treated depression. However, this does not add up to the number of women with treated depression (7005) plus the number with untreated depression (12630). In fact, 7005+12630 = 19635, almost 6000 fewer women than are in the depression group. I don't understand why there is this difference; can this be explained?

b.Separate analyses for each exposure of interest have been conducted (as far as I can tell). Instead of conducting separate analyses for each of the cohorts of interest, it would make more sense to fit a model for the outcome, including a binary term for "presence of depression" (I'll call this Dep), a binary term for "antidepressant prescription" (I'll call this Ant), and an interaction between the two. The effect "treated depression" (Dep=Yes, Ant=Yes), "Untreated depression" (Dep=Yes, Ant=No) can then be extracted directly. The effect of "Depression" can also be extracted or of "Antidepressant use" would then be obtained by marginalising over the effect of the other exposure; in Stata this would be achieved using the margins command. This approach allows direct comparison of the effects of different combinations of these two exposures. If separate regression models have been fit for different subgroups then the comparisons made between results for women with treated and untreated depression, for example, are inappropriate: it is difficult to determine whether the estimates for children exposed to treated depression differ to a large degree from those for children exposed to untreated depression if the estimates for each are based on different statistical models.

c.If analyses have been conducted separately for different exposures, then statements such as "associations were stronger for treated depression than untreated depression" may not be supported by the data.

d.If the above suggestion for analysis aligns with the analyses that were actually conducted, then this needs to be clarified in the Statistical analyses section.

e.The above suggestion does not require any change to Table 1 (although, as I note below, standardised differences rather than p-values should be used to compare groups; and as noted above there is a discrepancy in the sample sizes of the different groups)

2.It is stated that a GEE-based approach was used to account for repeated measures on the same child; but it is unclear to me what the timing of the primary outcome of interest actually is, and how the data were structured. That is, were outcomes available for each child in each year? Was the primary question of interest SEN by age 10 (or some other age)? I have difficulty determining what the actual research question of interest is here; this requires clear definition of the exposure (this seems clearly defined), the outcome (again, clearly defined), and also the time of the outcome (at 5 years of age? 10 years? Or the time to the occurrence of the outcome?) Please define the research question of interest clearly.

3.It is stated that maternal and child confounders were adjusted for. However, the child-level confounders included child age - child age at what event? This seems as though it could instead be on the causal pathway from exposure to outcome (e.g. the age of a child when they first have SENs could be impacted by maternal depression and use of antidepressants), so should not be adjusted for.

4.When it is stated that gestational age and birthweight were truncated, does this mean that babies with ages and weights outside this region were excluded? It would be inadvisable to modify the observed values of child characteristics.

5.Please provide some justification for the missing-treated-as-separate category approach used for the analyses.

6.Standardised differences would be more informative than hypothesis tests for determining which confounders have large differneces between exposure groups. The reason for this is that standardised differences provide some measure of the magnitude of differences, while, for a large enough sample, any difference between groups, no matter how tiny, will have a small p-value.

7.It is stated that the final stage of models included potential mediators - however, when including potential mediators, the research question being asked is no longer about the impact of an exposure on an outcome; questions of how the effect of that exposure is mediated by the outcome are being assessed, and methods appropriate for mediation analyses are required. If interest is in separating out direct and indirect effects of the exposures of interest, then a formal mediation analysis would be required.

8.How were the analyses for specific antidepressants conducted? Was "antidepressant" included as a categorical variable in the outcome regression models?

Reviewer #2: This paper addresses maternal mental health; a hugely important aspect of maternal health that is both under recognised and under researched. Well done to the authors on this population linkage study. This study adds valuable insight and may assist women and their care providers in weighing up treatment options to optimise pregnancy outcomes for each individual woman. However, there are several points that, in my opinion warrant further attention:

Major points:

1.Please report according to STROBE guidelines.

2.Can the authors please provide clearer justification for the choice of analysis (i.e. the use of several comparison groups)? The outline of the exposure groups does not include sufficient detail, and it is not clear enough that women and offspring could be included in more than one group. Further, it is confusing why the totals don't add up, for example, I would have thought that treated depression + untreated depression + comparison group = total cohort. This does not seem to be the case though. Further detail on the division of groups would improve clarity.

3.Can the authors please provide further detail on each of the databases included in the study? The purpose and coverage of each database, and what percentage of data collected are missing. The authors state that the primary care data are available for 83% of the population but that is all. Please elaborate.

4.In the results, can the authors please explain why the four exposed groups are not all represented in figure 1? Why is the depression cohort not shown?

5.I think that the discussion needs some work and would benefit from deeper interrogation of previous studies on this topic. There needs to be a more thorough discussion of this study in the context of other studies examining SEN and maternal mental health, with a clearer distinction between how this study divides exposures to maternal depression and/or antidepressant use in pregnancy.

6.While population wide observational studies are a vital method of examining perinatal exposures and longer-term outcomes, they have inherent limitations. The authors highlight that postnatal and paternal depression could also impact outcomes. This is a very important point that warrants further discussion. Unmeasured confounders and residual confounding due to imperfect measures are likely to be present. This is a well understood, unavoidable aspect of retrospective cohort studies, particularly of perinatal exposures. Despite this, discussion of this and potential impacts on findings are important. I recommend that the authors expand this point in the limitations section.

7.Our findings support the recommendation that SSRIs should be the first line therapy where antidepressants are indicated during pregnancy. I think this statement needs revising; it overstates findings. I would argue that the study examines the risk of SEN in exposure groups and therefore recommending medications that treat depression in not an accurate conclusion of this study.

Minor points:

1.Please provide further data on the handling of missing data. Was there a statistical analysis plan a priori, and did it specify how missing data would be dealt with?

2.Mothers were excluded if their primary care records had gaps of more than 30 days during this period (30 day maximum allows for transfer of records between primary care practices). Please clarify what this means and the potential impact that it has.

3.Was a drug category system used? Could the authors please provide more detail.

4.In the exposure to specific antidepressant section of the results, could the authors please include the absolute numbers of the exposed included in each analysis? e.g. (SNRIs; n, N, %,OR 2.00, 95% CI 1.47-2.71).

5.The authors state Some children may have been further exposed to medication postnatally via breastmilk, however studies have shown that women with depression, treated and untreated, have low breastfeeding initiation. I would recommend that the authors review this sentence and either provide a more balanced viewpoint on this point or revise the wording.

Reviewer #3: Thank you very much for the opportunity to review your very interesting work. This is an under-researched area of great clinical and social importance. The approach of using multiple linked, population-based, high-quality datasets is very powerful and well executed. The manuscript is very well written and easy to understand. I do have a few points I want to raise for further clarification:

Methods:

Inclusion Criteria and Definitions

*Given a long time-window of up to 2 years, was there an approach to assess/validate whether the mother still had depression (without antidepressants) during the pregnancy?

Covariates:

*Was there a reason as to why other maternal comorbidities (other than epilepsy) were not included as covariates (having diabetes is linked to SEN too)?

*Similarly for childhood comorbidities (trisomies, inherited genetic conditions, epilepsy)?

Analysis:

*For missing data (there is about 5-10% missingness according to your Table 1 in parity and APGAR scores), did you use complete case analysis? This should be stated.

Results:

*Can you clarify if the follow up of 5.68 years is from birth or from point of entry into the cohort/school year? The following sentence "The main cohort comprised 167,477 children, born in Wales between 2009 and 2016, with mean follow-up of 5.68 years (Table 1)." suggests its from birth and thus needs clarification.

*Table 1: Your missing % are stated in some variables (e.g., smoking) but not in others (e.g., parity). Is there a reason why they are not presented with %s throughout - could they all be included as an additional category (as per the comment under table 1, f)?

*Table 2: Are the number of observations greater than Table 1 due to repeated observations per child? Could this be clarified.

*Not sure if you can confidently conclude on comparisons across exposure categories (e.g., the anti-depressant category had a stronger association than the depressed-only category) as each exposure category is independently compared to the undepressed population and not to each other. Especially given the confidence intervals sometimes overlap (e.g., "The association with SEN was higher for… specifically amitriptyline, and serotonin-norepinephrine reuptake inhibitors (SNRIs; OR 2.00, 95% CI 1.47-2.71) than for SSRIs (OR 1.39, 95% CI 1.31-1.48)". Further analysis using a multinomial or ordinal logistic regression with repeated measures may be able to more directly answer this.

*Small point: in the following sentence, the untreated depression confidence intervals are missing : "behavioral-emotional-social difficulties (treated depression OR 1.80, 95% ci 1.67-1.95; untreated depression OR 1.55, 95% CI 1.55, 95% CI 1.43-1.67; Figure 1, Tables S2-4)"

Discussion:

*Ascertainment of depressed women during pregnancy: I think an important limitation of using healthcare records is that you may have pregnant women with depression in the community who do not present to healthcare services (this does tend to be biased towards ethnic minority groups not presenting to their healthcare providers)

*Ascertainment of antidepressants. Another limitation is that you are using the GP records to identify antidepressants, which is the most comprehensive way, but this may miss those who are prescribed privately or directly from hospital and could be a limitation.

*Residual confounding; you allude to this, important confounders cannot be quantified but with GP data could be strengthened - e.g., family history of mental health illness and environment (household size, sibling diagnoses etc).

Any attachments provided with reviews can be seen via the following link: [LINK]

--------------------------------------------------------- ---

General editorial requests:

*We ask every co-author listed on the manuscript to fill in a contributing author statement, making sure to declare all competing interests. If any of the co-authors have not filled in the statement, we will remind them to do so when the paper is revised. If all statements are not completed in a timely fashion this could hold up the re-review process. If new competing interests are declared later in the revision process, this may also hold up the submission. Should there be a problem getting one of your co-authors to fill in a statement we will be in contact. Please do not add or remove authors without first discussing this with the handling editor. You can see our competing interests policy here: http://journals.plos.org/plosmedicine/s/competing-interests.

*Please upload any figures associated with your paper as individual TIF or EPS files with 300dpi resolution at resubmission; please read our figure guidelines for more information on our requirements: http://journals.plos.org/plosmedicine/s/figures. While revising your submission, we strongly recommend that you use PLOS's NAAS tool (https://ngplosjournals.pagemajik.ai/artanalysis) to test your figure files. NAAS can convert your figure files to the TIFF file type and meet basic requirements (such as print size, resolution), or provide you with a report on issues that do not meet our requirements and that NAAS cannot fix.

After uploading your figures to PLOS's NAAS tool - https://ngplosjournals.pagemajik.ai/artanalysis, NAAS will process the files provided and display the results in the "Uploaded Files" section of the page as the processing is complete.

If the uploaded figures meet our requirements (or NAAS is able to fix the files to meet our requirements), the figure will be marked as "fixed" above. If NAAS is unable to fix the files, a red "failed" label will appear above.

When NAAS has confirmed that the figure files meet our requirements, please download the file via the download option, and include these NAAS processed figure files when submitting your revised manuscript.

*We expect all researchers with submissions to PLOS in which author-generated code underpins the findings in the manuscript to make all author-generated code available without restrictions upon publication of the work. In cases where code is central to the manuscript, we may require the code to be made available as a condition of publication. Authors are responsible for ensuring that the code is reusable and well documented. Please make any custom code available, either as part of your data deposition or as a supplementary file. Please add a sentence to your data availability statement regarding any code used in the study, e.g. "The code used in the analysis is available from Github [URL] and archived in Zenodo [DOI link]" Please review our guidelines at https://journals.plos.org/plosmedicine/s/materials-software-and-code-sharing and ensure that your code is shared in a way that follows best practice and facilitates reproducibility and reuse. Because Github depositions can be readily changed or deleted, we encourage you to make a permanent DOI'd copy (e.g. in Zenodo) and provide the URL.

*Please ensure that the study is reported according to the STROBE guideline and include the completed STROBE checklist as Supporting Information. When completing the checklist, please use section and paragraph numbers, rather than page numbers. Please add the following statement, or similar, to the Methods: "This study is reported as per STROBE guideline (S1 Checklist)."

FORMATTING - GENERAL

*At this stage, we ask that you include a short, non-technical Author Summary of your research to make findings accessible to a wide audience that includes both scientists and non-scientists. The Author Summary should immediately follow the Abstract in your revised manuscript. This text is subject to editorial change and should be distinct from the scientific abstract. Ideally each sub-heading should contain 2-3 single sentence, concise bullet points containing the most salient points from your study. In the final bullet point of 'What Do These Findings Mean?', please include the main limitations of the study in non-technical language. Please see our author guidelines for more information: https://journals.plos.org/plosmedicine/s/revising-your-manuscript#loc-author-summary.

*Please express the main results with 95% CIs as well as p values. When reporting p values please report as p<0.001 and where higher as the exact p value p=0.002, for example. Throughout, suggest reporting statistical information as follows to improve clarity for the reader "22% (95% CI [13%,28%]; p</=)". Please be sure to define all numerical values at first use.

*Please include page numbers and line numbers in the manuscript file. Use continuous line numbers (do not restart the numbering on each page).

*Please cite the reference numbers in square brackets. Citations should precede punctuation.

FIGURES AND TABLES

* Please consider moving figure S1 to the main text.

SUPPLEMENTARY MATERIAL

*Please note that supplementary material will be posted as supplied by the authors. Therefore, please amend it according to the relevant comments outlined here.

*Please cite your Supporting Information as outlined here: https://journals.plos.org/plosmedicine/s/supporting-information

REFERENCES

*PLOS uses the numbered citation (citation-sequence) method and first six authors, et al.

*Please ensure that journal name abbreviations match those found in the National Center for Biotechnology Information (NCBI) databases (http://www.ncbi.nlm.nih.gov/nlmcatalog/journals), and are appropriately formatted and capitalised.

*Where website addresses are cited, please include the complete URL and specify the date of access (e.g. [accessed: 12/06/2023]).

*Please also see https://journals.plos.org/plosmedicine/s/submission-guidelines#loc-references for further details on reference formatting.

OBSERVATIONAL STUDIES

*Abstract: Please include the study design, population and setting, number of participants, years during which the study took place (enrollment and follow up), length of follow up, and main outcome measures.

*Please ensure that the study is reported according to the STROBE (or appropriate STOBE extension) guideline (available from: https://www.equator-network.org/reporting-guidelines/strobe) and include the completed STROBE (or STROBE extension) checklist as Supporting Information. Please add the following statement, or similar, to the Methods: "This study is reported as per the Strengthening the Reporting of Observational Studies in Epidemiology (STROBE) guideline (S1 Checklist)." When completing the checklist, please use section and paragraph numbers, rather than page numbers.

*For all observational studies, in the manuscript text, please indicate: (1) the specific hypotheses you intended to test, (2) the analytical methods by which you planned to test them, (3) the analyses you actually performed, and (4) when reported analyses differ from those that were planned, transparent explanations for differences that affect the reliability of the study's results. If a reported analysis was performed based on an interesting but unanticipated pattern in the data, please be clear that the analysis was data driven.

*Please state in the Methods section whether the study had a prospective protocol or analysis plan. If a prospective analysis plan (from your funding proposal, IRB or other ethics committee submission, study protocol, or other planning document written before analyzing the data) was used in designing the study, please include the relevant document(s) with your revised manuscript as a Supporting Information file to be published alongside your study and cite it in the Methods section. A legend for this file should be included at the end of your manuscript. If no such document exists, please make sure that the Methods section transparently describes when analyses were planned, and when/why any data-driven changes to analyses took place. Changes in the analysis, including those made in response to peer review comments, should be identified as such in the Methods section of the paper, with rationale.

# Author comment: Author response to Decision Letter 2

**DOI:** 10.1371/journal.pmed.1004658.r003

26 Jan 2026

Attachment Submitted filename: Response to reviewers.docx

# Peer review: Decision Letter 2

**DOI:** 10.1371/journal.pmed.1004658.r004

**Authors:** Suzanne De Bruijn

6 Mar 2026

Dear Dr McIlvride,

Many thanks for submitting a revised version of your manuscript "Prenatal exposure to maternal depression and antidepressants and neurodevelopmental outcomes: a population cohort study of 167,447 children" (PMEDICINE-D-25-01855R2) to PLOS Medicine. The paper has been seen by the original reviewers; their comments are included below and can also be accessed here: [LINK]

As you will see, the reviewers think the manuscript has improved, but there are some serious concerns remaining, as pointed out by reviewer #1. Furthermore, both reviewer 1 and reviewer 3 state that the conclusions between groups cannot be made based on the main analyses, and that if you want to make these conclusions, this should be based on the results presented in table S5. After discussing the paper with the editorial team and an academic editor with relevant expertise, we are pleased to invite you to revise your paper to address these remaining points. We ask you to address all comments, but specifically the comments from reviewer #1. We plan to send the revised paper to some or all of the original reviewers, and we cannot provide any guarantees at this stage regarding publication.

In addition to these revisions, you may need to complete some formatting changes, which you will receive in a follow up email. A member of our team will be in touch with a set of requests shortly. If you do not receive a separate email within a few days, please assume that checks have been completed, and no additional changes are required.

When you upload your revision, please include a point-by-point response that addresses all of the reviewer and editorial points, indicating the changes made in the manuscript and either an excerpt of the revised text or the location (eg: page and line number) where each change can be found. Please also be sure to check the general editorial comments at the end of this letter and include these in your point-by-point response. When you resubmit your paper, please include a clean version of the paper as the main article file and a version with changes tracked as a marked-up manuscript. It may also be helpful to check the guidelines for revised papers at http://journals.plos.org/plosmedicine/s/revising-your-manuscript for any that apply to your paper.

We ask that you submit your revision by Mar 27 2026 11:59PM. However, if this deadline is not feasible, please contact me by email, and we can discuss a suitable alternative.

Don't hesitate to contact me directly with any questions (sbruijn@plos.org).

Best regards,

Suzanne

Suzanne De Bruijn, PhD

Associate Editor

PLOS Medicine

sbruijn@plos.org

-----------------------------------------------------------

Comments from the academic editor:

The main and essential points by Reviewer 1 are not addressed.

-----------------------------------------------------------

Comments from the reviewers:

Reviewer #1: I thank the authors for their responses to my comments. However, I still feel that there are some issues with the results that have been presented, and it is not clear that the conclusions are valid given the analyses that have been conducted.

1.I had requested clarity around the causal question being asked - in particular about the timing of the outcome. This hasn't really been provided: further description of the analysis method has been provided (which is appreciated), but the research question of interest remains opaque. What is the research question that these methods have been applied to address? The time frame for the outcome needs to be provided: e.g. any SEN etc diagnosed during a child's school years. Without a clear specification of the research question it is difficult to determine whether the research methods used to answer that question are appropriate.

2.The abstract states that "Untreated maternal depression was associated with higher risk of SEN … than unexposed children. Prenatal exposure to antidepressants and treated depression … were associated with even higher risks". Comparative statements such as these ("were associated with even higher risks") can only be made if the estimates all come from the same statistical model. However, I believe that these estimates come from fitting models to different subsets of the data. If the authors wish to compare results for these different exposure types, they must use the results of the analyses presented in Table S5 (the model including the interaction term - please note that I do not think testing the statistical significance of the interaction term is a useful thing to do). The treatment effects in different subgroups can be obtained as I noted in my previous review. A similar comment holds for the rest of the comparative statements in the Methods and findings section of the abstract.

3.Estimates presented in the author summary should be accompanied by confidence intervals.

4.It is stated that small p-values were used to justify the inclusion of variables in models as confounders, however, this is not the recommended approach for the selection of variables to include in regression models. Instead, the modern approach to the selection of variables for inclusion in regression models to adjust for recommends considering causal diagrams and confounding pathways (see e.g. https://doi.org/10.1002/sim.10244 and PMID: 30840181).

5.This is also related to my comment regarding inclusion of "potential mediators": something may still be a mediator (i.e. on the causal pathway between the exposure and outcome of interest) even if there is no significant attenuation of effect. Thus the analyses where "potential mediators" (which I take to mean variables that the authors may believe to be on the causal pathway) should not be conducted; I strongly recommend that these analyses be removed.

Reviewer #2: Again, I would like to congratulate the authors on a well-conducted study addressing an important knowledge gap. The authors made some changes to the manuscript that improved the clarity. However, while some of the previous comments were addressed, there are still points that, in my opinion, require further attention.

1.In the abstract I don't think that there is adequate description of the methods.

2.There is inadequate description of the method of handling missing data in the methods section. The authors state that complete case analysis was sued but without justification for this.

3.Table 1 could be improved by adding a row above the categories of exposed and then for unexposed and total, to make it explicitly clear that there are multiple exposure groups.

4.For example, in line 247 in the tracked changes manuscript adjusted has been added to the odds ratio but the abbreviation still says OR. Using aOR for this and all adjusted analyses would improve clarity.

5.Line 254 states Results are for repeated observations per child across years under the table. I am unclear on what this means, could the authors please provide further clarification.

6.From the other reviewer comments and author reply, I understand why p-values have been included in table 1. I am unclear however why they have been used in the main analysis continued throughout the paper, when 95% CI are reported. Can the authors please provide further justification for this?

7.From the description in the methods, I understand that all comparisons are made to the unexposed group, however from line 260 onwards in the results section, it is unclear what the comparison group is. I think it reads as though the women with depression have been compared with women with untreated depression. Can the authors please revise the wording in the results to make this distinction clearer?

8.Discussion: I thank the authors for addressing my comments about placing their study in context of the wider literature on the topic, however, I think this still requires further attention in the most recent draft. Would the authors consider being more specific with comparisons to other studies? There are recent studies that examine the association between antidepressant exposure in pregnancy and offspring neurodevelopmental outcomes, with several employing multiple subgroup approaches such as sibling-comparison analyses and not simply univariate analysis. I don't feel that the discussion in its current form addresses these adequately.

Reviewer #3: Thank you very much for your response. The only point that still needs to be reviewed is the conclusions made in between groups [i.e., Figure 1]. I still think it is not fully accurate to make conclusions about comparisons between depressed untreated and treated when you are only comparing 1) depressed untreated vs. unexposed and 2) depressed treated vs. unexposed in your analyses. The analyses you have added to S5 would be more appropriate for your main findings if you want to conclude on these specific comparisons between treated/untreated.

Any attachments provided with reviews can be seen via the following link: [LINK]

# Author comment: Author response to Decision Letter 3

**DOI:** 10.1371/journal.pmed.1004658.r005

2 Jul 2026

Attachment Submitted filename: Response to reviewers.pdf

# Peer review: Decision Letter 3

**DOI:** 10.1371/journal.pmed.1004658.r006

**Authors:** Suzanne De Bruijn

4 Aug 2026

Dear Dr. McIlvride,

Thank you very much for re-submitting your manuscript "Prenatal exposure to maternal depression and antidepressants and neurodevelopmental outcomes: a population cohort study of 167,447 children" (PMEDICINE-D-25-01855R3) for review by PLOS Medicine.

I have discussed the paper with my colleagues and the academic editor and it was also seen again by three reviewers. I am pleased to say that provided the remaining minor comments from the reviewers, as well as the editorial and production issues are dealt with we are planning to accept the paper for publication in the journal.

The remaining issues that need to be addressed are listed at the end of this email. Any accompanying reviewer attachments can be seen via the link below. Please take these into account before resubmitting your manuscript:

[LINK]

***Please note while forming your response, if your article is accepted, you may have the opportunity to make the peer review history publicly available. The record will include editor decision letters (with reviews) and your responses to reviewer comments. If eligible, we will contact you to opt in or out.***

In revising the manuscript for further consideration here, please ensure you address the specific points made by each reviewer and the editors. In your rebuttal letter you should indicate your response to the reviewers' and editors' comments and the changes you have made in the manuscript. Please submit a clean version of the paper as the main article file. A version with changes marked must also be uploaded as a marked up manuscript file.

Please also check the guidelines for revised papers at http://journals.plos.org/plosmedicine/s/revising-your-manuscript for any that apply to your paper. If you haven't already, we ask that you provide a short, non-technical Author Summary of your research to make findings accessible to a wide audience that includes both scientists and non-scientists. The Author Summary should immediately follow the Abstract in your revised manuscript. This text is subject to editorial change and should be distinct from the scientific abstract.

In addition to these revisions, you may need to complete some formatting changes, which you will receive in a follow up email. A member of our team will be in touch with a set of requests shortly. If you do not receive a separate email within a few days, please assume that checks have been completed, and no additional changes are required.

We expect to receive your revised manuscript within 1 week. Please email us (plosmedicine@plos.org) if you have any questions or concerns.

Please review your reference list to ensure that it is complete and correct. If you have cited papers that have been retracted, please include the rationale for doing so in the manuscript text, or remove these references and replace them with relevant current references. Any changes to the reference list should be mentioned in the rebuttal letter that accompanies your revised manuscript.

Please note, when your manuscript is accepted, an uncorrected proof of your manuscript will be published online ahead of the final version, unless you've already opted out via the online submission form. If, for any reason, you do not want an earlier version of your manuscript published online or are unsure if you have already indicated as such, please let the journal staff know immediately at plosmedicine@plos.org.

If you have any questions in the meantime, please contact me or the journal staff on plosmedicine@plos.org.

We look forward to receiving the revised manuscript by Aug 11 2026 11:59PM.

Sincerely,

Suzanne De Bruijn, PhD

Associate Editor

PLOS Medicine

plosmedicine.org

------------------------------------------------------------

Requests from Editors:

GENERAL EDITORIAL REQUESTS

* Please confirm that your title complies with PLOS Medicine's style. Your title must be nondeclarative and not a question. It should begin with main concept if possible. "Effect of" should be used only if causality can be inferred, i.e., for an RCT. Please place the study design ("A randomized controlled trial," "A retrospective study," "A modelling study," etc.) in the subtitle (ie, after a colon).

*Please change your title to: “Prenatal exposure to maternal depression and antidepressants and neurodevelopmental outcomes: a population cohort study”

*Funding statement: thank you for including your funding statement. For HDR, please consider whether all the stated sources of funding are necessary to mention for this study.

*Please ensure that your Data availability statement in the metadata is the same as in the manuscript.

*ETHICS: Thank you for stating that “Access to the data was approved by the SAIL Databank Information Governance Review Panel (IGRP).” Please also provide an approval number.

* ETHICS: Please specify whether informed consent was written or oral. Please ensure that the research complies with the PLOS policy in full: https://journals.plos.org/plosmedicine/s/human-subjects-research#loc-patient-privacy-and-informed-consent-for-publication

ABSTRACT

* Please confirm that your abstract complies with our requirements, including format (three sections: Background, Methods and Findings, and Conclusions) and providing all the information relevant to this study type https://journals.plos.org/plosmedicine/s/submission-guidelines#loc-abstract

* In the last sentence of the Abstract Methods and Findings section, please describe the main limitation(s) of the study's methodology.

* In the abstract, please include the important dependent variables that are adjusted for in the analyses.

AUTHOR SUMMARY:

-Why was this study done: we suggest to combine the 1st and 2nd bullet point.

-Why was this study done, 3rd bullet point: we suggest to either remove this, or combine with bullet point 4.

-What did the researchers do and find: we suggest to combine bullet point 1 and 2.

* In the author summary, in the final bullet point of 'What Do These Findings Mean?', please include the main limitations of the study in non-technical language.

GENERAL

* Please ensure that the Introduction ends with a clear description of the study question or hypothesis.

* Please ensure that all abbreviations are defined at first use throughout the text.

* Please confirm that all numbers presented in the abstract are present and identical to numbers presented in the main manuscript text.

* Please include a statement regarding use or non-use of AI tools and technologies at the end of the Methods. Please specify the name(s) and version(s) of any tool(s) used and a description of their use, including how the AI-assisted or generated content was evaluated by authors, and a clear statement of which aspects of the study, article contents, data, or supporting files were affected or generated by AI tool usage. Please also include a statement specifying who retains responsibility for the final manuscript. See our guidelines for more information: https://journals.plos.org/plosmedicine/s/ethical-publishing-practice#loc-artificial-intelligence-tools-and-technologies.

* Statistical reporting: Please revise throughout the manuscript, including tables and figures.

- Please report statistical information as follows to improve clarity for the reader ""22% (95% CI [13,28]; p</=)"".

- Please separate upper and lower bounds with commas instead of hyphens as the latter can be confused with reporting of negative values.

- Please repeat statistical definitions (HR, CI etc.) for each set of parentheses.

* Please remove the 'conclusions' subheading from the discussion. Please also remove any other subheadings from the discussion.

* Thank you for including the STROBE checklist. However, could you attach the checklist as a separate file, rather than as part of the appendix?

FIGURES AND TABLES

* Please provide the unadjusted comparisons as well as the adjusted comparisons in all relevant Tables.

* Please specify the variables controlled for in all relevant Tables.

OBSERVATIONAL, COHORT, CROSS-SECTIONAL, AND CASE CONTROL STUDIES

* Did your study have a prospective protocol or analysis plan? Please state this (either way) early in the Methods section.

a) If a prospective analysis plan (from your funding proposal, IRB or other ethics committee submission, study protocol, or other planning document written before analyzing the data) was used in designing the study, please include the relevant prospectively written document with your revised manuscript as a Supporting Information file to be published alongside your study, and cite it in the Methods section. A legend for this file should be included at the end of your manuscript.

b) If no such document exists, please make sure that the Methods section transparently describes when analyses were planned, and when/why any data-driven changes to analyses took place.

c) In either case, changes in the analysis-- including those made in response to peer review comments-- should be identified as such in the Methods section of the paper, with rationale.

Comments from Reviewers:

Reviewer #1: I thank the authors for their responses to my comments. I have no further suggestions.

Reviewer #2: Thank you to the authors for addressing the concerns raised by the reviewers. I think that the study is much clearer now and that the design and analysis more logical following the last review. My recommendation is to publish the manuscript. Well done to the authorship team for the work that has gone into this study. I think that it adds evidence to an important aspect of maternity care that is often neglected.

The only minor suggestion that I have is that in the abstract, line 72 be revised. I think that saying 2.9% is unclear what effect estimate this is and therefore what is meant by it.

Reviewer #3: Thank you all my queries have been addressed.

Any attachments provided with reviews can be seen via the following link:

[LINK]

# Author comment: Author response to Decision Letter 4

**DOI:** 10.1371/journal.pmed.1004658.r007

13 Aug 2026

Attachment Submitted filename: Author reply.pdf

# Editor report: Decision Letter 4

**DOI:** 10.1371/journal.pmed.1004658.r008

**Authors:** Suzanne De Bruijn

17 Aug 2026

Dear Dr McIlvride,

On behalf of Suzanne de Bruijn, my colleagues and the Academic Editor, Lars Åke Persson, I am pleased to inform you that we have agreed to publish your manuscript "Prenatal exposure to maternal depression and antidepressants and neurodevelopmental outcomes: a population cohort study." (PMEDICINE-D-25-01855R4) in PLOS Medicine.

Before your manuscript can be formally accepted you will need to complete some formatting changes, which you will receive in a follow up email. Please be aware that it may take several days for you to receive this email; during this time no action is required by you. Once you have received these formatting requests, please note that your manuscript will not be scheduled for publication until you have made the required changes.

Please also note the following in Table 2: last row, please clarify why there is a number sign, "Depression#" and define in the legend or remove.

In the meantime, please log into Editorial Manager at http://www.editorialmanager.com/pmedicine/, click the "Update My Information" link at the top of the page, and update your user information to ensure an efficient production process.

PRESS

We frequently collaborate with press offices. If your institution or institutions have a press office, please notify them about your upcoming paper at this point, to enable them to help maximise its impact. If the press office is planning to promote your findings, we would be grateful if they could coordinate with medicinepress@plos.org. If you have not yet opted out of the early version process, we ask that you notify us immediately of any press plans so that we may do so on your behalf.

We also ask that you take this opportunity to read our Embargo Policy regarding the discussion, promotion and media coverage of work that is yet to be published by PLOS. As your manuscript is not yet published, it is bound by the conditions of our Embargo Policy. Please be aware that this policy is in place both to ensure that any press coverage of your article is fully substantiated and to provide a direct link between such coverage and the published work. For full details of our Embargo Policy, please visit http://www.plos.org/about/media-inquiries/embargo-policy/.

To enhance the reproducibility of your results, we recommend that you deposit your laboratory protocols in protocols.io, where a protocol can be assigned its own identifier (DOI) such that it can be cited independently in the future. Additionally, PLOS ONE offers an option to publish peer-reviewed clinical study protocols. Read more information on sharing protocols at https://plos.org/protocols?utm_medium=editorial-email&utm_source=authorletters&utm_campaign=protocols

Thank you again for submitting to PLOS Medicine. We look forward to publishing your paper.

Sincerely,

Alison Farrell, PhD

Senior Editor

PLOS Medicine
